# Integrated Delivery of Antiretroviral Treatment and Pre-exposure Prophylaxis to HIV-1–Serodiscordant Couples: A Prospective Implementation Study in Kenya and Uganda

**DOI:** 10.1371/journal.pmed.1002099

**Published:** 2016-08-23

**Authors:** Jared M. Baeten, Renee Heffron, Lara Kidoguchi, Nelly R. Mugo, Elly Katabira, Elizabeth A. Bukusi, Stephen Asiimwe, Jessica E. Haberer, Jennifer Morton, Kenneth Ngure, Nulu Bulya, Josephine Odoyo, Edna Tindimwebwa, Craig Hendrix, Mark A. Marzinke, Norma C. Ware, Monique A. Wyatt, Susan Morrison, Harald Haugen, Andrew Mujugira, Deborah Donnell, Connie Celum, Partners Demonstration Project Team

**Affiliations:** 1 Department of Global Health, University of Washington, Seattle, Seattle, Washington, United States of America; 2 Department of Medicine, University of Washington, Seattle, Seattle, Washington, United States of America; 3 Department of Epidemiology, University of Washington, Seattle, Seattle, Washington, United States of America; 4 Centre for Clinical Research, Kenya Medical Research Institute, Nairobi, Kenya; 5 Infectious Disease Institute, Makerere University, Kampala, Uganda; 6 Centre for Microbiology Research, Kenya Medical Research Institute, Nairobi, Kenya; 7 Kabwohe Clinical Research Center, Kabwohe, Uganda; 8 Massachusetts General Hospital, Boston, Massachusetts, United States of America; 9 College of Health Sciences, Jomo Kenyatta University of Agriculture and Technology, Nairobi, Kenya; 10 Department of Medicine, Johns Hopkins University, Baltimore, Maryland, United States of America; 11 Department of Global Health and Social Medicine, Harvard Medical School, Boston, Massachusetts, United States of America; 12 Harvard Global, Cambridge, Massachusetts, United States of America; 13 Vaccine and Infectious Disease Division, Fred Hutchinson Cancer Research Center, Seattle, Washington, United States of America; Medical Research Council South Africa, SOUTH AFRICA

## Abstract

**Background:**

Antiretroviral-based interventions for HIV-1 prevention, including antiretroviral therapy (ART) to reduce the infectiousness of HIV-1 infected persons and pre-exposure prophylaxis (PrEP) to reduce the susceptibility of HIV-1 uninfected persons, showed high efficacy for HIV-1 protection in randomized clinical trials. We conducted a prospective implementation study to understand the feasibility and effectiveness of these interventions in delivery settings.

**Methods and Findings:**

Between November 5, 2012, and January 5, 2015, we enrolled and followed 1,013 heterosexual HIV-1-serodiscordant couples in Kenya and Uganda in a prospective implementation study. ART and PrEP were offered through a pragmatic strategy, with ART promoted for all couples and PrEP offered until 6 mo after ART initiation by the HIV-1 infected partner, permitting time to achieve virologic suppression. One thousand thirteen couples were enrolled, 78% of partnerships initiated ART, and 97% used PrEP, during a median follow-up of 0.9 years. Objective measures of adherence to both prevention strategies demonstrated high use (≥85%). Given the low HIV-1 incidence observed in the study, an additional analysis was added to compare observed incidence to incidence estimated under a simulated counterfactual model constructed using data from a prior prospective study of HIV-1-serodiscordant couples. Counterfactual simulations predicted 39.7 HIV-1 infections would be expected in the population at an incidence of 5.2 per 100 person-years (95% CI 3.7–6.9). However, only two incident HIV-1 infections were observed, at an incidence of 0.2 per 100 person-years (95% CI 0.0–0.9, *p* < 0.0001 versus predicted). The use of a non-concurrent comparison of HIV-1 incidence is a potential limitation of this approach; however, it would not have been ethical to enroll a contemporaneous population not provided access to ART and PrEP.

**Conclusions:**

Integrated delivery of time-limited PrEP until sustained ART use in African HIV-1-serodiscordant couples was feasible, demonstrated high uptake and adherence, and resulted in near elimination of HIV-1 transmission, with an observed HIV incidence of <0.5% per year compared to an expected incidence of >5% per year.

## Abstract 2

In a prospective implementation study, Jared Baeten and colleagues investigate integrated delivery of antiretroviral treatment and pre-exposure prophylaxis to HIV-1–serodiscordant couples in Kenya and Uganda.

## Author Summary

**Why Was This Study Done?:**

- Each year, approximately 2 million people become infected with HIV-1, most through sexual transmission and a majority in Africa.
- Antiretroviral medications effectively treat HIV-1 infection, and, in the last 5 y, pivotal clinical trials demonstrated that such medications can be used to prevent new infections as well, through treatment of HIV-1 infected persons to reduce their infectiousness and use as pre-exposure prophylaxis by HIV-1 uninfected persons to reduce their susceptibility.
- Models for effective delivery of antiretroviral medications for HIV-1 prevention in Africa have not been evaluated.

**What Did We Do and Find?:**

- We conducted a prospective study of a novel delivery model for use of antiretroviral medications for HIV-1 prevention among just over 1,000 HIV-1-serodiscordant couples (i.e., couples in which one member was HIV-1 infected and the other uninfected) in Kenya and Uganda; couples had behavioral and biologic characteristics that put them at particularly enhanced risk of HIV-1, even compared with HIV-1-serodiscordant couples in general.
- Both members of all couples were offered antiretroviral medications, and they were counseled on their HIV-1 prevention benefits; the study took a pragmatic and cost-minimizing approach, discontinuing the use of pre-exposure prophylaxis for the uninfected partner 6 mo after the infected partner initiated antiretroviral treatment.
- The primary goals of the project were to evaluate the implementation of the delivery model, including uptake, adherence, continuation, and safety.
- Part-way through the anticipated delivery period, it became clear that the approach was highly successful and that HIV-1 transmission rates were considerably lower than would have been anticipated (a 96% reduction compared to expected rates in a simulated model).

**What Do These Findings Mean?:**

- Antiretroviral medications have proven to be a powerful tool for preventing HIV-1 spread in clinical research studies; the results of this study show that a practical delivery approach in an implementation setting is feasible with high uptake and adherence and can virtually eliminate HIV1- transmission.
- Wide-scale roll-out of use of antiretroviral medications for HIV-1 prevention could have substantial effects in reducing the global burden of new HIV-1 infections.

## Introduction

Antiretroviral medications markedly improve the survival of persons with HIV-1 infection and are the cornerstone intervention for the prevention of HIV-1 transmission from mother to child. Recent randomized trials have demonstrated that antiretroviral medications can also be used for the prevention of sexual HIV-1 transmission between adults, as antiretroviral treatment (ART) to reduce the infectiousness of HIV-1-infected persons and as pre-exposure prophylaxis (PrEP) for HIV-1-uninfected persons at high risk for HIV-1 acquisition [1–3]. HIV-1 prevention efficacies in excess of 90% have been estimated for both ART and PrEP when used individually with high adherence, while low adherence substantially compromises the HIV-1 prevention benefits of both strategies [1,4,5]. Limited data are available to assess the feasibility and effectiveness of ART and PrEP in settings outside of clinical trials.

HIV-1-serodiscordant couples—i.e., in which one member is HIV-1 infected and the other uninfected—were a key population for the clinical trial evaluations of ART and PrEP for HIV-1 prevention [1,2]. Population data from Africa suggest that up to half or more of new infections occur within stable serodiscordant marital or cohabiting relationships [6], making serodiscordant couples a priority population for delivery of effective HIV-1 prevention strategies. World Health Organization (WHO) guidance recommends consideration of ART and PrEP for HIV-1 prevention for couples [7]. However, HIV-1 risk can be heterogeneous, even within at-risk populations such as HIV-1-serodiscordant couples; prioritizing the subset at highest risk of HIV-1 acquisition could maximize the cost-effectiveness of antiretroviral-based interventions for HIV-1 prevention [8,9]. In addition, as both ART and PrEP are potential prevention options for HIV-1-serodiscordant couples, approaches that integrate their delivery, and provide PrEP in a time-limited fashion, may be the most pragmatic strategy.

As ART and PrEP are new prevention strategies against HIV-1, implementation science research is needed to assess potential delivery approaches to achieve maximal individual and public health benefits. For HIV-1-serodiscordant couples, HIV-1 risk is sustained prior to and during the first approximately 6 mo after ART initiation by the HIV-1-infected partner, when viral suppression is typically achieved, after which risk appears to be virtually zero [1]. PrEP may thus offer substantial benefit prior to and during early ART. Moreover, potential uptake of and adherence to ART and PrEP for HIV-1 prevention in implementation settings is not fully known and may be limited by refusals or delays in ART initiation or insufficient adherence to ART and PrEP [4,5,10]. We conducted a prospective implementation study to understand the delivery feasibility and uptake of, as well as adherence to, an integrated package of ART and PrEP among high-risk heterosexual HIV-1-serodiscordant couples in Kenya and Uganda.

## Methods

### Ethics Statement

The study protocol was approved by the University of Washington Human Subjects Division and ethics review committees at each of the study sites (for Kabwohe and Kampala, Uganda, the National HIV/AIDS Research Committee of the Uganda National Council for Science and Technology; for Kisumu and Thika, Kenya, the Ethics Review Committee of the Kenya Medical Research Institute). All participants provided written informed consent in English or their local language. In addition, a Data Monitoring Committee, comprised of independent scientists from Kenya, Uganda, and the United States, was convened by the University of Washington and met every 6 mo to advise on the conduct of the study; the Committee advised that the data from the study be reported prior to all participants reaching 24 mo of follow-up due to a substantial effect on HIV-1 incidence.

### Study Population

Beginning November 5, 2012, heterosexual HIV-1-serodiscordant couples were enrolled in a prospective, open-label, implementation science-driven study of ART and PrEP for HIV-1 prevention (the Partners Demonstration Project, Clinicaltrials.gov NCT02775929). The overall goal was to evaluate a scalable, integrated, and pragmatic delivery approach for ART and time-limited PrEP, in combination with targeted counseling, brief adherence promotion, and frequency of follow-up designed to reflect approaches suitable for public health settings in East Africa. A sample size of 1,000 couples was chosen to provide a robust evaluation of the integrated ART and PrEP delivery strategy, across a diversity of clinical research sites. Couples were recruited using community outreach methods by four clinical care and research sites in Kenya (Kisumu and Thika) and Uganda (Kabwohe and Kampala). Recruitment strategies included working with voluntary counseling and testing centers, antenatal clinics and programs for prevention of mother-to-child HIV-1 transmission, referrals from HIV-1 care providers, including those performing testing of partners of known HIV-1 infected individuals engaged in HIV-1 care, and community promotion activities for couples’ testing.

Eligible couples were ≥18 y of age, sexually active, and intending to remain as a couple. At the time of enrollment, HIV-1 seronegative partners had never used PrEP, had normal renal function (defined as an estimated creatinine clearance ≥60 mL/min using the Cockcroft-Gault equation), were not infected with hepatitis B virus, and were not pregnant or breastfeeding. At enrollment, HIV-1 seropositive partners were not using ART; so as not to have the research process detract from immediate clinical need for ART, couples were excluded if the HIV-1-infected partner had WHO stage III or IV HIV-1 disease conditions. In addition, in order to recruit a population at higher risk for HIV-1 infection, a validated, empiric risk scoring tool was applied, and couples with a score ≥5 (out of a maximum of 12) were eligible for enrollment; in prior studies of HIV-1-serodiscordant couples, a score ≥5 was associated with an HIV-1 incidence in excess of 3%–4% per year [11]. For calculating the score, characteristics assessed at the time of screening included age of the HIV-1-uninfected partner, number of children in the partnership, circumcision status of HIV-1-uninfected men, whether the couple was cohabitating, whether the couple had had sex unprotected by a condom in the prior month, and the plasma HIV-1 RNA level in the HIV-1-infected partner. There was no obligation for couples to commit to initiating ART or PrEP as part of study eligibility. Ineligible couples were referred for standard of care HIV-1 prevention and treatment services.

### Provision of ART and PrEP

At enrollment, couples were counseled on the HIV-1 prevention benefits of immediate ART and PrEP. HIV-1-infected partners were advised to initiate ART according to national policies, which, for Kenya and Uganda, evolved early in the study period from recommending initiation at CD4 counts ≤350 cells/μL to initiation for all HIV-1 infected partners in HIV-1-serodiscordant relationships, regardless of CD4 count. ART was offered at the study site or by referral to another HIV-1 care center of their choice; nationally recommended ART regimens were used (preferred regimen: tenofovir disoproxil fumarate, lamivudine, and efavirenz, with zidovudine and nevirapine as alternative agents). HIV-1-uninfected partners were offered PrEP (combination emtricitabine/tenofovir disoproxil fumarate 200 mg/300 mg once daily), which was provided at the study sites, as PrEP was not available otherwise in Kenya and Uganda during the study period. PrEP was offered until the HIV-1-infected partner had been on ART for 6 mo (a strategy characterized as PrEP as a "bridge" to sustained ART and viral suppression within the partnered relationship). For couples in which the HIV-1-infected partner delayed or declined ART, the bridge period was extended until ART was initiated and sustained for 6 mo. On an individual basis, as determined by the clinical discretion of the PrEP prescriber—for example, in couples attempting conception—the duration of PrEP could be extended. The use of PrEP in the periconception period, even if ART had been used for >6 mo, was permitted because of uncertainty whether ART alone could provide complete protection during this vulnerable period when other prevention strategies, such as condoms, cannot be combined with antiretroviral-based prevention options, and when HIV-1 prevention would be of even greater importance to avoid transmission to an infant.

Couples returned for follow-up visits at 1 mo after enrollment and then quarterly for up to 24 mo. Visits included HIV-1 serologic testing for HIV-1-uninfected partners using serial HIV-1 rapid tests according to national algorithms for HIV-1 testing, HIV-1 primary care for HIV-1-infected partners, and brief adherence counseling for those on PrEP and ART. Visits also included risk reduction counseling, syndromic assessment and treatment for sexually transmitted infections, and referral for male circumcision for HIV-1 uninfected men. Pregnancy testing was performed when clinically indicated, and HIV-1-uninfected pregnant women were permitted, with additional counseling and consent regarding available data on the safety of PrEP in pregnancy, to continue PrEP. Serum creatinine testing was done for HIV-1-uninfected partners at 1 mo after enrollment and then every 6 mo, to monitor for renal safety of those on PrEP. CD4 counts were done every 6 mo for HIV-1-infected partners, as part of standard HIV-1 care services. Serious adverse events and events felt to be related to PrEP were recorded by the treating physician.

Initially-HIV-1 seronegative participants who had positive HIV-1 rapid test results had HIV-1 seroconversion confirmed by enzyme immunoassay and plasma HIV-1 RNA PCR and were permanently discontinued from PrEP. For all HIV-1 seroconverters, archived plasma samples from the enrollment visit were tested by HIV-1 RNA PCR, and those with detectable HIV-1 RNA, signifying seronegative acute HIV-1 infection, were assessed as having been infected prior to study initiation. HIV-1 resistance to antiretrovirals was assessed by standard consensus sequencing in those who acquired HIV-1, from a sample collected at the time seroconversion was first detected [2].

### Adherence Assessment

Plasma HIV-1 RNA quantification was performed every 6 mo for HIV-1-infected partners; results were used as part of clinical care but were not used for determining whether to discontinue PrEP use in the uninfected partner. Viral suppression was defined as HIV-1 RNA <400 copies/mL. For HIV-1-uninfected partners who chose to take PrEP, several measures of adherence were used. First, pill counts of returned, unused PrEP medication were conducted at each follow-up visit; these results were used in adherence counseling sessions. Second, PrEP medication was distributed in bottles with medication electronic monitoring system (MEMS) caps to electronically capture each date and time that their PrEP pill bottle was opened [12]; MEMS data were used only for measurement of adherence and were not incorporated into adherence counseling. Finally, plasma was collected and archived at each visit; in subjects who acquired HIV-1 and a randomly selected 15% subset of HIV-1-uninfected partners, detection of tenofovir in plasma was measured via ultra-performance liquid chromatographic-tandem mass spectrometric (LC-MS/MS), with a limit of quantification of 0.31 ng/mL [2]. As plasma tenofovir testing was performed on archived plasma samples tested in batch, results were not used in adherence counseling.

### Statistical Analysis

For the present analysis, data collected through January 5, 2015, were analyzed, and incident HIV-1 infection was defined as seroconversion, excluding cases subsequently found to be HIV-1-infected prior to study initiation. Descriptive statistics were used to characterize the cohort and describe uptake of and adherence to ART and PrEP.

Given the low HIV-1 incidence observed in the study, an analysis was planned after the study initiated to compare to an expected HIV-1 incidence in a simulated comparable at-risk population, using data from the placebo arm of the prior Partners PrEP Study, the earlier PrEP clinical trial conducted among HIV-1-serodiscordant couples in the same geography [2]. In that prior study, ART was recommended for HIV-1-infected partners whose CD4 counts declined to <350 cells/μL, consistent with national ART policies at the time; some of those who became eligible for ART delayed or declined therapy [10]. For the counterfactual model, a bootstrap resampling study was conducted; in each simulation, we constructed a bootstrap sample of 1,013 couples, with a distribution of empiric HIV-1 transmission risk scores and duration of follow-up to match those of the present study [11]. The mean number of HIV-1 infections expected in the counterfactual population was averaged over 10,000 bootstrap samples; a 95% confidence interval was defined by the 2.5th and 97.5th quantiles. The incidence rate ratio was computed comparing HIV-1 incidence in the present study to the mean counterfactual estimate; a 95% confidence interval was calculated using a Poisson distribution, and the *p*-value was estimated by assessing the frequency of a comparable number of infections in the bootstrapped sample. Additional models were constructed by gender of the HIV-1-uninfected partner and enrollment plasma HIV-1 RNA concentration of the HIV-1-infected partner to create estimates for each of these subgroups.

Analyses were conducted using SAS version 9.4 (SAS Institute).

## Results

### Study Participants and Follow-Up

**Fig 1:**
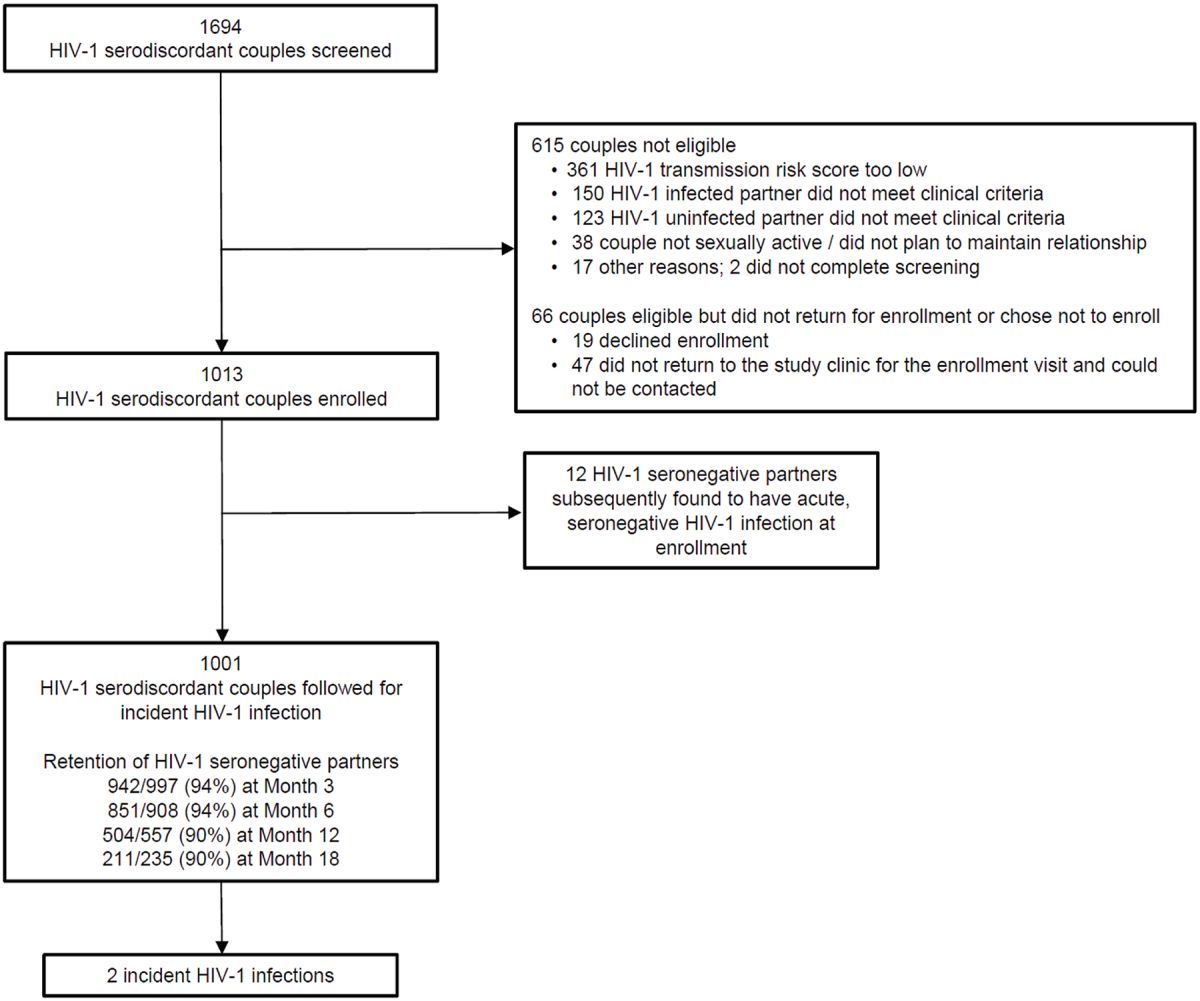
Screening, enrollment, and follow-up. Reasons for ineligibility are not mutually exclusive. A total of 1,013 higher-risk HIV-1-serodiscordant couples were enrolled. Of the initially HIV-1-seronegative partners, 12 subsequently seroconverted to HIV-1 but were found to have HIV-1 RNA in archived plasma from the time of enrollment, indicating acute seronegative HIV-1 infection.

Between November 5, 2012, and August 29, 2014, 1,694 couples were screened and 1,013 HIV-1-serodiscordant couples were enrolled (Fig 1). Sixty-six couples were eligible for enrollment but did not enroll; 19 of these declined enrollment, and 47 did not return to the study clinic for the enrollment visit and could not be contacted. Participant characteristics were consistent with elevated HIV-1 risk: 20% of HIV-1 uninfected partners were <25 y of age, more than half of couples had no children together, two-thirds practiced unprotected sex in the month prior to enrollment, and 41% of HIV-1-infected partners had a plasma HIV-1 RNA level >50,000 copies/mL (Table 1). Consistent with eligibility requirements, all couples had an empiric HIV-1 risk score ≥5, and nearly half (47%) had a score ≥7; the distribution of risk score components is detailed in Table 2. In addition, 41% of HIV-1 infected partners had a CD4 count >500 cells/μL and, thus, were not eligible for antiretroviral therapy except as part of an HIV-1-serodiscordant partnership, under the national antiretroviral therapy guidelines of Kenya and Uganda.

**Table 1:** Characteristics of the study subjects at enrollment (N = 1013 couples).

	Median (interquartile range) or *n* (%)
**Characteristics, HIV-1-uninfected partner**	
Male sex	679 (67%)
Age, years	30 (26,36)
Age <25 y	207 (20%)
Education, years	8 (7,12)
Monthly income, any	870 (86%)
Circumcised (men only)	454 (67%)
**Characteristics, HIV-1-infected partner**	
Age, years	28 (23,35)
Age <25 y	317 (31%)
Education, years	8 (6,11)
Monthly income, any	739 (73%)
CD4 cell count/μL	436 (272,638)
CD4 ≥500 cells/ μL	420 (41%)
HIV-1 plasma RNA, log_10_ copies/mL	4.6 (3.9, 5.0)
HIV-1 plasma RNA ≥50,000 copies/mL (4.7 log_10_ copies/mL)	417 (41%)
Time since HIV-1 diagnosis, days	37 (14, 256)
**Characteristics, couple**	
Married to each other	957 (94%)
Years living together	2.5 (0.8,7.0)
Years aware of HIV-1 serodiscordant status	0.1 (0.1,0.3)
Proportion without children	572 (56%)
Empiric HIV-1 transmission risk score*	
5	211 (21%)
6	323 (32%)
≥7	479 (47%)
**Sexual risk behavior**	
Number of sex acts, prior month	6 (3,11)
Any unprotected sex acts, prior month	656 (65%)
HIV-1 uninfected partner had any sex with outside partner, prior month	84 (8%)

* As previously described [11], an empiric risk scoring tool was developed and validated to identify highest-risk HIV-1-serodiscordant African heterosexual couples using data from >8,500 couples enrolled in three prospective studies. The score is composed of variables that are easily measurable in clinical settings: age of the HIV-1-uninfected partner (≤20 y = 4 points, 21–30 y = 1 point, >30 y = 0 points), the number of children in the partnership (0 = 4 points, 1–2 = 1 point, ≥3 = 0 points), circumcision status of HIV-1-uninfected male partners (uncircumcised = 1 point, circumcised = 0 points), marital/cohabitation status (married and/or cohabitating = 1 point, neither married nor cohabitating = 0 points), whether the couple had any unprotected sex in the prior month (yes = 2 points, no = 0 points), and plasma HIV-1 RNA levels in the HIV-1-infected partner (≥50,000 copies/mL = 3 points, 10,000–49,999 copies/mL = 1 point, <10,000 copies/mL = 0 points). The maximum score is 12, and a score of 0–2 has an anticipated HIV-1 incidence of <1% per year, a score of 3–4 has an anticipated incidence of approximately 2% per year, and a score ≥5 has an anticipated HIV-1 incidence of >3%–4% per year.

**Table 2:** Distribution of components of the couples’ HIV-1 empiric risk score.

Risk score element	Category	Score	*n* (%)
Age of HIV-1-uninfected partner	20 y or less	4	38 (4%)
	21–30 y	1	533 (53%)
	more than 30 y	0	442 (44%)
Number of children within the partnership	0	2	572 (56%)
	1–2	1	334 (33%)
	3 or more	0	107 (11%)
Male HIV-1-uninfected partner circumcision status	Yes	1	225 (22%)
	No, or HIV-1 uninfected partner is female	0	788 (78%)
Married and/or cohabiting	Yes	1	991 (98%)
	No	0	22 (2%)
Unprotected sex within partnership in 30 d prior to enrollment	Yes	2	948 (94%)
	No	0	65 (6%)
HIV-1-infected partner plasma HIV-1 RNA concentration (per mL)	50,000 copies or higher	3	417 (41%)
	10,000–49,999 copies	1	301 (30%)
	Fewer than 10,000 copies	0	295 (29%)

A total of 858 person-years of follow-up were accrued, with a median follow-up of 11.4 mo per couple for assessment of incident HIV-1 infection (interquartile range 6–15). Retention of HIV-1-uninfected partners for assessment of HIV-1 acquisition was ≥90% throughout follow-up. Pregnancy incidence was 24.9 and 18.5 per 100 woman-years in couples with HIV-1-uninfected and infected women, respectively. Adverse events were of the type and frequency expected for the population, and none were felt to be related to PrEP (S1 Table).

### Uptake and Adherence to ART and PrEP

At some point during follow-up, ART was initiated by 789 HIV-1-infected partners (78%), with the cumulative probability of initiating ART at 12% on the day of enrollment, 54% by 6 mo, 72% by 12 mo, 88% by 18 mo, and 92% by 24 mo post-enrollment. Among those who had initiated ART, plasma HIV-1 RNA was suppressed (<400 copies/mL) at the first measurement at least 6 mo after initiation in 89% (269/301).

Nearly all (960/1,013, 95%) HIV-1-uninfected partners initiated PrEP at enrollment, and an additional 25 (2%) initiated PrEP at a later visit, after enrollment. PrEP continuation was high: among those initiating at enrollment and attending the month 1 and 3 visits, 840 (97%) and 792 (94%) continued to receive PrEP. Adherence to PrEP, as measured by pill counts of returned, unused pills, indicated that 95% of dispensed pills had been taken as expected and 88% of periods between study visits had adherence ≥80%. By MEMS cap measurement, PrEP was taken on 82% of days, and 74% of periods between study visits had adherence ≥80%. In the randomly selected sample of individuals receiving PrEP (*n* = 133 subjects, at 438 study visits), tenofovir was detected in plasma in 85% of samples (372/438).

**Fig 2:**
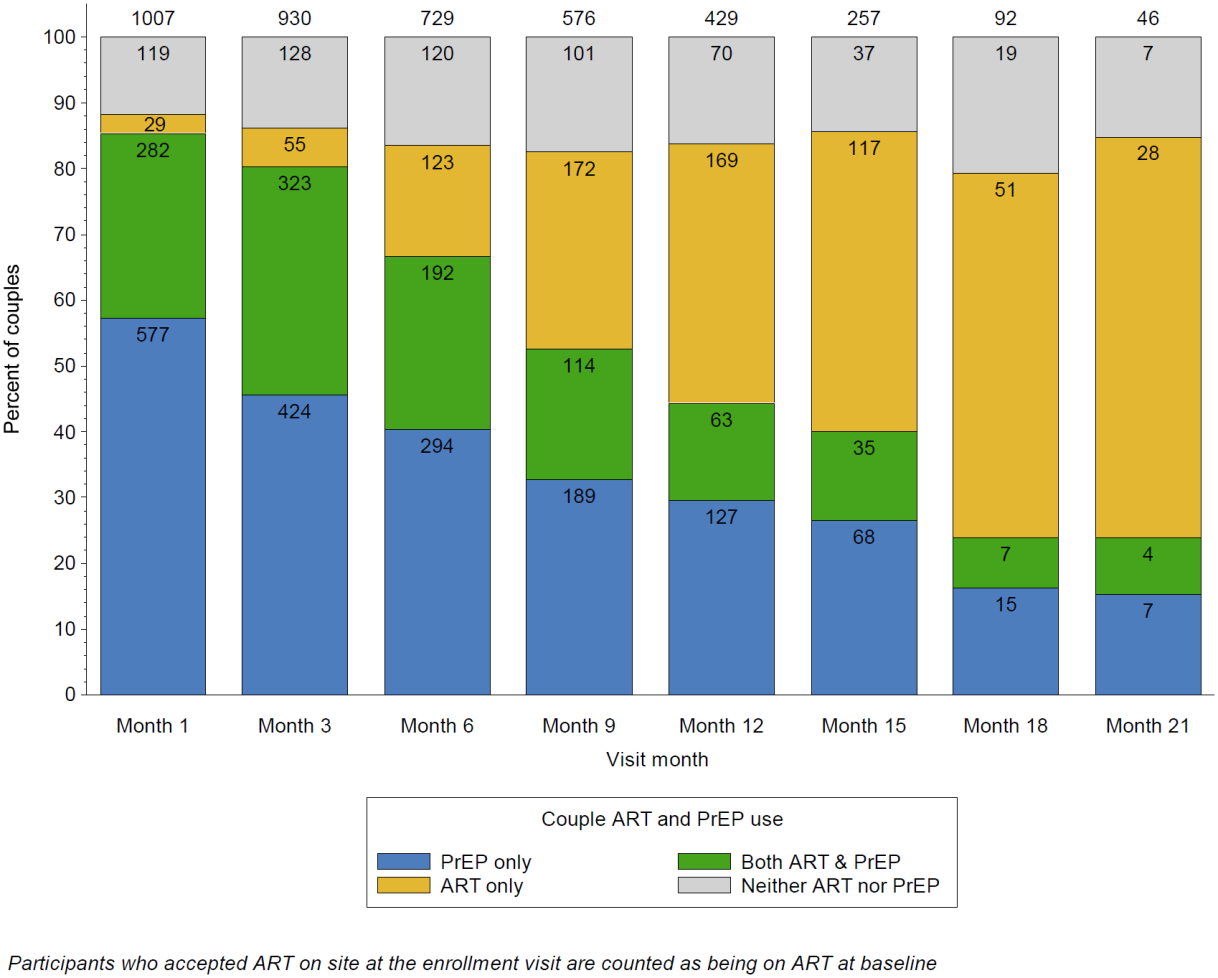
Couple ART and PrEP use over time. This graph illustrates the overall distribution of ART use by HIV-1-infected partners and PrEP use by HIV-1-uninfected partners within the study partnerships, over follow-up. The proportion of couples using only PrEP for HIV-1 prevention declined over time, as HIV-1-infected participants initiated ART, as defined by the PrEP as a bridge to ART approach of the project. Through month 6, there was the greatest overlap between ART and PrEP; thereafter, couples with HIV-1 infected partners that initiated ART at or soon after enrollment begin to discontinue PrEP. The primary reason for couples using neither ART nor PrEP was missed visits, which were considered as not exposed to PrEP (since PrEP was distributed only at the study sites during the study period) nor to ART (which was assumed to have not been initiated until first reported).

Thus, across all follow-up, couples used PrEP alone (i.e., prior to initiating ART) for 48% of person-time, both PrEP and ART (i.e., during the overlap bridge period) for 27%, and ART alone in 16% (Fig 2). Nine percent of follow-up person-time had neither ART nor PrEP use in the partnership, generally due to participant preferences or missed visits.

### Incident HIV-1 Infection and Counterfactual Comparison

**Fig 3:**
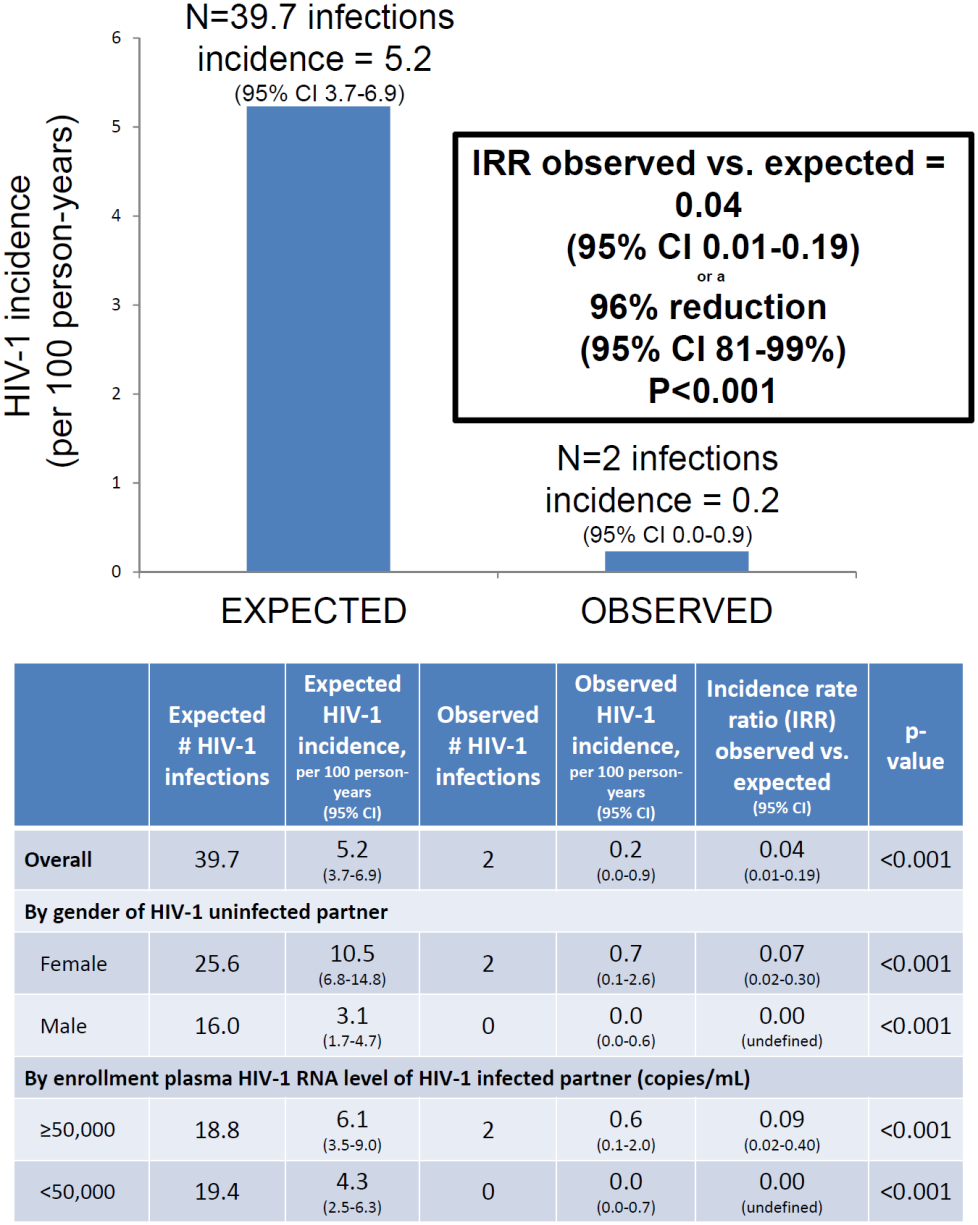
HIV-1 incidence, expected versus observed. Expected HIV-1 incidence was estimated from a counterfactual model, bootstrapping data from a comparable at-risk population of HIV-1-serodiscordant couples. The graphic presents results for the entire study population. The table details the overall population estimates as well as analyses stratified by gender of the HIV-1-uninfected partner and baseline plasma HIV-1 RNA concentrations of the HIV-1-infected partner.

A total of 14 initially HIV-1-seronegative partners seroconverted to HIV-1 during follow-up, of which 12 were subsequently determined by HIV-1 RNA PCR testing of archived plasma to have been infected at the time of study enrollment. Thus, two incident HIV-1 infections occurred, among 1,001 couples, for an observed HIV-1 incidence of 0.2 per 100 person-years (Fig 3). Given the risk score distribution of the enrolled population, the counterfactual simulations predicted that 39.7 HIV-1 infections (95% confidence interval 28–52) would be expected during follow-up in the population, at an overall HIV-1 incidence of 5.2 per 100 person-years. Thus, the observed HIV-1 incidence represented a 96% reduction (95% confidence interval 81%–99%, *p* < 0.001).

Both HIV-1 infections occurred in couples with HIV-1-uninfected female partners. When stratified by gender, substantial reductions in observed compared to expected HIV-1 infections were seen both in couples with HIV-1-uninfected female partners (93% reduction in HIV-1 incidence, *p* < 0.001) and couples with HIV-1-uninfected male partners (100% reduction in HIV-1 incidence, *p* < 0.001). Similarly, HIV-1 risk reduction was >90% both in couples in which the HIV-1-infected partner had plasma HIV-1 RNA concentrations ≥50,000 and <50,000 copies/mL.

The two HIV-1 infections both appeared to occur in the absence of use of ART or PrEP. The first occurred 15 mo after enrollment, and the uninfected partner had no tenofovir detected in plasma from that visit; the couple had separated by month 3, and the uninfected partner did not report sex within the relationship after the separation but reported a new sexual partner. The second occurred 3 mo after enrollment; no tenofovir was detected in plasma from that visit, and the infected partner had not yet initiated ART; the initially uninfected partner reported several breaks in PrEP use due in part to travel, illness, separations from the infected partner, and intimate partner violence in the relationship. Neither seroconverter had evidence of antiretroviral resistance.

## Discussion

In this open-label demonstration project providing integrated delivery of ART and PrEP for HIV-1 prevention in couples, we observed high uptake of and adherence to both PrEP and ART as well as near-elimination of incident HIV-1 infection, with an HIV-1 incidence of <0.5% per year, compared to an expected incidence in this population of >5% per year. Our implementation approach differed substantially from randomized trials of ART and PrEP for HIV-1 prevention in its pragmatic strategy, with less frequent follow-up, counseling equivalent to what would be expected in public health settings, and its focused recruitment of couples with characteristics predicting elevated HIV-1 transmission risk. To our knowledge, this study is one of the first and one of the largest demonstration projects to provide PrEP to a priority population at risk for HIV-1 outside of a clinical trial setting, and the findings demonstrate the feasibility and impact of using PrEP as a bridging strategy to sustained HIV-1 protection by ART in HIV-1-serodiscordant couples.

Both ART and PrEP have been proven to be highly efficacious for preventing HIV-1 transmission within HIV-1-serodiscordant relationships [1,2]. Indeed, data from one clinical trial and several observational studies suggest that suppressive ART may provide complete protection against HIV-1 transmission [13]. However, delays in ART initiation are common for HIV-1 infected persons, particularly at higher CD4 counts, when infected persons may be largely without symptoms of HIV-1 disease [10]. Even in the HPTN 052 clinical trial, which recruited couples motivated to initiate ART and demonstrated ART’s protective effect for preventing HIV-1 transmission, almost 20% of HIV-1-infected partners who had been randomly assigned to delay ART until developing progressive HIV-1 disease declined to initiate for a year after the trial’s results were announced and ART was made available to all couples [14]. In addition, as demonstrated in HPTN 052 and other studies, HIV-1 transmission risk persists during the first months after ART is started, likely as a result of ongoing viral replication prior to the time when viral suppression is usually achieved. Sustained adherence is also critical to achieve and maintain the prevention benefits of ART. Thus, PrEP may offer HIV-1 protection to HIV-1-uninfected partners during the period prior to ART initiation and overlapping the period when viral concentrations are declining as a result of ART; this was the rationale for the time-limited use of overlapping PrEP and ART as implemented in the present study. ART uptake in our study was high, which is notable since HIV-1-infected partners were largely asymptomatic and many had high CD4 counts; this may suggest that an integrated approach to delivery of PrEP and ART within a coupled relationship could motivate ART initiation, a hypothesis that is supported by qualitative work from this setting [15]. Like for ART, PrEP requires uptake and sustained adherence to provide HIV-1 protection.

Because of the integrated delivery approach for ART and PrEP in the present study, we are unable to precisely define the relative contributions of each intervention; however, PrEP was used for three-quarters of the follow-up time, mostly without concurrent ART, indicating a substantial PrEP contribution to our findings. Two individuals, both women, acquired HIV-1 in spite of access to PrEP but with self-reported and objective evidence of interrupted PrEP use. Although clinical trials have demonstrated definitively that PrEP is efficacious for HIV-1 prevention for women, strategies to support adherence and continuously evaluate personal risk, particularly during relationship challenges and at the onset of new relationships, are needed.

Implementation models for PrEP delivery are being evaluated, as this prevention strategy has not been delivered widely to date. Our approach, with time-limited use of PrEP during a period with continuing HIV risk within a coupled relationship, was supported by prior mathematical modeling analyses predicting the prevention effectiveness and cost-effectiveness of the approach [8]. Cost considerations are important for antiretroviral-based HIV-1 prevention strategies, both early ART and PrEP, given flat global resources for HIV-1 prevention and treatment, and pragmatic delivery models will be needed for public health scale-up of ART and PrEP for HIV-1 prevention. For PrEP, cost-effectiveness in multiple analyses across populations has been dependent on targeting delivery to those individuals at greatest HIV-1 risk and limiting PrEP use to their highest-risk periods [8,16]. We used an empiric, validated risk scoring tool to recruit couples to the present study, demonstrating that this higher-risk subgroup, which had a predicted HIV-1 incidence comparable to the highest incidence populations observed in recent HIV-1 prevention studies [17,18], could use PrEP with high adherence. Cost analyses from the present study suggest that integrated PrEP and ART can be affordably delivered [8,9]. Other analyses from this cohort, including detailed behavioral and social science and more intensive assessment of the safety of PrEP use through pregnancy, will be reported separately.

Notably, PrEP adherence and HIV-1 prevention effectiveness were higher in this open-label demonstration project than in our previous clinical trial of PrEP among couples. Similar results with effectiveness exceeding efficacy has been seen in other open-label PrEP studies, which has been hypothesized to be a result of offering a strategy with demonstrated safety and efficacy, as compared to clinical trials, which tested an unproven product with a placebo comparison [19]. In addition, recruitment of individuals who recognize their elevated HIV-1 risk and are motivated to use PrEP may also contribute to high adherence in demonstration projects to date [20]. PrEP and ART use could be different in populations with lesser transmission risk or longer duration of knowledge of HIV-1 status. In addition, approximately 6% of couples who were eligible for this study did not enroll, which could reflect relationship instability, lack of interest in PrEP and/or ART and, thus, need for other prevention options, or unwillingness to participate in a study.

We used a counterfactual, simulation model to compare observed HIV-1 risk in this study to anticipated HIV-1 incidence in the absence of PrEP and ART provided at any CD4 count. This comparison was consistent with the implementation science approach of the project, and other potential comparisons—such as use of placebo, enrollment of a contemporary comparison population, or randomized assignment to delayed provision of ART and/or PrEP—would have had important ethical challenges. The use of a non-concurrent comparison is a potential limitation of our approach; however, secular changes in HIV-1 transmission risk within larger populations (such as all of Kenya and Uganda) would not be expected to substantially alter HIV-1 risk in known HIV-1-serodiscordant couples, where the principal risk derives from within the relationship. The empiric risk scoring tool we used to recruit couples for this study and select the counterfactual population was developed and validated in three separate cohorts of HIV-1-serodiscordant couples, from seven African countries and occurring over a decade, suggesting generalizability and stability over time [11]. Indeed, the population recruited for this project could be argued to be at higher risk than the comparison population, as the comparison group for the counterfactual models was a more carefully selected clinical trial population and received monthly risk-reduction counseling [2]. Twelve individuals (1.1% of the study population) in the present study were found to have been infected at enrollment, compared to 14 (of 4,747, 0.3%) in our prior PrEP clinical trial, suggesting substantial force of infection in the population recruited for this demonstration project. Finally, pregnancy incidence of approximately 20% per year in the present study population suggests HIV-1 exposure was ongoing.

New guidelines from the World Health Organization recommend ART for all persons with HIV-1 infection and PrEP as an additional prevention option for all persons at substantial HIV-1 risk [21]. Countries, including Kenya, where this study was in part conducted [22], have drafted national policies that seek to optimally use both ART and PrEP to reduce new HIV-1 infections in at-risk populations, including HIV-1 serodiscordant couples. The results of this project demonstrate that an integrated strategy of ART and PrEP can be delivered feasibly to a high-risk African population and result in almost complete protection from HIV-1 transmission. HIV-1 incidence in this study was lower than observed in both ART and PrEP clinical trials in couples [1,2], in spite of targeted recruitment for a subpopulation with substantially greater risk characteristics. Thus, this time-limited offering of PrEP bridging to ART demonstrates important synergies between ART and PrEP. Scale-up of these strategies is imperative in order to achieve the global goal to eliminate new HIV-1 infections and reverse the 30-year HIV-1 epidemic.

## Supporting Information

S1 Analysis Plan Statistical analysis plan. The analysis plan for the present assessment of HIV-1 incidence is included as supplementary text.
(PDF)

S1 Protocol Study protocol. The final study protocol is included as supplementary text.
(PDF)

S1 STROBE Checklist STROBE checklist. The STROBE checklist is included as supplementary text.
(DOCX)

S1 Table Serious adverse events. Data on serious adverse events and events felt by the treating clinician to be related to PrEP were collected.
(DOCX)

## Abbreviations

ART: antiretroviral treatment
LC-MS/MS: liquid chromatographic-tandem mass spectrometric
MEMS: medication electronic monitoring system
PrEP: pre-exposure prophylaxis
