# Supplementary material for: Integrated Delivery of Antiretroviral Treatment and Pre-exposure Prophylaxis to HIV-1–Serodiscordant Couples: A Prospective Implementation Study in Kenya and Uganda
Source: PLoS Med. 2016 Aug 23;13(8):e1002099. doi: 10.1371/journal.pmed.1002099 (PMC4995047; doi:10.1371/journal.pmed.1002099)
Supplement: S1 Analysis Plan — The analysis plan for the present assessment of HIV-1 incidence is included as supplementary text. (PDF) [file pmed.1002099.s001.pdf]

**STATISTICAL ANALYSIS PLAN**

# An open-label, pilot demonstration and evaluation project of antiretroviral-based HIV-1 prevention among high-risk HIV-1 serodiscordant African couples

***The Partners Demonstration Project***

Analysis Plan Version 0.1

30 January 2013


Deborah Donnell, PhD

Renee Heffron, PhD

Jared Baeten, MD, PhD



*Funding:*

*United States National Institutes of Health*

*Bill & Melinda Gates Foundation*

*United States Agency for International Development*

## Description of the Partners Demonstration Project

The Partners Demonstration Project is a prospective, open-label study evaluating uptake of, adherence to, and user preferences regarding pre-exposure prophylaxis (PrEP) and antiretroviral treatment (ART) for HIV-1 prevention among Kenyan and Ugandan HIV-1 serodiscordant couples. The overall goal is to develop sustainable models for delivery of antiretroviral-based HIV-1 prevention services. Critical unanswered questions for successful implementation of antiretroviral-based HIV-1 prevention include how to target these strategies to the highest-risk couples and whether HIV-1 infected persons with asymptomatic disease would accept ART to reduce their risk for transmitting HIV-1, at-risk HIV-1 negative persons would use PrEP, and both would sustain high adherence needed for high effectiveness.

Approximately 1000 HIV-1 serodiscordant couples will be enrolled in the Partners Demonstration Project at four clinical research sites. HIV-1 uninfected partners will be offered PrEP (daily oral emtricitabine/tenofovir, Truvada®). HIV-1 infected partners will be offered ART following national ART initiation guidelines. Together, this PrEP and ART strategy is best described as PrEP as a "bridge" to ART initiation and viral suppression in the partnership: when the HIV-1 infected partner is not yet taking ART, PrEP will be offered, and if the HIV-1 infected partner initiates ART, PrEP will be discontinued for the HIV-1 uninfected partner six months later (i.e., once viral suppression in the HIV-1 infected partner would be expected to be typically achieved) (Figure 1).

**Figure 1. Study approach**

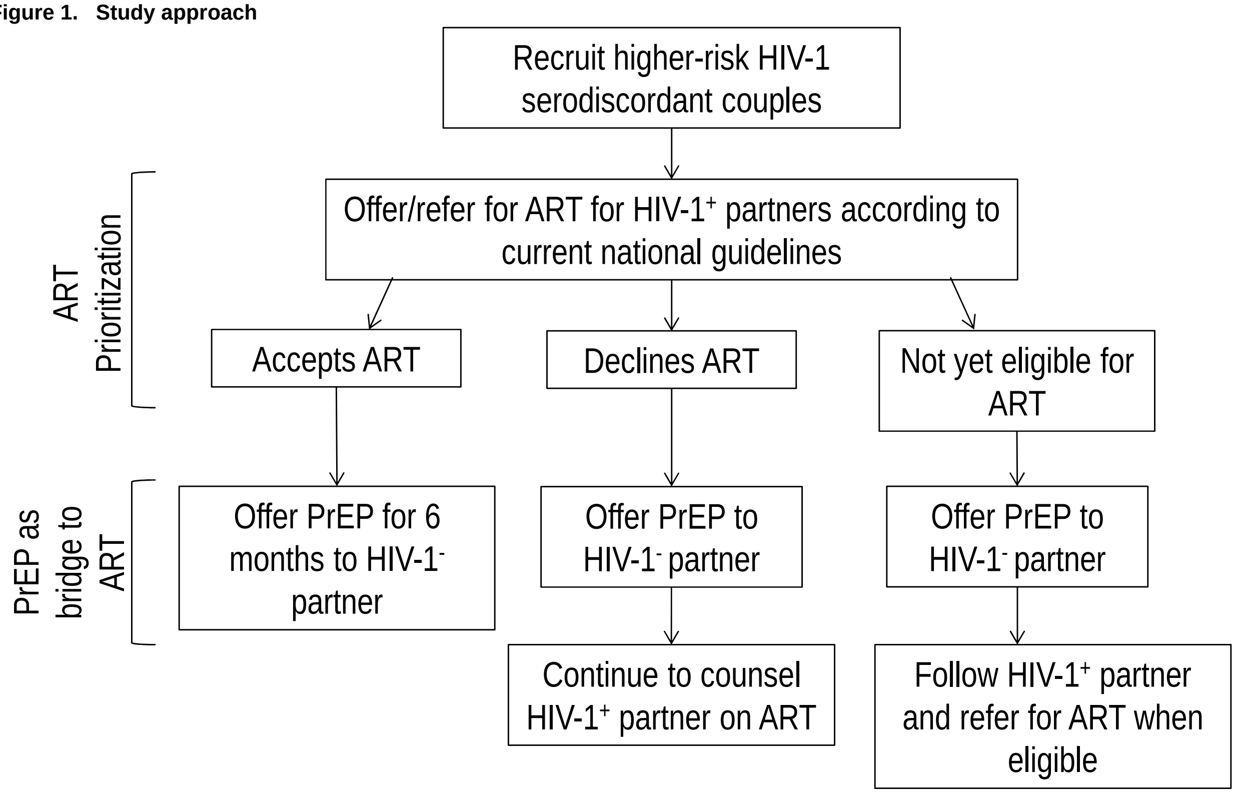


The Partners Demonstration Project is designed to be representative of "real world" implementation of PrEP, with less stringent eligibility criteria and less frequent clinic visits (quarterly) than in the clinical trials of PrEP and ART for HIV-1 prevention. The target population

of the Partners Demonstration Project is men and women with a known HIV-1 infected stable partner who are at increased HIV-1 risk, with recruitment based on a standardized risk score metric (Kahle et al. *JAIDS* in press) which will select for those with an anticipated HIV-1 incidence of >4% per year. The risk score was developed from data from prior prospective studies among HIV-1 serodiscordant couples – higher scores predicted greater HIV-1 incidence.

This document presents the Statistical Analysis Plan for the Partners Demonstration Project. As an observational study with substantial data collection, it is anticipated that all aspects of analyses to be done for Partners Demonstration Project cannot be anticipated in advance of the project. Thus, the goal of this Statistical Analysis Plan is to address core issues, including operational management, overarching goals of the project related to HIV-1 prevention and delivery of antiretroviral-based HIV-1 prevention, and specific outcomes of the work, mapped to each of the study Aims.

**Operational management**

A number of reports will be run periodically to track participant screening/eligibility, enrollment, and retention (e.g., Tables 1 and 2). Additional reports will track pregnancy incidence and contraceptive use, participant demographics, and other operational metrics. Finally, we will closely track PrEP and ART initiation, continued use, and discontinuation. A flow chart will depict how couples flow through PrEP and ART use from enrollment through follow-up (Figure 1).

**Table 1: Enrollment report (to be run weekly)**

| | | Number of couples enrolled during month of: | | | | | | | | | |
|---|---|---|---|---|---|---|---|---|---|---|---|
| Site | Date of first enrollment | Enrolled prior | 11-2012 | 12-2012 | 1-2013 | Average monthly enrollment, past 3 months | Total couples enrolled | Screen: Enroll ratio | % HIV- beginning PrEP at enrollment | % HIV+ eligible for ART | % ART eligible accepting ART at enrollment |
| Kabwohe | | | | | | | | | | | |
| Kampala | | | | | | | | | | | |
| Kabwohe | | | | | | | | | | | |
| Thika | | | | | | | | | | | |

**Table 2: Retention report (to be run monthly, separately for HIV-1 infected and uninfected partners)**

| Site | | M1 | M3 | M6 | M9 | M12 | M15 | M18 | M21 | M24 |
|---|---|---|---|---|---|---|---|---|---|---|
| Kabwohe | N Expected | | | | | | | | | |
| | % Completed | | | | | | | | | |
| Kampala | N Expected | | | | | | | | | |
| | % Completed | | | | | | | | | |
| Kabwohe | N Expected | | | | | | | | | |
| | % Completed | | | | | | | | | |
| Thika | N Expected | | | | | | | | | |
| | % Completed | | | | | | | | | |

**Figure 1. PrEP and ART use at enrollment and during follow up**

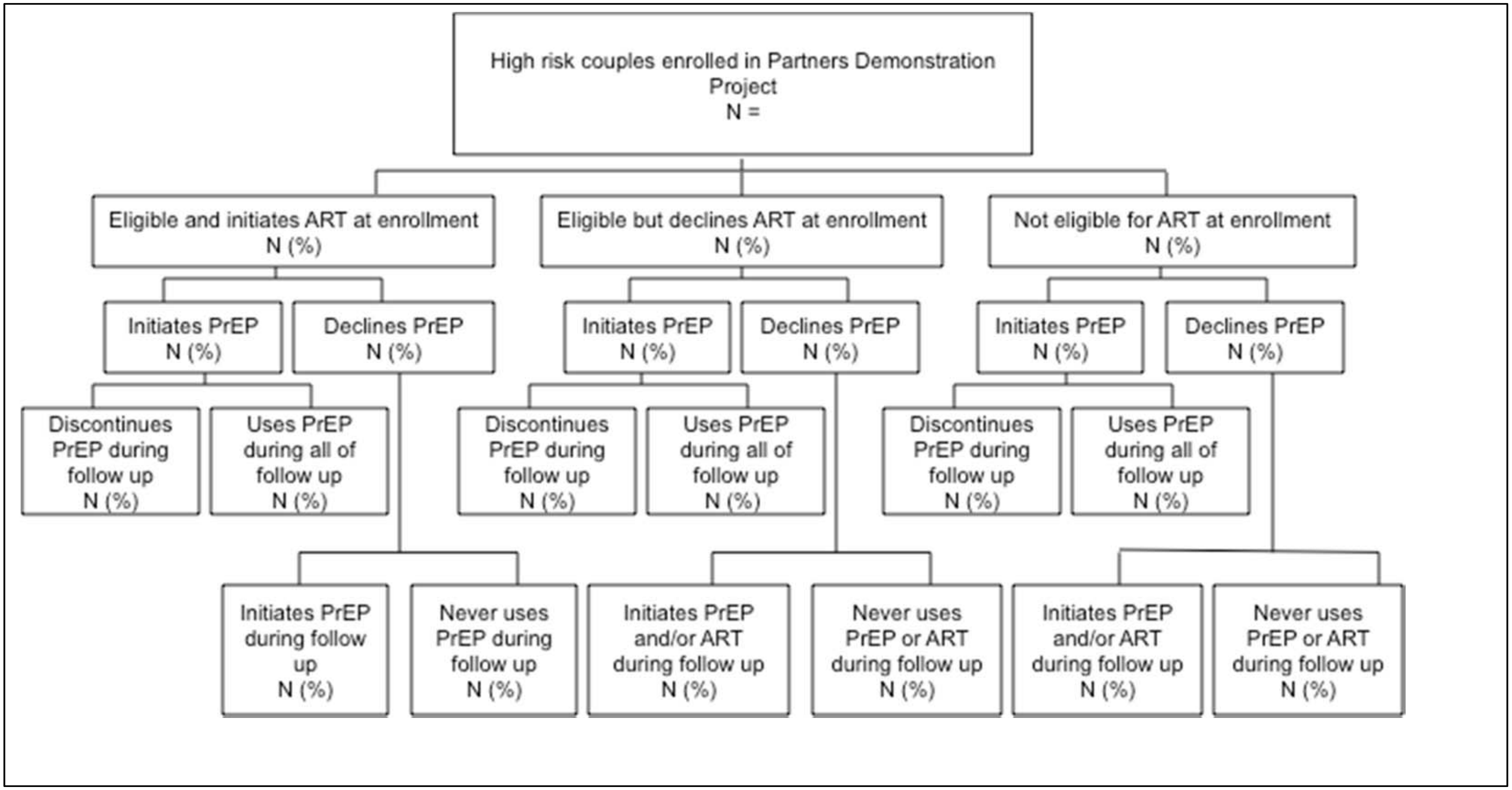

**Overarching Goal**

This goal is cross-cutting, across protocol aims, and is thus presented overall.

**We will evaluate the effectiveness of the Partners Demonstration Project combination prevention intervention in reducing HIV-1 infection rates in HIV-1 serodiscordant couples**

> *Using historical data from the placebo arm of the Partners PrEP Study (a prior prospective study among HIV-1 serodiscordant couples) and the same distribution of risk scores (≥5), we will compare the incidence of HIV-1 infection in the Partners Demonstration Project to the counterfactual HIV-1 incidence estimated in the Partner's PrEP placebo arm for the same distribution of risk scores.*

Statistical methods:

Comparisons between the Partners Demonstration Project and the similar risk score distribution group within the Partners PrEP Study will use scenarios described below.

a) HIV-1 incidence in the full Partners Demonstration Project cohort will be compared to data from the Partners PrEP Study cohort with the same risk score distribution
b) HIV-1 incidence in the full Partners Demonstration Project cohort will be compared to data from Partners PrEP Study participants cohort with the same risk score distribution enrolled at the same project sites as those that are participating in the Partners Demonstration Project

Non-parametric bootstrap resampling methods will be used to construct pseudo-populations from the Partners PrEP study with the same distribution of risk scores as the Partners Demonstration Project cohort. HIV-1 infection rates and confidence intervals will be computed for each study. Formal inference for difference in HIV-1 incidence between cohorts will acknowledge that temporal trends and differences between study cohorts in addition to the Demonstration Project intervention could contribute to observed differences in incidence.

**Aim 1: We will evaluate the ability to do targeted enrollment of high-risk HIV-1 serodiscordant couples into a longitudinal HIV-1 prevention study**

*The primary outcomes of this aim will be the screened to eligible ratio of HIV-1 serodiscordant couples recruited for this open-label study, the proportion of eligible couples who decide to enroll in the cohort, and the costs of screening and targeting high-risk couples.*

Statistical methods:

a) Descriptive: among ineligible, ineligible reasons will be categorized as due to low risk score; ART use; etc.
b) Descriptive: among those declining enrollment, reasons will be categorized as related to PrEP use, ART use or other.
c) Descriptive: baseline risk profile of eligible couples will include risk score, age of the HIV-1 uninfected partner, married and/or cohabiting partnership, number of children, unprotected sex, uncircumcised male HIV-1 uninfected partner, and HIV-1 plasma viral load.
d) Comparison: The risk profile of enrolled and eligible but not enrolled couples will be compared. (e.g., Table 3: Risk characteristics)

A flow diagram will be used to depict screened and enrolled participants (Figure 2)

Throughout this statistical analysis plan, descriptive presentation of categorical data will display both n/N and %. Comparisons, when appropriate, will use chi-square tests for baseline characteristics; and logistic regression for adjusted comparisons. Descriptive presentation of continuous variables will be described with means and standard errors or medians and upper and lower quartiles. Comparison will use the t-test or Mann-Whitney test for baseline comparisons, and linear regression for adjusted comparisons. No stratification or adjustment for study site is planned.

Additional analyses to evaluate costs will be performed.

**Table 3: Characteristics of eligible couples (by enrollment status)**

| | Enrolled<br>N= | Not enrolled<br>N= |
|---|---|---|
| | N (%) or Median (IQR) | |
| **Demographics** | | |
| Age of HIV-1 uninfected partner, years | | |
| Age of HIV-1 infected partner, years | | |
| Number of children in partnership | | |
| Study partners are married to each other | | |
| Partnership duration | | |
| Years aware of HIV-1 serodiscordancy | | |
| **Sexual behavior & Medical characteristics** | | |
| Unprotected sex with study partner, month prior | | |
| CD4 count (HIV-1 infected partners) | | |
| HIV Plasma viral load (HIV-1 infected partners) | | |
| Circumcision status (male HIV-1 uninfected partners) | | |
| Risk Score<br>5<br>6<br>7 or more | | |

**Figure 2: Example flow chart showing screened and enrolled participants**

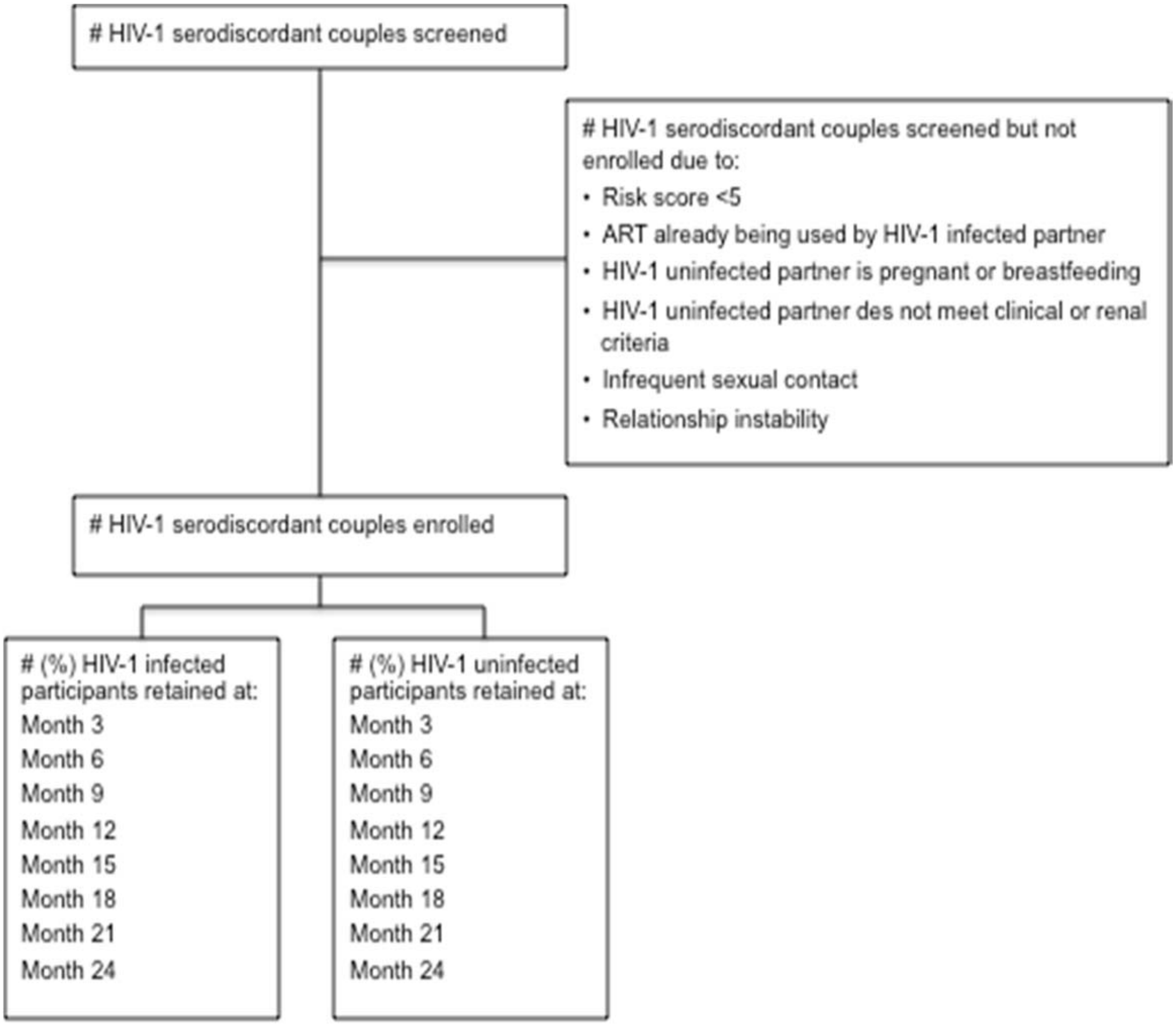

**Aim 2:** **We will assess user preferences among high-risk HIV-1 serodiscordant couples for ART initiation for HIV-1 infected partners and PrEP for HIV-1 uninfected partners.**

*The primary outcomes for this aim will be measured at baseline and quarterly through interviewer-administered questionnaires. The questionnaires will specifically ask about willingness to use ART for HIV-1 prevention and PrEP use and which method the participant would prefer (ART or PrEP). Reasons for the choice and concerns about both methods will also be collected through mixed-methods approaches.*

Statistical methods: McNemars test will be used to assess agreement between preference and uptake.

Baseline preferences will be described and compared by
a) Gender
b) HIV-1 infection status
c) CD4 count and symptom status of the HIV-1 infected partner

Follow-up preferences will tabulated by agreement with
a) Actual current PrEP use (Y/N) in HIV-1 uninfected partner
b) Actual current ART use in HIV-1 infected partner and
c) Actual current couple uptake of ART (Y/N) and PrEP (Y/N)

Qualitative data analysis: Translated transcripts will be reviewed separately by at least two analysts for text element and key word coding and initial theme generation. We will use focused coding in a content analysis approach. Coders will meet to review categories and agree on a matrix that can be applied to interviews.

**Aim 3:** **We will ascertain initiation of and adherence to PrEP among HIV-1 uninfected partners, as a bridge to ART.**

*The primary measures of adherence will be collection of PrEP, clinic-based pill counts of unused study pills as recorded at each follow-up visit, and MEMS cap data on how frequently the pill bottle was opened. Blood samples for detection and quantification of PrEP levels (testing in batch) will be done for those who seroconvert to HIV-1 and a subset who remain HIV-1 uninfected.*

We will use 3 measures to identify PrEP use:
a) Proportion of recruited couples who initiate PrEP
   - At enrollment
   - At any time during the study
   - After enrollment but later in the study
b) The primary measures of PrEP adherence will be
   - Dispensing of PrEP: how many PrEP bottles are dispensed / expected maximal number. (pharmacy refill data)
   - Clinic-based pill counts of dispensed study pills as recorded at each follow-up visit. (pill count adherence of dispensed product)
   - MEMS cap. (objective measure of daily use)
c) Blood samples for detection and quantification of PrEP levels (testing in batch) will be tested for those who seroconvert to HIV-1 and a subset of those who do not acquire

HIV-1.

Statistical methods:

a) Initiation of PrEP: Simple descriptive proportions of participants initiating PrEP will be supplemented with rates that account for time retained in study follow-up using survival analysis methods.
b) Adherence:
    - Dispensing of PrEP will be described in the cohort initiating PrEP, accounting for periods of time when PrEP is withheld or stopped (e.g., due to clinical hold reasons or after 6 months of ART use by the HIV-1 infected partner)
    - Pill count adherence will be described for periods following visits where PrEP is dispensed until the bottle is returned as number of pills not returned / number of days elapsed. Bottles that are not returned will be reported separately.
    - MEMS cap adherence will be described for the period when a MEMS cap is being used as how many days in the period the bottle was opened. This will account for any extra openings or times when multiple pills are taken out with one opening, as reported during participant study visits.
c) Detection of PrEP at seroconversion. A case-control analysis will assess the relative risk of seroconversion for detectable levels of PrEP.

**Aim 4:** **We will ascertain initiation of and adherence to ART among HIV-1 infected partners.**

*The primary measures of ART adherence will be ART initiation at a local ART provider and plasma HIV-1 RNA levels taken every 6 months for HIV-1 infected partners.*

Measures of ART adherence will include:

a) ART initiation at a local ART provider:
   - ART initiation (not including short-course therapy for PMTCT) by quarter amongst those referred for initiation, according to national guidelines for initiation

b) Plasma HIV-1 RNA levels taken every 6 months for HIV-1 infected partners.
   - Time to first undetectable plasma RNA
   - Proportion with undetectable plasma RNA as measured at the 6, 12, 18, and 24 month follow-up visits.

c) Time to treatment failure (first detectable RNA after achieving viral suppression)

Statistical Methods:
As ART for treatment is not dispensed by the study staff, analysis methods will appropriately account for delay in reporting information into the study database.

a) ART initiation: Initiation time will be assessed only in the cohort with a visit after ART referral. Survival analysis methods (e.g., Kaplan-Meier curves) will be used to estimate the probability of ART initiation after becoming eligible (and referred by study staff).
b) Viral suppression and treatment failure: The proportion ever achieving undetectable viral load and ever experiencing treatment failure after viral suppression is achieved will be reported. Viral suppression will be assessed relative to the date ART is initiated. Methods that account for interval censoring will be used to estimate the proportion with undetectable plasma RNA at 6, 12, 18, and 24 months after ART initiation. Time to treatment failure will be assessed relative to the first occurrence of undetectable viral load. Only those achieving viral failure and with a subsequent visit will be included in this assessment.
c) Care cascade: The number and proportion at each step of the care cascade will be described: i.e. number eligible for ART, contacted, offered, accepted, started and virally suppressed.

**Aim 5:** **We will assess factors influencing preferences, uptake and adherence for antiretroviral-based HIV-1 prevention**

*Through interviewer-administered questionnaires, correlates of preferences, uptake and adherence to ART and PrEP will be assessed, including characteristics of sexual risk, psycho-social factors and clinical characteristics of the HIV-1 infected partner.*

Outcomes for PrEP:

a) Uptake: Initiated PrEP at baseline
b) Adherence: Remains on PrEP at visit. Cohort of those who initiated PrEP during the time they are meant to be on PrEP

Outcomes for ART:

a) Uptake: Initiated ART (Both overall, and amongst referred for ART )
b) Adherence: Virally suppressed at visits after ART initiation

Covariates

a) Gender
b) Any unprotected sex
c) Coital frequency
d) Any outside partners by HIV-uninfected partner
e) Depression scale (as measured by the Hopkins Symptoms Checklist)
f) Alcohol use scale (as measured by the RAPS4)
g) Additional sociobehavioral measures
h) Living together (baseline); Still together as couple (follow-up)
i) Fertility intentions
j) Initiated ART/achieved viral suppression (PrEP only)
k) Initiated PrEP (ART only)
l) CD4 of HIV-1 infected partner
m) WHO stage

Statistical methods:

a) Initiation of PrEP: Logistic regression will be used to explore the relationship between PrEP initiation and baseline covariates.
b) Adherence to PrEP: GEE models will be used to assess association of baseline and time-varying covariates with remaining on PrEP amongst those who initiated PrEP at baseline.
c) Initiation of ART: Logistic regression will be used to explore the relationship between ART initiation and covariates during the period of initiation. To account for reporting lag only those with a visit following referral will be included. Covariate assessment will encompass values at referral and prior to and after initiation.
d) Adherence to ART: GEE models will be used to assess the association of baseline and time-varying covariates with viral load suppression at visits more than 6 months after ART initiation.

During modeling, each covariate will be assessed in a univariate relationship, and then included in the multivariate model with those deemed scientifically plausible and/or statistically reliable.

**Aim 6: We will assess the feasibility of PrEP discontinuation in couples in which the HIV-1 infected partner initiates ART**

Through interviewer-administered questionnaires, we will assess couples' attitudes and understanding of PrEP discontinuation.

Outcome:

a) Proportion of HIV-1 uninfected partners discontinuing PrEP 6 months after ART initiation by their HIV-1 infected partner.
b) Because of clinician discretion, reasons for non-discontinuation of PrEP after 6 months of ART use by the HIV-1 infected partner.
c) Measures of attitudes and understanding of PrEP discontinuation.

Covariates:

a) ART initiation and viral suppression in HIV-1 infected partners
b) Adherence to ART (by self-report)
c) Outside partners
d) Continuation of partnership/partnership dissolution
e) Fertility intentions
f) HIV-1 risk perception

Statistical Methods:
PrEP discontinuation will be assessed among couples who initiated both PrEP and ART. The number and proportion of these couples who decide to discontinue PrEP will be described. Reasons for not discontinuing PrEP will be presented, including HIV-1 infected partner having a detectable viral load, outside partners, and immediate fertility intentions. If statistically reliable, logistic regression will be used to explore correlates of PrEP discontinuation. Attitudes and understanding will be described for those who discontinue PrEP after 6 months of ART use by the HIV-1 infected partner within a couple.

**Aim 7: We will assess PrEP use and birth outcomes among HIV-1 uninfected women who choose to continue PrEP during pregnancy**

*HIV-1 uninfected women who become pregnant will be given the opportunity to participate in enhanced follow up during pregnancy - regardless of their choice to continue or discontinue PrEP. We will monitor for HIV-1 seroconversion, serious adverse events, congenital malformations, and infant growth.*

Outcomes:

a) HIV-1 infection
b) Maternal elevated creatinine levels during pregnancy
c) Congenital anomalies in infants
d) Growth: Head circumference, weight, height

Statistical Methods: Methods will be primarily descriptive as the number of pregnancies is expected to be modest.

a) HIV-1 infection rates will be compared in four groups: women not pregnant who are 1) using and 2) not using PrEP and women while pregnant who are 3) using and 4) not using PrEP.
b) SAEs will be reported for all pregnant women, separately for periods using and not using PrEP
c) Growth parameters for infants will be compared to normalized WHO growth curves at birth and 1 year of age. Z-scores will be calculated and will be compared to those from infants from the Partners PrEP Study placebo arm (historical controls, without PrEP exposure).
