# Supplementary material for: Integrated Delivery of Antiretroviral Treatment and Pre-exposure Prophylaxis to HIV-1–Serodiscordant Couples: A Prospective Implementation Study in Kenya and Uganda
Source: PLoS Med. 2016 Aug 23;13(8):e1002099. doi: 10.1371/journal.pmed.1002099 (PMC4995047; doi:10.1371/journal.pmed.1002099)
Supplement: S1 Protocol — The final study protocol is included as supplementary text. (PDF) [file pmed.1002099.s002.pdf]

PROTOCOL

An open-label, pilot demonstration and evaluation project of antiretroviral-based HIV-1 prevention among high-risk HIV-1 serodiscordant African couples

The Partners Demonstration Project

Version 3.0

10 December 2012

Funding:

United States National Institutes of Health

Bill & Melinda Gates Foundation

United States Agency for International Development

STUDY TEAM

Coordinating Center - University of Washington, Seattle, USA

Jared Baeten, MD, PhD
Connie Celum, MD, MPH
Deborah Donnell, PhD
Renee Heffron, PhD
Andrew Mujugira, MBChB, MSc
Bettina Shell-Duncan, PhD
Ruanne Barnabas, MBChB, DPhil

Thika, Kenya site - Kenyatta National Hospital, Nairobi, Kenya

Nelly Mugo, MBChB, MMed, MPH
Kenneth Ngure, MPH, PhD

Kampala, Uganda site - Makerere University, Kampala, Uganda

Elly Katabira, MBChB
Edith Nakku-Joloba, MBChB, PhD
Nulu Bulya, MBChB, MPH

Kisumu, Kenya site - Kenya Medical Research Institute, Nairobi, Kenya

Elizabeth Bukusi, MBChB, MMed, MPH, PhD
Josephine Odoyo, KRCHN, MPH

Kabwohe, Uganda site - Kabwohe Clinical Research Centre, Kabwohe, Uganda

Elioda Tumwesigye, MBChB, MS
Stephen Asiimwe, MBChB, MS
Rogers Twesigye, MBChB, MPH

Additional sites pending funding

Adherence – Harvard University, Boston, USA

David Bangsberg, MD, MPH
Jessica Haberer, MD, MPH
Norma Ware, PhD

I. SUMMARY

Antiretroviral-based HIV-1 prevention strategies – including 1) antiretroviral treatment (ART) to reduce the infectiousness of HIV-1 infected persons and 2) pre-exposure prophylaxis (PrEP) for uninfected persons to prevent HIV-1 acquisition – are among the most promising new approaches for dramatically decreasing HIV-1 spread. A priority population for implementation of ART and PrEP for HIV-1 prevention is HIV-1 serodiscordant couples (i.e., one member is HIV-1 infected and the other uninfected). Stable, heterosexual African HIV-1 serodiscordant couples face high risk of HIV-1 transmission, from within the partnership and from outside partners, and are a priority population for prevention interventions. Importantly, during the past year, both ART (HPTN 052) and PrEP (Partners PrEP Study) have demonstrated high efficacy for HIV-1 protection when used by members of HIV-1 serodiscordant couples. Critical unanswered questions for successful implementation of antiretroviral-based HIV-1 prevention include how to target these strategies to the highest-risk couples and whether HIV-1 infected persons with asymptomatic disease would accept ART to reduce their risk for transmitting HIV-1, at-risk HIV-1 negative persons would use PrEP, and both would sustain high adherence needed for high effectiveness. This protocol describes an open-label prospective study among high-risk African HIV-1 serodiscordant couples to determine user preferences for ART and PrEP and to optimize targeted delivery and sustained use of these interventions.

Design: Prospective, observational, open-label cohort study
Up to 24 months of follow-up per couple

Population: Heterosexual HIV-1 serodiscordant couples in Kenya and Uganda who did not participate in the Partners PrEP Study (approximately 1000 couples in total)

Study Sites: Thika, Kenya; Kampala, Uganda; Kisumu, Kenya; and Kabwohe, Uganda Partners PrEP Study sites
Additional sites will be added if funding permits.

Approach: We will enroll couples with characteristics defining higher HIV-1 transmission risk (i.e., to target those for ART/PrEP that are at greatest risk for HIV-1, a public health priority). We will provide or refer HIV-1 infected participants to ART services following national ART initiation guidelines, recognizing that guidelines for ART initiation (including potentially recommending ART for all HIV-1 infected members in serodiscordant relationships) may change during the course of the study. PrEP will be offered to the HIV-1 uninfected partners as a “bridge” to ART initiation and viral suppression in the partnership; uptake and adherence to PrEP will be measured. **Specifically, PrEP as a bridge to ART will be done as follows: when the HIV-1 infected partner is not yet taking ART, PrEP will be offered, and if the HIV-1 infected partner initiates ART, PrEP will be discontinued for the HIV-1 uninfected partner six months later (i.e., once viral suppression in the HIV-1 infected partner would be expected to be typically achieved).** We will conduct mixed-methods work to understand user preferences, couples decision-making, risk perception, fertility intentions, and barriers to uptake of and adherence to both PrEP and ART. This bridge to ART approach is detailed in Figure 1.

Figure 1. Study approach

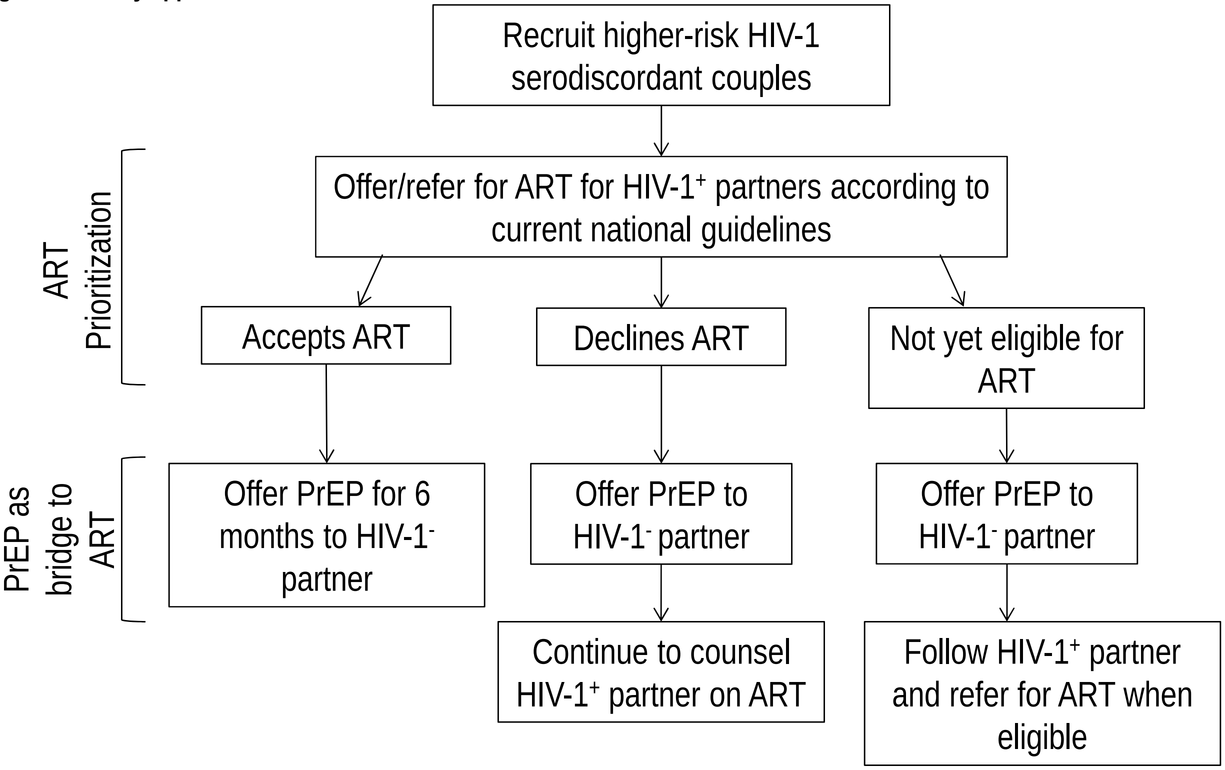

Specific Aims:

Aim 1: **We will evaluate the ability to do targeted enrollment of higher-risk HIV-1 serodiscordant couples into a longitudinal HIV-1 prevention study**

A critical aspect of implementation and cost-effectiveness of antiretroviral-based HIV-1 prevention in HIV-1 serodiscordant couples will be the ability to utilize behavioral, epidemiologic and clinical data to identify those couples who are at highest risk of HIV-1 acquisition. We have developed a risk score using enrollment data from our previous studies of >8500 HIV-1 serodiscordant couples that is able to discriminate couples at highest risk. The score is comprised of a limited set of variables that can be obtained in research and clinical settings: plasma HIV-1 levels in the HIV-1 infected partner, any unprotected sex in the couple in the prior month, number of children in the partnership, age of the HIV-1 uninfected partner, and circumcision status of HIV-1 uninfected male partners.

The primary outcomes of this aim will be the screened to eligible ratio of HIV-1 serodiscordant couples recruited for this open-label study, the proportion of eligible couples who decide to enroll in the cohort, and the costs of screening and targeting high-risk couples.

Aim 2: **We will assess user preferences among high-risk HIV-1 serodiscordant couples for ART initiation for HIV-1 infected partners and PrEP for HIV-1 uninfected partners.**

Understanding couples' preferences for and concerns about antiretroviral-based HIV-1 prevention is of utmost importance at this time when guidelines for use of PrEP and ART are being formulated.

The primary outcomes for this aim will be measured at baseline and quarterly through an interviewer-administered questionnaire. The questionnaire will specifically ask about willingness to use PrEP or ART for HIV-1 prevention and which method the participant would prefer. Reasons for the choice and concerns about both methods will also be collected through mixed-methods approaches.

Aim 3: We will ascertain initiation of and adherence to PrEP among HIV-1 uninfected partners, when implemented as a bridge to ART.

Adherence to PrEP is critical to its effectiveness. Understanding adherence to PrEP is a priority, particularly when PrEP is delivered outside of a controlled clinical trial setting. For this study, brief adherence counseling, to reflect real-world settings, will be delivered at baseline and then quarterly thereafter. The content of the adherence counseling will be derived from the counseling messages and procedures in the Partners PrEP Study, and informed by the findings from an ancillary adherence study conducted as part of the Partners PrEP Study. We will explore factors that are related to the decision to start and continue to use PrEP.

The primary measures of adherence will be collection of PrEP, clinic-based pill counts of unused study pills as recorded at each follow-up visit, and MEMS cap data on how frequently the pill bottle was opened. Blood samples for detection and quantification of PrEP levels (testing in batch) will be done for those who seroconvert to HIV-1 and a subset who remain HIV-1 uninfected.

Aim 4: We will ascertain initiation of and adherence to ART among HIV-1 infected partners.

In addition to providing clinical benefit to HIV-1 infected partners, ART also confers HIV-1 prevention benefits to HIV-1 uninfected partners. However, a substantial proportion of HIV-1 infected persons who are eligible for ART decline or delay initiation, for a number of reasons. In this study, HIV-1 infected partners who become eligible for ART will be actively encouraged to initiate, including counseling to improve their knowledge and understanding of ART and the benefits for themselves and their partner and the importance of strict adherence to ART regimens to achieve viral suppression. We will explore factors that are related to the decision to start and continue to use ART.

The primary measures of ART adherence will be ART initiation at a local ART provider and plasma HIV-1 RNA levels taken every 6 months for HIV-1 infected partners.

Aim 5: We will assess factors influencing preferences, uptake and adherence for antiretroviral-based HIV-1 prevention.

Understanding factors that influence couples' preferences and their likelihood to initiate and adherence to PrEP and/or ART in a real-world setting is critical for providers who will counsel HIV-1 serodiscordant couples and make

recommendations about the HIV-1 prevention practices that are best indicated for couples.

Through interviewer-administered questionnaires, correlates of preferences, uptake and adherence to ART and PrEP will be assessed, including characteristics of sexual risk (i.e., sexual frequency, condom use, whether the HIV-1 uninfected partner reports outside partners), depression and substance use, gender of the HIV-1 uninfected partner, fertility intentions, partnership characteristics (whether the couple is cohabiting, has children, is together throughout follow-up, whether members have other partners, and whether the HIV-1 infected partner initiates ART), and clinical characteristics of the HIV-1 infected partner (e.g., CD4 count, WHO stage).

Aim 6: We will assess the feasibility of PrEP discontinuation in couples in which the HIV-1 infected partner initiates ART

ART is life-long, but PrEP need not be. In fact, time-limited use of PrEP for highest-risk persons and periods of time is likely the most cost-effective approach – since ART has substantial prevention benefits, any additional benefit of PrEP would be small for limiting risk within the partnership. Moreover, time-limited use of PrEP – e.g., until the HIV-1 infected partner initiates ART and is on ART for 6 months (the time by which viral suppression is typically achieved) – would limit risk of long-term side effects or toxicity of a daily PrEP medication and potentially improve adherence, since use would be for a defined period of time. Thus, time-limited use of PrEP has the greatest chance of being implemented widely. In couples in which the HIV-1 infected partner initiates ART, we will discontinue PrEP six months later. It is possible that HIV-1 uninfected partners may be reluctant to discontinue PrEP, for example if they do not trust their partner's motivation to consistently use ART or if the uninfected partner has additional sexual partners. We will explore the ability to discontinue PrEP.

Through interviewer-administered questionnaires, we will assess couples' attitudes and understanding of PrEP discontinuation.

Aim 7: We will assess PrEP use and birth outcomes among HIV-1 uninfected women who choose to continue PrEP during pregnancy

Pregnancy represents a period of heightened HIV-1 risk for women, and for their infant if acute HIV-1 infection occurs. Preventing HIV-1 acquisition in pregnant women is a priority. Data from PrEP clinical trials and from clinical experience with FTC/TDF as part of combination antiretroviral therapy for HIV-1 infected women have shown that FTC/TDF does not appear to increase the risk of major birth defects. A priority for high-risk HIV-1 uninfected women is to assess PrEP use during pregnancy.

HIV-1 uninfected women who become pregnant will consented to monthly follow-up during pregnancy - regardless of their choice to continue or discontinue PrEP. We will monitor for HIV-1 seroconversion, serious adverse events, congenital malformations, and infant growth.

II. BACKGROUND & RATIONALE

More than 2.5 million persons are infected with HIV-1 each year, the majority in sub-Saharan Africa, the region with the highest prevalence [2]. After 30 years of the HIV-1 epidemic, novel, effective HIV-1 prevention strategies remain urgently needed, particularly those that are deliverable to and useable by high-risk populations. To achieve population-level impact of strategies that are proven efficacious in controlled trials, effective delivery systems and high uptake are critical [3]; to lower the costs per infection averted, targeted delivery to those at highest HIV-1 risk will be essential.

The potential of antiretroviral-based HIV-1 prevention strategies to change the epidemic

Antiretrovirals markedly increase the survival of HIV-1 infected persons and are the cornerstone of strategies to prevent vertical HIV-1 transmission. During the past 10 years, a growing scientific and advocacy interest in antiretroviral-based strategies for prevention of sexual HIV-1 transmission has developed, and antiretroviral-based HIV-1 prevention interventions are now among the most promising strategies for dramatically reducing HIV-1 spread [4]. Antiretrovirals have the potential to be used for HIV-1 prevention as 1) antiretroviral treatment (ART) to reduce the infectiousness of HIV-1 infected persons and 2) oral or topical pre-exposure prophylaxis (PrEP) for uninfected persons with repeated and ongoing HIV-1 exposure [5, 6].

ART reduces HIV-1 transmission

The primary determinant of risk of HIV-1 transmission is the concentration of HIV-1 in plasma [7, 8] and genital secretions [9]. ART reduces HIV-1 plasma concentrations to undetectable levels within 6 months of initiation in the majority of persons [10, 11] and seminal and cervicovaginal HIV-1 concentrations are also reduced to undetectable levels in most persons on ART [12-16]. Use of peripartum ART is responsible for the remarkable success in virtually eliminating mother-to-child HIV-1 transmission in resource-rich settings [17].

It was hypothesized that the substantial reduction in HIV-1 quantity in plasma and genital compartments in persons on suppressive ART would translate into markedly reduced risk of HIV-1 transmission to sexual partners [18]. A meta-analysis of data from 11 cohorts among 5021 heterosexual HIV-1 serodiscordant couples with 1098 person-years follow-up, found only five cases of HIV-1 transmission to sexual partners from HIV-1 infected persons receiving ART, consistent with a transmission rate between 0.19 and 1.09 per 100 person-years [19].

Among 3381 HIV-1 serodiscordant couples in the Partners in Prevention HSV/HIV Transmission Study, we found a 92% reduction (95% CI 43-100%, p=0.004) in HIV-1 transmission risk among the 349 couples in which the HIV-1 infected partners initiated ART during follow-up (1 transmission event in 273 person-years of follow-up), compared to those who did not start ART (102 transmission events in 4558 person-years of follow-up, Table 1) [20]. As in the earlier meta-analysis [19], we found a very low rate of HIV-1 transmission (<0.5% per year) after ART initiation. The single HIV-1 transmission event observed after ART initiation occurred less than four months after ART was begun, and thus it is probable that transmission occurred prior to complete HIV-1 suppression as a result of ART.

In 2011, the observational data associating ART initiation with substantial reduction in HIV-1 risk were confirmed by HPTN 052, a randomized trial among 1763 HIV-1 serodiscordant couples [21]. At the time of enrollment into the trial, all HIV-1 infected partners had a CD4 count between 350 and 550 cells/mm^3 (thus not meeting international guidelines for ART provision). HIV-1 infected partners were randomly assigned to immediately initiate ART or refrain from ART until their CD4 count fell to ≤250 cells/mm^3, consistent with standard practice at the time. In

April 2011, the trial's independent Data and Safety Monitoring Board (DSMB) recommended public report of the study results because HIV-1 prevention benefits of earlier ART initiation had become clearly demonstrated (i.e., the statistical calculations for the study crossed a pre-specified efficacy threshold for early discontinuation of the delayed ART arm). Of 39 HIV-1 transmissions observed in the study, 28 were virologically-linked within the study partnership: 27 in the delayed ART arm and only 1 in the immediate ART arm, a 96% reduction in HIV-1 risk that was highly statistically significant (RR 0.04, 95% 0.01-0.27, p<0.001) [21]. The single transmission in the immediate ART arm was observed soon after ART initiation, a similar situation to what we had observed in the observational Partners in Prevention HSV/HIV Transmission Study analysis. Notably, HPTN 052 conducted quarterly viral load monitoring with intensive follow-up and adherence counseling for those on ART who were not achieving viral suppression, and as a result they achieved 89% of participants on ART with viral suppression within 3 months and 97% at 24 months [21].

Mathematical modeling by WHO has stimulated great interest in the potential of ART to substantially reduce population HIV-1 incidence when administered through near-universal annual HIV-1 testing, linkage to care, and uptake of ART, regardless of CD4 count (together called the 'Test and Treat' or 'Test and Linkage to Care' concept) [22]. Most individuals are infected for several years before CD4 decline or clinical disease necessitates ART, and although WHO HIV-1 treatment guidelines now recommend ART initiation at CD4 counts <350 cells/μL, <200-250 remains the standard in many countries, and the average CD4 at ART initiation is <100 in many settings, often due to late testing or fears about ART [23]. While ART adherence has been excellent in Africa [24], this success has been exclusively in individuals with advanced disease whose families are dedicated to provide tangible support to overcome severe structural and economic barriers to adherence because of the dramatic functional improvement they witness with ART. It is unclear whether asymptomatic individuals and their families will share the same commitment to adherence when ART is given to asymptomatic individuals.

Recent evidence demonstrates PrEP protects against heterosexual transmission of HIV-1

The rationale for prevention of sexual HIV-1 acquisition with PrEP stems from efficacy of antiretrovirals for the prevention of mother-to-child transmission of HIV-1, first demonstrated with peripartum zidovudine [25]. Recent studies have shown that post-natal antiretrovirals, provided to infants who have ongoing exposure to HIV-1 through breastmilk, can substantially reduce HIV-1 risk [26]. Thus, these infant studies provided compelling analogous evidence that antiretroviral prophylaxis could be highly efficacious for preventing infection in the context of known and ongoing HIV-1 exposure [27].

Animal studies also provided evidence to suggest that antiretrovirals could be used for prevention of HIV-1 acquisition. Macaque SHIV challenge studies and more recently, humanized mouse HIV-1 challenge studies [28], have tested topical tenofovir gel, oral tenofovir disoproxyl fumarate (TDF), and oral combination emtricitabine (FTC)/TDF. TDF and FTC/TDF act early in the HIV-1 life cycle and have high potency, high genital tract levels, excellent safety and tolerability, and low incidence of resistance. Animal model studies have tested drug dosing and delivery routes that reflect oral dosing in humans and repeat low dose mucosal virus challenges to mimic sexual exposure to HIV-1. Overall, these studies indicated high levels of protection from topical tenofovir gel [29] and daily oral dosing of TDF and FTC/TDF, with potentially greater protection from combination FTC/TDF than TDF alone [30, 31], as well as efficacy when FTC/TDF was dosed intermittently (3 days before and 2 hours after rectal viral exposure) [32].

We are conducting the Partners PrEP Study, a phase III, placebo-controlled trial of oral tenofovir disoproxyl fumarate (TDF) and combination emtricitabine (FTC)/TDF PrEP among

HIV-1 uninfected partners in 4758 HIV-1 serodiscordant African couples. On July 10, 2011, the independent DSMB of the Partners PrEP Study recommended that the results of the trial be publicly reported and the placebo arm discontinued (more than 1.5 years early) because of definitive demonstration that PrEP substantially reduced HIV-1 risk, crossing a pre-specified statistical stopping boundary for the trial (Table 1); the findings were presented 8 days later [1]. HIV-1 protection from FTC/TDF and TDF was statistically similar (p=0.23), and each reduced HIV-1 in both men (TDF 63%, p=0.01 and FTC/TDF 84%, p<0.001) and women (TDF 71%, p=0.002 and FTC/TDF 66%, p=0.005). The rate of serious medical events was similar across the study arms. Further evidence for the efficacy of PrEP in the Partners PrEP Study is provided by a case-cohort analysis. For subjects on the active PrEP arms who acquired HIV-1 after randomization, 31% had tenofovir detected in a plasma sample at the seroconversion visit compared with 82% of a randomly-selected samples from a subset of subjects who did not acquire HIV-1, verifying overall high adherence in the trial and demonstrating that seroconverters had low adherence (Table 2, tenofovir was tested since it is common to the active arms). Importantly, having detectable tenofovir was associated with a relative risk reduction for acquiring HIV-1 of 86% (TDF, p<0.001) and 90% (FTC/TDF, p=0.002) – further emphasizing high protection against HIV-1 for those who were PrEP-adherent

Table 1. Partners PrEP Study efficacy results – July 2011 [1]

	TDF	FTC/TDF	Placebo
**HIV-1 incidence,** per 100 person-years	0.65	0.50	1.99
**HIV-1 protection efficacy, vs placebo**	**67%**	**75%**	
95% CI	(44-81%)	(55-87%)	
p-value	<0.001	<0.001	

Table 2. Case-cohort analysis

	Number / total samples (%) with tenofovir detected	
	**Case at seroconversion**	**Cohort**
**TDF arm**	6 / 17 (35.3%)	363 / 437 (83.1%)
**FTC/TDF arm**	3 / 12 (25.0%)	375 / 465 (80.6%)

The Partners PrEP Study results were a breakthrough in the prevention of heterosexual transmission of HIV-1 [5, 33] and reinforce findings of other recent PrEP studies: in CAPRISA 004 peri-coital tenofovir vaginal gel reduced HIV-1 risk by 39% (95% CI 6-60%, p=0.017) among 889 South African women [34]; in iPrEx, daily oral FTC/TDF reduced HIV-1 risk by 44% (95% CI 15-63%, p=0.005) among 2499 men who have sex with men [35], and oral FTC/TDF reduced HIV-1 risk by 63% (95% CI 22-83%, p=0.01) among 1200 young heterosexuals from Botswana in the TDF2 study [36]. Across studies, adherence has been a key predictor of efficacy; very high adherence in the Partners PrEP Study likely explains the high degree of HIV-1 protection. Notably, two PrEP trials among African women – FEM-PrEP (using FTC/TDF) [37] and VOICE (using TDF and tenofovir gel, with an FTC/TDF arm still ongoing) [38] – failed to show HIV-1 protection. For one of these (FEM-PrEP), substantial lack of adherence (approximately only 25% consistent use of the study medication) likely explains the failure to show HIV-1 protection. Thus, while a number of factors could explain the divergent trial results [5], foremost is non-adherence [39]. Importantly, for HIV-1 uninfected members of known HIV-1 serodiscordant couples, as shown in the Partners PrEP Study, adherence appears to be very high.

HIV-1 serodiscordant couples are central to the African epidemic and will be a target population for antiretrovirals for HIV-1 prevention

A clear and consistent message from stakeholders has been that a priority population for implementation of ART and PrEP for HIV-1 prevention will be HIV-1 serodiscordant couples

(i.e., one member is HIV-1 infected and the other uninfected). Population data from Africa suggest that a substantial fraction of new infections (up to half or more) may occur within stable serodiscordant marital or cohabiting relationships [40]. Epidemiologic studies, national HIV-1 serosurveys, and mathematical modeling analyses indicate that stable, heterosexual HIV-1 serodiscordant couples account for a substantial proportion of new HIV-1 transmissions in East Africa [40, 41]. The 2007 Kenya AIDS Indicator Survey (KAIS) found that 45% of married persons with HIV-1 had HIV-1 negative partners, and Uganda estimates a similar proportion [42]. Understanding HIV-1 prevention choices and targeting prevention strategies to this group are public health priorities. In particular, HIV-1 serodiscordant couples have been specifically identified as a priority population for implementation of antiretrovial-based HIV-1 prevention, given their high risk, smaller number for targeting relative to the general population, ability to be targeted for prevention efforts through promotion of couples HIV-1 counseling and testing, and clear advantage to the partnership to avert HIV-1 transmission. Importantly, during the past year, both ART (HPTN 052 [21]) and PrEP (Partners PrEP Study [43]) have demonstrated high efficacy for HIV-1 protection when used by members of HIV-1 serodiscordant couples. WHO is poised to release guidelines for counseling and HIV-1 prevention for HIV-1 serodiscordant couples, which will include ART and PrEP as potential prevention strategies.

Figure 2. HIV-1 incidence, by risk score

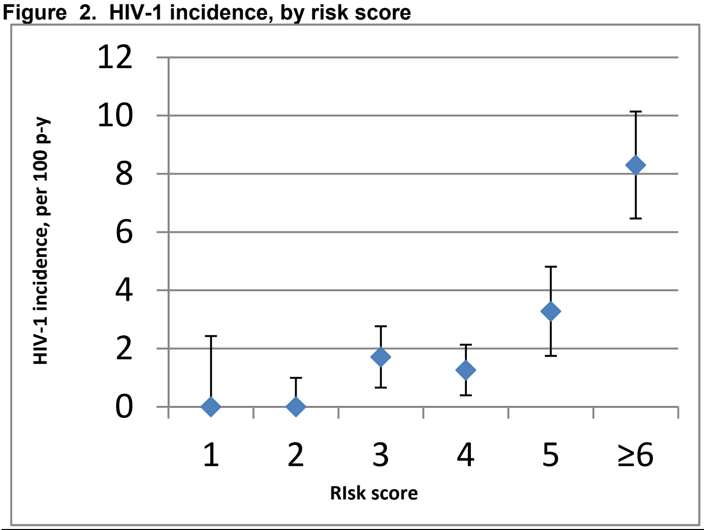

Risk score	Sero-conversions	Total couples	Person-years	Incidence (95% CI)
**1**	0	155	152.0	0.0 (0.0-2.4)
**2**	0	383	370.3	0.0 (0.0-1.0)
**3**	10	605	584.1	1.7 (0.7-2.8)
**4**	8	669	633.3	1.3 (0.4-2.1)
**5**	17	548	518.6	3.3 (1.7-4.8)
**≥6**	72	937	867.3	8.3 (6.5-10.14)

Targeting antiretroviral-based prevention to highest-risk couples

Defining those couples at highest risk for HIV-1 transmission might permit more efficient recruitment of couples into clinical research studies of novel prevention strategies and more cost-efficient delivery of expensive HIV-1 prevention interventions, such as ART and PrEP [15-18]. A simple risk assessment tool that could identify higher-risk couples would be useful for targeting evaluation and implementation of new HIV-1 prevention strategies, like antiretrovirals for prevention. Using data from three prospective studies of HIV-1 serodiscordant couples (>8500 couples) from 7 African countries and standard methods for development of clinical prediction rules, we derived and validated a risk scoring tool composed of key predictors for HIV-1 risk. The risk scoring tool was built off multivariate modeling using demographic, behavioral, clinical, and laboratory variables that could be measured in standard research and clinical settings. The final risk score included age of the HIV-1 uninfected partner, married and/or cohabiting partnership, number of children, unprotected sex, uncircumcised male HIV-1 uninfected partner, and HIV-1 plasma viral load. The maximum risk score was 12; overall, 28% of serodiscordant couples had an elevated risk score (≥6), and this group accounted for 67% of HIV-1 transmissions (Figure 2). A risk score of 5 was associated with an HIV-1 incidence of >3% per year; a score ≥6 was associated with an incidence of >5%. Internal and external validation within our discordant couples studies

datasets showed robust predictive ability of the risk score.

The results of this analysis demonstrate that a discrete set of factors, considered in combination and quantified to develop a risk score, can efficiently identify a subpopulation of HIV-1 serodiscordant couples at higher risk for HIV-1 transmission. The predictors selected for our final risk score model are well-established risk factors for HIV-1 and included factors measurable in clinical settings. Importantly, the combination of risk factors in a single algorithm allowed for more precise predictive capability than individual predictors. To our knowledge, the model defined here is the first empirically-based risk assessment tool for identifying high-risk HIV-1 serodiscordant heterosexual couples, and it offers a simple, quantitative approach for defining couples at higher HIV-1 risk. Our findings are relevant to both clinical research studies (to improve efficiency of recruitment) and programmatic roll-out of new HIV-1 prevention strategies (to maximize cost-effectiveness by targeting those at greatest risk) [20-22]. An example of our candidate risk scoring tool is presented in Figure 3.

Figure 3. Risk scoring tool

**Age of HIV-1 uninfected partner**			
	20 years or less	4	
	21-30 years	1	
	More than 30 years	0	
**Number of children**			
	0	2	
	1-2	1	
	3 or more	0	
**Male HIV-1 uninfected partner uncircumcised**			
	Yes	1	
	No	0	
**Married and/or cohabiting**			
	Yes	1	
	No	0	
**Unprotected sex within partnership, prior 30 days**			
	Yes	2	
	No	0	
**HIV-1 plasma viral load, HIV-1 infected partner**			
	50,000 copies or higher	3	
	10,000-49,999 copies	1	
	Less than 10,000 copies	0	
**Total score *(≥5-6 = higher risk)***			

Staged PrEP until ART initiation for HIV-1 prevention in couples

Given the constrained resources for HIV-1 treatment and prevention, many questions need to be considered regarding the relative benefits of PrEP and ART for HIV-1 prevention. We recently constructed a mathematical model to examine the impact and cost-effectiveness of different strategies, including earlier initiation of ART and/or PrEP, for HIV-1 prevention for serodiscordant couples [44]. The analysis provided three main results. First, PrEP used prior to ART initiation can prevent infections in HIV-1 serodiscordant couples and, although the initial costs are high, they are substantially offset by reduced future ART costs among HIV-1–uninfected partners who remain uninfected. In some circumstances (e.g., with effectiveness of ~80% -- approximately that seen in the Partners PrEP Study, particularly among those consistently using PrEP – and used in couples of high-risk), PrEP could be cost-saving overall. Second, PrEP in serodiscordant couples could be as cost-effective as earlier initiation of ART (compared to existing practice) if PrEP has a sufficiently high effectiveness (>70%) and low cost of delivery. If used in couples that remain at high risk, PrEP could be as cost-effective as earlier ART. Third, in couples that remain at high risk, PrEP and ART could be used sequentially (PrEP

in the uninfected individual prior to ART initiation for their HIV-1–infected partner) to deliver maximal benefit and best cost-effectiveness.

A key comparison of PrEP and ART is presented in Figure 4. The relative cost of PrEP to ART (vertical axis) and the effectiveness of PrEP (horizontal axis) are varied and the shaded region indicates the conditions where a PrEP intervention (PrEP used up to the moment that their infected partner starts treatment (at CD4 <350 cells/µL) is at least as cost-effective as earlier initiation of ART (at CD4 <500 cells/µL) at allowing couples to be "alive and HIV-1 free at age 50." The dark shaded region corresponds to lower risk couples (heavily counseled clinical trial participants from the Partners in Prevention HSV/HIV Transmission Study) and the lighter shaded region corresponds to higher-risk/more typical couples' behavior assumptions. The vertical dashed red line represents the efficacy estimate for FTC/TDF from the Partners PrEP Study – showing that, for higher-risk couples, PrEP used until the HIV-1 infected partner starts ART at <350 cells/µL is cost-effective, even if PrEP is equally expensive as ART.

Figure 4. Sequential use of PrEP and ART in higher risk couples

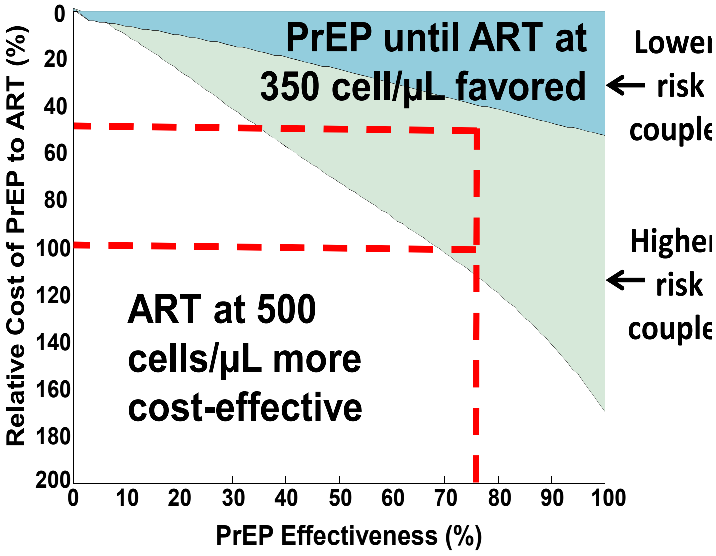

Ultimately, this work suggests that PrEP used prior to ART initiation can prevent infections in HIV-1 serodiscordant couples, with PrEP costs substantially offset by reduced future ART costs among HIV-1 uninfected partners who remain uninfected. Importantly, ART and PrEP are not antagonistic -- staged and strategic use of PrEP and ART could substantially and cost-effectively reduce HIV-1 transmission in HIV-1 serodiscordant couples. As international and national guidelines regarding ART and PrEP use evolve (potentially to even earlier ART initiation), staged PrEP use would still be important of HIV-1 uninfected partners of HIV-1 infected persons not yet eligible for ART, not yet willing to start ART, and recently started on ART (i.e., during the first few months before viral suppression is attained). In the Partners PrEP Study, for example, where all HIV-1 infected partners did not meet national guidelines for ART initiation at the time of enrollment but were actively referred for therapy upon meeting national guidelines, 20% of HIV-1 infected partners started ART during a median of 23 months of follow-up.

Need for implementation science research regarding delivery of ART and PrEP for HIV-1 prevention in couples

The first phase of PEPFAR provided ART access to >3.2 million HIV-1 infected persons. The second phase of PEPFAR aims to sustain initial successes and move from an emergency response to long-term solutions, which require critical evaluation of program effectiveness. The paradigm for studying translation of research evidence into service delivery is implementation science. A key priority for PEPFAR's implementation science agenda is translation of efficacy of antiretroviral-based HIV-1 prevention strategies – both ART to reduce infectiousness of HIV-1 infected persons and PrEP for uninfected persons to reduce HIV-1 acquisition – to

programmatic effectiveness. The key implementation science questions and methodologies related to this protocol are summarized in Table 3.

Table 3. Implementation science questions

Implementation questions	Methodology	Outcomes
Can HIV-1 serodiscordant couples characteristics predicting with ≥5% HIV incidence be readily recruited?	Operations research	Screened to enrolled ratio & HIV-1 incidence among HIV-1 serodiscordant couples who are recruited in new cohort
Will high-risk couples accept and use antiretroviral-based prevention, and what factors influence uptake?	Operations research	Uptake of PrEP as a bridge prior to ART initiation
Will high adherence be achievable w among those who receive PrEP in a public health model? Is adherence related to risk perception? Does PrEP use decline over time? What proportion return for refills?	Operations research	Multiple measures of adherence; mixed-methods approaches for risk perception, PrEP use, & adherence motivators & barriers
Will high levels of adherence be observed among HIV-1 infected persons who initiate ART while asymptomatic, in part motivated by desire to reduce transmission to their partner?	Operations research	Measurement of ART adherence by viral suppression; mixed-methods approaches for risk perception, ART use, & adherence motivators & barriers
Is there a significant reduction in # of HIV-1 infections averted through antiretroviral-based prevention for couples?	Impact evaluation	Comparison of HIV transmissions in the demonstration project compared to recent historical controls (placebo arm in Partners PrEP Study as counterfactual scenario)
What is the cost of delivery of antiretroviral-based prevention including PrEP? What is the cost-effectiveness of the PrEP bridging strategy?	Impact evaluation	Incremental cost-effectiveness of PrEP as a bridge to ART in reducing HIV-1 transmissions

Multiple questions remain about implementation of ART for HIV-1 prevention. Important questions need empiric data to inform resource allocation for ART, including uptake and adherence when ART is delivered to HIV-1 infected persons when asymptomatic and through typical clinical settings. For prevention benefit and clinical outcomes, ART adherence has to be high and sustained, resulting in viral suppression. A major focus is how to maximize coverage of ART for those eligible by national guidelines, and how to achieve durable adherence to ART among those who may have less motivation for adherence if they are asymptomatic. Issues of ART delivery models that achieve high adherence and retention outside of clinical trials are broadly relevant to ART delivery settings.

Implementation of PrEP will require targeting to high–risk persons with high motivation for adherence. Key deliverability questions for PrEP include targeting to those at highest risk, interest in PrEP use and motivators for sustained use (e.g., fertility intention, risk perception). Particularly needed is preparatory work in populations in which PrEP would be first targeted in an acceptable fashion in advance of larger-scale implementation. During the past several years, multiple stakeholder discussions have been held about how to deliver PrEP in various setting and populations. Recent US Food and Drug Administration approval of FTC/TDF PrEP for HIV-1 prevention – the first medication to achieve regulatory approval as an HIV-1 prevention intervention – has opened up new discussions about the potential for PrEP to become central to global HIV-1 prevention programs.

Targeted delivery of antiretroviral-based prevention to HIV-1 serodiscordant couples – a discrete and identifiable population – has been prioritized by PEPFAR and WHO, and is a first step to broader implementation. During mid-2012, WHO released guidance regarding next steps for PrEP evaluation – HIV-1 serodiscordant couples were prioritized, and demonstration projects were emphasized as the first step. As detailed above, a staged approach, with PrEP used by the uninfected partner until the infected partner initiates ART and typically achieves viral suppression may be most cost-effective and responsive to couples' preferences. For couples in which the HIV-1 infected partner has clinically-advanced disease, ART will be the priority. However, for couples in which the HIV-1 infected member is asymptomatic, with CD4 counts >350 cells/μL, or declines ART, empiric data are needed about the use of PrEP as a bridge to ART. Understanding the feasibility of targeting PrEP to couples with discrete risk characteristics

that predict they are at highest HIV-1 risk is particularly important, since PrEP delivery would be most cost-effective for averting infections in that subpopulation.

Finally, understanding ART and PrEP use in highest-risk couples is needed for anticipating maximal population-level benefits and cost-effectiveness. ART markedly reduces infectiousness, but not all HIV-1 risk for serodiscordant couples comes from within that partnership. Through analyses 'linking' transmission pairs by viral sequencing, studies have found that HIV-1 infected partners were not the source of transmission for ~30% of new infections in serodiscordant couples. PrEP would protect against HIV-1 transmissions both from within serodiscordant partnerships and from "outside" partners. Although dual use of PrEP and ART might provide the greatest HIV-1 protection, overlapping costs would be greatest in the that scenario. More likely,PrEP and ART will be staggered for couples – PrEP until the HIV-1 infected partner initiates ART – which may be both cost-effective and highly acceptable. Experience with discontinuing PrEP when a partner initiates ART is needed.

PrEP use during pregnancy

Pregnancy is a period of heightened HIV-1 risk for women -- likely reflecting both behavioral and biological contributions – and PrEP offers a potential risk-reduction strategy for high risk HIV-1 uninfected women when they are pregnant. In the Partners PrEP Study, PrEP was discontinued at the time pregnancy was detected; however, use until the time of pregnancy detection resulted in an estimated 4-8 weeks of exposure during early pregnancy. Overall, the pregnancy rate corresponded to 10.3 per 100 female partner subject-years. There were no statistically significant differences in pregnancy incidence or outcomes between women receiving PrEP and those receiving placebo (Table 4). Additionally, no difference or pattern of congenital abnormalities was noted.

Table 4. Pregnancy incidence and outcomes, Partners PrEP Study

	TOTAL	TDF	FTC/TDF	Placebo
**Number of pregnancies**	**288**	112	80	96
**Pregnancy incidence,** per 100 woman-years	**10.3**	11.9	8.8	10.0
P-value, vs. placebo		*0.19*	*0.40*	
**Pregnancy outcome**<br>**Live birth**<br><br>**Pregnancy loss**	 **167**<br>**(64%)**<br>**95**<br>**(36%)**	 73<br>(71%)<br>30<br>(29%)	 40<br>(54%)<br>34<br>(46%)	 54<br>(64%)<br>31<br>(36%)
P-value, vs. placebo		*0.35*	*0.26*	

The FDA-approved label for FTC/TDF (Truvada®) summarizes risks and benefits related to use of this medication in pregnancy:

Risk Summary
TRUVADA has been evaluated in a limited number of women during pregnancy and postpartum. Available human and animal data suggest that TRUVADA does not increase the risk of major birth defects overall compared to the background rate. There are, however, no adequate and well-controlled trials in pregnant women. Because the

studies in humans cannot rule out the possibility of harm, TRUVADA should be used during pregnancy only if clearly needed. If an uninfected individual becomes pregnant while taking TRUVADA for a PrEP indication, careful consideration should be given to whether use of TRUVADA should be continued, taking into account the potential increased risk of HIV-1 infection during pregnancy.

Moreover, one of the key population groups identified, by both the FDA and the WHO, for priority data collection regarding PrEP use is women using PrEP throughout pregnancy. In guidance produced by the US Centers for Disease Control and Prevention, women who become pregnant while using PrEP should discuss with their health-care providers the "currently available information regarding potential risks and benefits of continuing PrEP so that an informed decision can be made." Providers are also encouraged to submit information about the pregnancy to the Antiretroviral Use in Pregnancy Registry, which is also standard practice for de-identified clinical trial pregnancy data.

In the approval of FTC/TDF PrEP for HIV-1 prevention, the U.S. FDA emphasized the need for safety data among pregnant women:

"Through collaboration with the Antiretroviral Pregnancy Registry, a prospective observational study should be conducted in order to collect and analyze data on maternal and fetal outcomes in 200 women who become pregnant while taking Truvada® [FTC/TDF] for a pre-exposure prophylaxis (PrEP) indication and choose to continue Truvada® during their pregnancies and in 200 women who become pregnant while taking Truvada® for PrEP and choose to discontinue it."

Thus, understanding PrEP use and safety in HIV-1 uninfected women who become pregnant while using PrEP is a priority, including whether or not women will choose to continue using PrEP. Importantly, a critical consideration is the assessment of whether women who choose to discontinue PrEP during pregnancy face new risk of HIV-1 infection, which risks transmitting HIV-1 to their child.

Summary

Multiple stakeholder discussions have noted that demonstration of deliverability and understanding adherence and sexual behavior in the context of ART for prevention and PrEP use outside of clinical trials are critical factors to consider in anticipating roll-out of these strategies. HIV-1 serodiscordant couples are a primary target for implementation of ART for prevention and PrEP, particularly for East Africa. Critical unanswered questions for successful implementation of antiretroviral-based HIV-1 prevention include how to target these expensive prevention strategies to realize maximum population HIV-1 prevention benefits and whether HIV-1 infected persons with asymptomatic disease would accept ART to reduce their risk for transmitting HIV-1, at-risk HIV-1 negative persons would use PrEP, and both would sustain high adherence needed for high effectiveness. Couples may potentially have the opportunity to use ART or PrEP for prevention; however, costs would prohibit simultaneous use in most settings. Thus, staged use – i.e., PrEP until the HIV-1 infected partner initiates ART and achieves viral suppression – may be an effective and cost-effective approach [44], and it is the approach we propose. Successful implementation of ART and PrEP for prevention in HIV-1 serodiscordant couples will need to 1) target delivery to highest-risk couples, 2) respond to couples' preferences for and barriers to use of PrEP and ART, 3) achieve high uptake and sustained adherence and 4) consider how to discontinue PrEP if the HIV-1 infected partner initiates ART. This study proposes to address these key questions.

III. STUDY METHODS

Overall Design.

This is an open-label, prospective cohort study of higher-risk HIV-1 serodiscordant couples. The overall goal is to determine barriers and facilitators to uptake and sustained adherence to ART for HIV-1 infected partners and daily oral PrEP for HIV-1 uninfected partners. Our validated risk score will be used to target couples at highest risk of HIV-1 transmission. We will offer PrEP (daily oral FTC/TDF) to the HIV-1 uninfected partner and measure uptake and adherence. We will refer HIV-1 infected participants to ART services following national ART initiation guidelines, recognizing that guidelines for ART initiation may change during the course of the study. For each couple, if the HIV-1 infected partner initiates ART, we will discontinue PrEP for the HIV-1 uninfected partner six months later (i.e., once viral suppression is typically achieved). If the HIV-1 infected partner declines to initiate ART, or initiates and discontinues, we will continue PrEP. We will conduct mixed-methods work to understand user preferences, couples decision-making, and barriers to uptake of and adherence to both PrEP and ART.

Objectives.

Aim 1: We will evaluate the ability to do targeted enrollment of high-risk HIV-1 serodiscordant couples into a longitudinal HIV-1 prevention study

A critical aspect of implementation and cost-effectiveness of antiretroviral-based HIV-1 prevention in HIV-1 serodiscordant couples will be the ability to utilize behavioral, epidemiologic and clinical data to identify those couples who are at highest risk of HIV-1 acquisition. We have developed a risk score using enrollment data from our previous studies of >8500 HIV-1 serodiscordant couples that is able to discriminate couples at highest risk. The score is comprised of a limited set of variables that can be obtained in research and clinical settings: plasma HIV-1 levels in the HIV-1 infected partner, any unprotected sex in the couple in the prior month, number of children in the partnership, age of the HIV-1 uninfected partner, and circumcision status of HIV-1 uninfected male partners.

The primary outcomes of this aim will be the screened to eligible ratio of HIV-1 serodiscordant couples recruited for this open-label study, the proportion of eligible couples who decide to enroll in the cohort, and the costs of screening and targeting high-risk couples.

Aim 2: We will assess user preferences among high-risk HIV-1 serodiscordant couples for ART initiation for HIV-1 infected partners and PrEP for HIV-1 uninfected partners.

Understanding couples' preferences for and concerns about antiretroviral-based HIV-1 prevention is of utmost importance at this time when guidelines for its use are being formulated.

The primary outcomes for this aim will be measured at baseline and quarterly through an interviewer-administered questionnaire. The questionnaire will specifically ask about willingness to use PrEP or ART for HIV-1 prevention and

which method the participant would prefer. Reasons for the choice and concerns about both methods will also be collected through mixed-methods approaches.

Aim 3: We will ascertain initiation of and adherence to PrEP among HIV-1 uninfected partners, as a bridge to ART.

Understanding adherence to PrEP is a critical research priority, particularly when PrEP is deliveredoutside of a controlled clinical trial setting. Brief adherence counseling for all participants will be delivered at baseline and then quarterly thereafter. The content of the adherence counseling will be derived from the counseling messages and procedures in the Partners PrEP Study, and informed by the findings from an ancillary adherence study conducted as part of the Partners PrEP Study. PrEP will be implemented as a bridge to ART, as described in the Summary and Figure 1.

The primary measures of adherence will be collection of PrEP, clinic-based pill counts of unused study pills as recorded at each follow-up visit, and MEMS cap data on how frequently the pill bottle was opened. Blood samples for detection and quantification of PrEP levels (testing in batch) will be done for those who seroconvert to HIV-1.

Aim 4: We will ascertain initiation of and adherence to ART among HIV-1 infected partners.

In addition to providing clinical benefit to HIV-1 infected partners, ART also confers HIV-1 prevention benefits to HIV-1 uninfected partners. HIV-1 infected partners who become eligible for ART will be actively encouraged to initiate, including counseling to improve their knowledge and understanding of ART and the benefits for themselves and their partner and the importance of strict adherence to ART regimens to achieve viral suppression.

The primary measures of ART adherence will be ART initiation at a local ART provider and plasma HIV-1 RNA levels taken every 6 months for HIV-1 infected partners.

Aim 5: We will assess factors influencing preferences, uptake and adherence for antiretroviral-based HIV-1 prevention

Understanding factors that influence couples' preferences and their likelihood to initiate and adherence to PrEP and/or ART in a real-world setting is critical for providers who will counsel HIV-1 serodiscordant couples and make recommendations about the HIV-1 prevention practices that are best indicated for couples.

Through interviewer-administered questionnaires, correlates of preferences, uptake and adherence to ART and PrEP will be assessed, including characteristics of sexual risk (i.e., sexual frequency, condom use, whether the HIV-1 uninfected partner reports outside partners), depression and substance use, gender of the HIV-1 uninfected partner, fertility intentions, partnership characteristics (whether the couple is cohabiting at baseline, has children, is together throughout follow-up, whether members have other partners, and whether the HIV-1 infected partner

initiates ART), and clinical characteristics of the HIV-1 infected partner (e.g., CD4 count, WHO stage).

Aim 6: We will assess the feasibility of PrEP discontinuation in couples in which the HIV-1 infected partner initiates ART

In couples in which the HIV-1 infected partner initiates ART, we will discontinue PrEP six months later. It is possible that HIV-1 uninfected partners may be reluctant to discontinue PrEP, for example if they do not trust their partner's motivation to consistently use ART or if the uninfected partner has additional sexual partners.

Through interviewer-administered questionnaires, we will assess couples' attitudes and understanding of PrEP discontinuation.

Aim 7: We will assess PrEP use and birth outcomes among HIV-1 uninfected women who choose to continue PrEP during pregnancy

Pregnancy represents a period of heightened HIV-1 risk for women, and for their infant if acute HIV-1 infection occurs. Preventing HIV-1 acquisition in pregnant women is a priority. Data from PrEP clinical trials and from clinical experience with FTC/TDF as part of combination antiretroviral therapy for HIV-1 infected women have shown that FTC/TDF does not appear to increase the risk of major birth defects. A priority for high-risk HIV-1 uninfected women is to assess PrEP use during pregnancy.

HIV-1 uninfected women who become pregnant will consented to monthly follow-up during pregnancy - regardless of their choice to continue or discontinue PrEP. We will monitor for HIV-1 seroconversion, serious adverse events, congenital malformations, and infant growth.

Population.

Heterosexual HIV-1 serodiscordant couples will be recruited from Partners PrEP Study sites in Kenya and Uganda. To recruit a population that has not experienced PrEP use, only couples who did not participate in the Partners PrEP Study will be recruited.

Study services

Participants will receive baseline and quarterly individual and couples HIV-1 counseling, condoms, risk reduction counseling, and syndromic management of sexually transmitted infections according to local guidelines. HIV-1 infected partners will have quarterly monitoring of HIV-1 clinical status and 6-monthly CD4 counts and will be referred for HIV-1 care based on national HIV-1 care guidelines. Counseling on the importance of not sharing study medications within the partnership will occur at quarterly visits. To ensure as real world assessment of uptake and sustained use of PrEP and ART as possible, tracing of participants will occur only for follow-up of safety issues and for HIV-1 assessment at the end of the study, but not for completion of routine visits and PrEP refills. HIV-1 uninfected women using PrEP who become pregnant will be offered the opportunity to continue PrEP and will participate in more frequent follow-up, including follow-up of their infants.

Eligibility.

For couples

- Risk score defining higher HIV-1 risk (≥5, as defined by card in Figure 3)
- Sexually active (defined as having had vaginal intercourse at least 6 times in the previous three months)
- Willing to enter the study as a couple and intending to remain as a couple for the next 12 months
- Did not participate in the Partners PrEP Study

For HIV-1 uninfected members of the couple (partner participants)

- Age ≥18
- Able and willing to provide written informed consent
- HIV-1 uninfected based on negative HIV-1 rapid tests, both at study screening and at the enrollment visit
- Adequate renal function, defined by normal creatinine levels and estimated creatinine clearance ≥60 mL/min
- Not infected with hepatitis B virus, as determined by a negative hepatitis B surface antigen test
- Not currently pregnant or breastfeeding
- Not currently enrolled in an HIV-1 prevention clinical trial
- Not currently using PrEP
- Enrollment of individuals with active and serious infections or active clinically significant medical problems will be at the discretion of the site investigator

For HIV-1 infected members of the couple (index participants)

- Age ≥18
- Able and willing to provide written informed consent
- HIV-1 infected based on positive rapid HIV-1 tests, according to national algorithm
- No history of WHO stage III or IV conditions
- Not currently using ART
- Not currently enrolled in an HIV-1 treatment study
- *Note: current pregnancy and breastfeeding are permitted for HIV-1 infected partners*

Sample size.

Up to 500 couples per site. All couples will participate in the longitudinal cohort study; a subset (up to 60 per site) will be invited to participate in qualitative in-depth interviews and focus group discussions.

PrEP medication

Tenofovir disoproxil fumarate (or TDF, 9-[(R)-2-[[bis[[(isopropoxycarbonyl)oxy]methoxy]phosphinyl] methoxy]propyl]adenine fumarate) and emtricitabine (or FTC, 5-fluoro-1-(2R,5S)-[2-(hydroxymethyl)-1,3-oxatholan-5-yl]cytosine) are reverse transcriptase inhibitors that have been approved for the treatment of HIV-1 infection in humans in Kenya, Uganda, and the United States. A fixed-dose, oral co-formulation of FTC/TDF (Truvada®) will be used in this study. PrEP will be prescribed for once-daily use. Study medication will be donated by Gilead Sciences.

Recruitment

Each site has established local recruitment and screening methods that operationalize protocol-specified requirements for eligibility determination in a manner that is tailored to and most efficient for the local study setting and target study population.

Recruitment strategies will include partnering with existing voluntary counseling and testing (VCT) centers and outreach workers, public promotion of couples VCT by well-known figures and community organizations such as churches, and community mobilization around couples VCT promotion (e.g., around Valentine's Day). Recruitment materials will educate couples about the probability of being HIV-1 serodiscordant based on available data, and risks of unknown HIV-1 serodiscordancy in terms of transmission to the HIV-1 uninfected partner, and will emphasize the benefits of couples VCT with specialized counseling services. Couples may be recruited for possible inclusion in the study through referrals from VCT centers and other community-based organizations, direct outreach, or other activities conducted at the study sites.

Regardless of the recruitment source, each partner in each couple will provide independent informed consent for screening. The screening process will proceed in a step-wise manner for both partners until either all screening procedures are completed or one or both of the partners is determined to be ineligible.

Although all required screening procedures may be completed in as few as two visits for each partner, additional visits may be conducted as needed (for example, if one or both partners want more time to consider whether to enroll in the study). At least one screening visit and the enrollment visit must be attended by both partners of a couple together, and at least one couples counseling session must take place during the screening process. There is no time limit on the screening process.

For those couples found to be eligible for the study, informed consent for study participation and enrollment in the study may proceed on the same day when eligibility is determined. Each partner will be asked to provide independent informed consent for study participation.

Study procedures

Specific study procedures are detailed in Tables 5-9. Visits will take place at screening and enrollment, a visit 1 month after enrollment, and then quarterly thereafter, for up to 24 months.

At screening, demographic and behavioral information will be collected, along with laboratory results to establish the risk score and participant eligibility (for HIV-1 uninfected partners – serum creatinine, hepatitis B surface antigen; for HIV-1 infected partners – CD4 count, plasma HIV-1 viral load; for both partners – HIV-1 rapid testing according to national algorithms).

Note: couples with a score <5 will be counseled about HIV-1 serodiscordancy, the importance of behavioral risk-reduction and condoms to minimize transmission risk, and will be referred to partner organizations for couples support groups and HIV-1 clinical care and ART according to national guidelines; in this way, a score of <5 will not be interpreted as "low risk." Our goal with highest-risk recruitment for this cohort is to show that antiretroviral-based prevention can be implemented among those who could achieve the greatest benefit.

At enrollment, HIV-1 testing will be performed for HIV-1 uninfected partners, to confirm eligibility (HIV-1 seronegative at the time of study start). Couples will be counseled about ART and PrEP.

HIV-1 uninfected partners will be offered PrEP; HIV-1 infected partners will be counseled about ART guidelines and will be referred/initiated on ART if they are eligible at the time of enrollment or will be counseled how they will be referred for initiation if they were to become eligible for ART during the study. HIV-1 uninfected partners who have symptoms potentially consistent with acute HIV-1 infection (fever, rash, pharyngitis) will have enrollment deferred for at least 2 weeks at which time repeat serologic testing will be performed (and, if positive, will result in study exclusion). Adherence, risk reduction and contraceptive counseling will be completed. HIV-1 uninfected participants will be given MEMS caps to electronically capture each date and time that their PrEP pill bottle is opened. Data from MEMS caps will be downloaded during quarterly visits as part of routine PrEP dispensation procedures. Additionally, behavioral and medical history data as well as data on participant preferences regarding antiretroviral-based prevention strategies and fertility intentions will be collected during one-on-one interviews with staff counselors. Syndromic diagnosis and management of STIs will be conducted as well as a physical exam for both members of the couple. A blood sample from HIV-1 uninfected partners will be collected to be used for retrospective PCR testing if the participant seroconverts early during follow up.

At enrollment HIV-1 uninfected women will have a pregnancy test to confirm eligibility (must be not pregnant to complete enrollment). During follow-up, women will be queried regarding pregnancy at every scheduled visit and pregnancy testing will be done when clinically indicated (e.g., missed menses, participant request). Women who become pregnant while using PrEP will be offered a separate consent to enroll in enhanced follow-up (monthly) during pregnancy and to continue PrEP during pregnancy. Infants born to these women will be followed quarterly until 1 year of age. [Of note, these procedures reflect the follow-up schedule of the Partners PrEP Study clinical trial.]

At the Month 1 and then quarterly visits, participants will be counseled (for adherence, not sharing PrEP medications, risk reduction and contraception) and complete behavioral and medical history interviews with staff counselors. Syndromic diagnosis and management of STIs will be conducted as clinically indicated. At each scheduled visit, a plasma sample will be archived, for testing including tenofovir levels (as an objective measure of PrEP adherence).

Every 6 months, in addition to regular quarterly visit procedures, CD4 counts and plasma viral loads will be done for HIV-1 infected partners.

If HIV-1 infected partners become eligible for ART under current national guidelines, they will be offered ART on-site or referred to local clinics to seek ART services and initiate ART. If national guidelines for ART initiation change during the course of the study (for example, initiation at higher CD4 counts or inclusion of plasma viral load in initiation decisions), the study will follow updated guidelines as they are implemented. Once the HIV-1 infected partner has been using ART for 6 months, PrEP will be discontinued for the HIV-1 uninfected partner, after counseling to the couple.

During follow-up, HIV-1 infected and uninfected participants will complete a quantitative interview to capture evolution in preferences to uptake of antiretroviral-based HIV-1 prevention and fertility intentions. Questionnaires will be administered separately to both members of couples.

For initially HIV-1 uninfected participants who seroconvert during the study, PrEP will be discontinued. Seroconverters will have a visit within 1-month after they seroconvert and then follow-up in the study will be completed for both members of the couple. These visits will

include risk reduction and contraceptive counseling, collection of behavioral and medical history data during one-on-one interviews, and collection of blood specimens for assessment of CD4 count, HIV-1 plasma viral load levels and batched resistance assays from both partners. In addition, serum, plasma and peripheral blood mononuclear cells (PBMCs) will be archived for possible genotypic and phenotypic resistance testing, determining the level of PrEP drug present in the blood and immune function.

Interim visits may occur at any time during the study. All interim contacts and visits will be documented in participants' study records and on applicable CRFs. If a study participant presents to an interim visit with symptoms suggestive of acute HIV-1 infection syndrome, rapid HIV-1 testing will be performed, as at a scheduled visit, and study medication will be held if a positive rapid test is documented.

All data collection will be conducted in a private room in the participant's preferred language, according to the participant's fluency and preference. Development of data collection tools has been guided by our experience collecting clinical data for the Partners PrEP Study at multiple sites in Kenya and Uganda as well as results from a linked study using qualitative interviews and focus group discussions conducted at the Thika, Kenya study site with members of HIV-1 serodiscordant couples.

HIV-1 testing

HIV-1 testing – for index (HIV-1 infected) participants at screening and for partner (HIV-1 uninfected) participants at screening, enrollment, and follow-up – will be performed in line with national HIV-1 testing algorithms for Kenya and Uganda. HIV-1 seroconversions will be confirmed using HIV-1 EIA. HIV-1 testing will be accompanied by couples and/or individual counseling, depending on attendance of the index (HIV-1 infected) participant at a study visit. All counseling and testing approaches will be in accordance with national HIV-1 counseling and testing guidelines. For women who become pregnant and choose to enroll for enhanced follow up, HIV-1 EIA and PCR testing will be done during the third trimester, in addition to the routine serologic testing, in order to detect acute infection during late pregnancy. Babies born to any of these women who seroconvert during pregnancy will be tested for HIV-1 according to national guidelines.

Table 5. Procedures for partner (HIV-1 uninfected) participants

	S	E	M1	M3 & M15	M6 & M18	M9 & M21	M12 & M24
**ADMINISTRATIVE AND REGULATORY PROCEDURES**							
Obtain informed consent	X	X					
Apply inclusion/exclusion criteria, including behavioral and lab eligibility	X	X					
Collect/update locator information	X	X	X	X	X	X	X
Collect/update demographic information	X						X
**COUNSELING**							
Provide HIV-1 pre and post-test counseling	X	X	X	X	X	X	X
Risk reduction counseling and condom promotion & provision	X	X	X	X	X	X	X
Contraception counseling and provision/referral	X	X	X	X	X	X	X
Adherence counseling		X	X	X	X	X	X
**CLINICAL PROCEDURES**							
Provide HIV-1 test results	X	X	X	X	X	X	X
Acute HIV-1 assessment		X					
Collect prior PrEP supplies for pill count, download MEMS data			X	X	X	X	X
Medical history / symptoms information	X	X	X	X	X	X	X
Perform physical exam		X	[X]	[X]	[X]	[X]	[X]
STI syndromic assessment and management		X	[X]	[X]	[X]	[X]	[X]
Collect blood specimen (screening laboratory tests, enrollment sample, and follow-up samples for archival to measure PrEP adherence)	X	X	X	X	X	X	X
Offer and provide PrEP, instructions; discontinue PrEP 6 months after HIV-1 infected partner initiates ART		X	X	X	X	X	X
**BEHAVIORAL DATA COLLECTION**							
Collect sexual behavioral information	X	X	X	X	X	X	X
Collect alcohol and substance use data		X					X
Collect depression indicators		X					X
Collect HIV-1 risk perception data		X	X	X	X	X	X
Collect antiretroviral-based prevention preference data, information on fertility intentions, stigma and social support, and other sociobehavioral data to inform PrEP and ART preferences and use; at visit at which PrEP is stopped, questionnaire about PrEP discontinuation		X	X	X	X	X	X
Collect adherence data			X	X	X	X	X
Collect data on understanding and attitudes regarding PrEP discontinuation, for those stopping PrEP 6 months after HIV-1 infected partner initiates ART				[X]	[X]	[X]	[X]
**LABORATORY PROCEDURES**							
Creatinine	X		X		X		X
HIV-1 serology (rapid test and, if positive, EIA)	X	X	X	X	X	X	X
HBV surface antigen	X						
Urine pregnancy test (women only, at enrollment and then as clinically indicated)		X	[X]	[X]	[X]	[X]	[X]

[ ] as indicated

Table 6. Procedures for index (HIV-1 seropositive) participants

	S	E	M1	M3 & M15	M6 & M18	M9 & M21	M12 & M24
**ADMINISTRATIVE AND REGULATORY PROCEDURES**							
Obtain informed consent	X	X					
Apply inclusion/exclusion criteria, including behavioral and lab eligibility	X	X					
Collect/update locator information	X	X	X	X	X	X	X
Collect/update demographic information	X						X
**COUNSELING**							
Provide HIV-1 pre and post-test counseling	X						
Risk reduction counseling and condom promotion & provision	X	X	X	X	X	X	X
Contraception counseling and provision/referral	X	X	X	X	X	X	X
Adherence counseling (if on ART)			X	X	X	X	X
Counseling regarding ART eligibility, clinical and prevention benefits of ART	X	X	X	X	X	X	X
**CLINICAL PROCEDURES**							
Provide HIV test results	X						
Medical history	X	X	X	X	X	X	X
Perform physical exam		X	[X]	[X]	[X]	[X]	[X]
STI syndromic assessment and management		X	[X]	[X]	[X]	[X]	[X]
Collect blood specimen (screening lab tests, 6-monthly CD4 and viral load)	X				X		X
Referral for / provision of ART according to national guidelines		X	X	X	X	X	X
**BEHAVIORAL DATA COLLECTION**							
Collect sexual behavior information	X	X	X	X	X	X	X
Collect alcohol and substance use data		X					X
Collect depression indicators		X					X
Collect HIV-1 risk perception data	X	X	X	X	X	X	X
Collect antiretroviral-based prevention preference data, information on fertility intentions, stigma and social support, and other sociobehavioral data to inform PrEP and ART preferences and use	X	X	X	X	X	X	X
Collect adherence data			X	X	X	X	X
Collect data on understanding and attitudes regarding PrEP discontinuation, for those stopping PrEP 6 months after HIV-1 infected partner initiates ART				[X]	[X]	[X]	[X]
**LABORATORY PROCEDURES**							
HIV-1 serology	X						
CD4 count	X				X		X
Plasma HIV-1 viral load	X				X		X
Urine pregnancy test (women only, as clinically indicated)		[X]	[X]	[X]	[X]	[X]	[X]

[ ] as indicated

Table 7. Procedures for HIV-1 seroconverters

	Possible seroconversion visit (≥1 rapid HIV-1 test positive)	Follow-up to possible seroconversion (ideally within one month of possible seroconversion)
**ADMINISTRATIVE, BEHAVIORAL AND REGULATORY PROCEDURES**		
Provide HIV-1 counseling, including couples counseling	X	X
**CLINICAL PROCEDURES**		
Collect medical history	X	X
Perform physical exam	X	
Collect blood specimen	X	X
Stop PrEP, if applicable	X	
Provide test results	X	X
Refer for HIV-1 care		X
**LABORATORY PROCEDURES**		
CD4 count	X	X
HIV-1 plasma viral load	X	X
HIV-1 serology (confirmatory EIA)	X	
Urine pregnancy test (women only)	X	X
Serum, plasma and PBMC archive for HIV-1 resistance assays, PrEP levels (batched testing)	X	X

Index participants whose partners seroconvert to HIV-1 will have one set of samples collected as close in time to the seroconversion visit as possible (ideally, either at the SC or SC<1 month visits). Samples will be collected:

CD4 count
Viral load
Plasma archive (for tenofovir and resistance assays)

Table 8. Procedures for female partner (HIV-1 uninfected) participants during pregnancy*

	Visit when pregnancy is discovered	Monthly visits during pregnancy
**ADMINISTRATIVE AND REGULATORY PROCEDURES**		
Obtain informed consent	X	
Collect/update locator information	X	X
Collect/update demographic information	X	X
**COUNSELING**		
Provide HIV-1 pre and post-test counseling	X	X
Risk reduction counseling and condom promotion & provision	X	X
Discussion of PrEP use during pregnancy – known risks & benefits	X	X
Adherence counseling	X	X
**CLINICAL PROCEDURES**		
Provide HIV-1 test results	X	X
Acute HIV-1 assessment	X	X
Collect prior PrEP supplies for pill count, download MEMS data	X	X
Medical history / symptoms information	X	X
Perform physical exam	[X]	[X]
STI syndromic assessment and management	[X]	[X]
Collect blood specimen (screening laboratory tests, enrollment sample, and follow-up samples for archival to measure PrEP adherence)	X	X
Offer and provide PrEP, instructions	X	X
**BEHAVIORAL DATA COLLECTION**		
Collect sexual behavioral information	X	X
Collect alcohol and substance use data	During annual visits, on schedule already established	
Collect depression indicators	During annual visits, on schedule already established	
Collect HIV-1 risk perception data	X	X
Collect antiretroviral-based prevention preference data, information on fertility intentions, stigma and social support, and other sociobehavioral data to inform PrEP and ART preferences and use; at visit at which PrEP is stopped, questionnaire about PrEP discontinuation	X	X
Collect adherence data	X	X
**LABORATORY PROCEDURES**		
HIV-1 serology (rapid test and, if positive, EIA)	X	X
Creatinine	Every 6 months, on schedule already established	
HIV-1 EIA and PCR		Once, during 3rd trimester

[ ] as indicated

*for women who consent for enhanced follow-up during pregnancy.

Table 9. Procedures for infants born to HIV-1 uninfected women using PrEP at the time of pregnancy

	Visit <1 month after birth	Quarterly visits for 12 months
**ADMINISTRATIVE AND REGULATORY PROCEDURES**		
Collect/update locator information	X	X
Collect/update demographic information	X	X
**CLINICAL PROCEDURES**		
Medical history / symptoms information	X	X
Perform physical exam	X	X
**LABORATORY PROCEDURES**		
HIV-1 DNA PCR for babies born to women who seroconvert	Per national policies	

Participant retention and withdrawal

For this observational demonstration project, where the goal is to reflect real-world delivery of PrEP and ART, formal retention efforts will reflect retention approaches used by HIV-1 care centers in Kenya and Uganda. Each site will develop retention methods tailored to and most efficient for the local study setting. Retention activities may include explanation of the study visit schedule and procedural requirements during the informed consent process and re-emphasis at each study visit, collection and updating of locator information, and use of appropriate and timely visit reminder mechanisms (including phone calls and text messages). To provide complete information at the end of the study, efforts will be made to have a final follow-up visit for each participant.

For pregnant women who agree to have more frequent follow-up visits during their pregnancy, additional retention efforts may be done.

Participants may voluntarily withdraw from the study for any reason at any time. The site Investigator also may withdraw participants from the study in order to protect their safety and/or if they are unwilling or unable to comply with required study procedures. Reasons for withdrawal, including partnership dissolution, will be recorded.

Qualitative data collection. To gain a deeper understanding of the factors and the extent to which the factor influences the willingness of the infected partner to initiate ART or uninfected partner to use PrEP regularly, we will conduct in-depth interviews and focus group discussions with up to 60 couples per site (some interviews will be individually-focused and some will be couples-based). Interview topics will include: identification of factors influencing individuals' preferences for antiretroviral-based HIV-1 prevention, their experience using antiretroviral-based HIV-1 prevention and their intentions for continued use of antiretroviral-based HIV-1 prevention and potential strategies to reduce HIV-1 risk when they desire to conceive a child. We will conduct purposive sampling to include couples with HIV-1 infected women and men across a spread of ages and baseline CD4 counts. Interviews will take 60-120 minutes and will focus on structural (contraception access, gender roles), individual (fertility desire, ability to discuss fertility with partner, understanding serodiscordance), and dyadic (sexual negotiation skills, trust, power, violence) factors; a subset of individual interviewees will be selected for follow-up interviews. Interviews done with the couple together will assess the couples' dynamic and communication patterns. Interviews will be conducted in the participant's preferred language at a time mutually convenient for the participant(s) and study staff and they will be recorded and transcribed. Neither the transcribed notes nor the facilitator notes from the focus group will include any personal identifiers. Interviewers will follow pre-piloted topic guides, developed through literature review and our ongoing qualitative and quantitative research, with flexibility to explore probes and relevant content. Discussion of sexual assault and suicidal ideation will be avoided. The audio recordings and transcriptions from the interviews will be destroyed after study participation and data analysis has been completed, by September 1, 2018.

IV. PrEP MEDICATION

PrEP medication

This study will provide open-label PrEP to HIV-1 uninfected individuals (partner participants) in HIV-1 discordant couples. Co-formulated FTC/TDF at 200 mg / 300 mg respectively will be used. The dose of FTC/TDF is the standard dose approved by the U.S. Food and Drug Administration.

It has been demonstrated that FTC/TDF tablets are stable at 25°C and 60% relative humidity for up to 48 months, at 30°C and 65% relative humidity for up to 24 months, and at 40°C and 75% relative humidity for up to 6 months. Additionally, tablets have been shown to be stable at 50°C for up to 1 month.

FTC/TDF tablets will be supplied by Gilead. Tablets will be packaged as a one-month supply (30 tablets) in bottles with child-resistant screw caps. In addition to the tablets, each bottle contains a silica gel desiccant to protect the product from humidity and the polyester packing material that cushions it during handling and shipping. PrEP will be dispensed in an amount to last until the next scheduled visit – thus, at enrollment, a one-month supply (to last until the scheduled Month 1 visit), a two-month supply at Month 1 (to last until the Month 3 visit), and a three-month supply thereafter (corresponding to quarterly visits).

The study drug will be stored in accordance with the drug manufacturer's recommendations. The pharmacy and storage facility will have locked, climate-controlled environments, with controlled humidity and temperature to remain within limits allowed by the manufacturer for drug storage.

Counseling on the medications being used, their side effect profiles, how to take the study

medication, what to do if side effects are experienced, and the importance of not sharing study medication to optimize potential efficacy and to reduce the chances of developing resistance through suboptimal HIV-1 suppression if study medication is shared with the index (HIV-1 infected) partner or with others (including other spouses – e.g., a 2nd wife – if applicable) will occur at each study visit. Women who become pregnant while taking PrEP will be counseled about the potential for increased HIV-1 risk during pregnancy and currently available information regarding potential risks and benefits of continuing PrEP.

Adherence.

High adherence is important for PrEP effectiveness in preventing HIV-1 acquisition. Study staff also will provide brief adherence counseling at each scheduled visit, to mimic “real world” counseling.

Data on adherence to the product use regimen will be collected via standardized interviewer-administered questions to ascertain product use. Additionally, staff will count pills remaining in bottles returned to the study site and record the number of pills returned. MEMS caps will be used as well. Finally, adherence will also be assessed through batched drug levels in the partner (HIV-1 uninfected) participants from time points as described above.

Discontinuation of PrEP

Use of PrEP may be interrupted by the site Investigator due to safety concerns for the participant, use of concomitant medications that could interfere with PrEP or present a safety concern, or if the participant is unable or unwilling to comply with study procedures. All treatment interruptions will be documented.

Concomitant medication use will be recorded. PrEP will be avoided, per investigator discretion, in individuals receiving ongoing therapy with anti-retroviral medications, interferon (alpha, beta, or gamma) or interleukin (e.g., IL-2) therapy, metformin, systemic aminoglycoside antibiotics, amphotericin B, cidofovir, systemic chemotherapeutic agents, other agents with significant nephrotoxic potential, other agents that may inhibit or compete for elimination via active renal tubular secretion (e.g., probenecid).

In addition, for couples in which the HIV-1 infected participant (index) initiates ART, PrEP will be discontinued in the partner (HIV-1 uninfected) participant six months later. By clinician discretion, there may be circumstances in which HIV-1 uninfected partner will continue PrEP use despite >6 months of ART use by the HIV-1 infected partner. These circumstances may include unsuppressed HIV-1 viral load in the HIV-1 infected partner (due to viral load measurement or known poor ART adherence), pregnancy or immediate plans by the couple to conceive a child, or an additional partner of known HIV-1 seropositive status. Any cases of non-discontinuation of PrEP will be documented and reconsideration of PrEP discontinuation will occur at each subsequent visit.

V. SAFETY

The Partners PrEP Study demonstrated that PrEP (including FTC/TDF) was safe for use in heterosexual men and women from Kenya and Uganda. There were no statistically significant differences in the frequency of deaths, serious adverse events, adverse events overall, or key laboratory adverse events (specifically, creatinine elevation and phosphorus decrease) for those receiving PrEP compared to those receiving placebo.

For the purposes of this study, only serious adverse events (SAEs), for both index (HIV-1 infected) and partner (HIV-1 uninfected) participants, and adverse events felt related to PrEP will be documented on case report forms. Serum creatinine will be monitored at Month 1 and then every 6 months; serum creatinine elevation of Grade 1 or greater that is confirmed on a second sample drawn for follow-up of an abnormal result will result in temporary hold of PrEP. A participant with a Grade 2 or higher creatinine or a calculated creatinine clearance of <50 mL/min will have PrEP temporarily withheld, and a second sample will be drawn for confirmation; confirmed Grade 2 or higher creatinine events or confirmed creatinine clearance <50 mL/min will result in permanent PrEP discontinuation. SAEs felt to be related to PrEP will result in temporary hold of PrEP. In the case of temporary holds, the hold will continue until the event is stabilized or resolved. If the event resolves, PrEP may be reinitiated at the discretion of the Investigator, resuming safety monitoring. The severity of clinical symptoms will be scored using the DAIDS Table for Grading the Severity of Adult and Pediatric AEs [45].

Decisions to hold PrEP due to clinical and/or other laboratory safety reasons, or in the event of overdose, will be at the discretion of site Investigator, in consultation with the Protocol Chair. Reporting on adverse events to relevant IRBs will be according to relevant regulations.

Clinical symptoms will be systematically assessed in a structured medical history administered to partner (HIV-1 uninfected) participants. Clinical side effects of TDF and FTC/TDF that have been reported are primarily gastrointestinal, including nausea, vomiting, and flatulence.

VI. DATA ANALYSIS

Sample size justification

The primary goal of this project is to demonstrate feasibility of delivery of PrEP and ART to higher-risk HIV-1 serodiscordant couples in an open-label (i.e., non-clinical trial) setting. Thus, the principal justification for the sample size is inclusion of a sufficient number of HIV-1 serodiscordant couples to indicate implementation feasibility, in a way that would inform normative guidance and public health recommendations. Importantly, in this way, the justification for the sample size is different than that of a comparative study (e.g., a randomized, placebo-controlled trial) – in the proposed study, the informative evaluation is whether PrEP and ART can be implemented, and for whom these strategies will be most acceptable and used.

A sample size of 1000 couples will provide a robust evaluation of the proposed approach, and the diversity of study sites, with urban and rural locations, in two countries, will permit evaluation of targeted recruitment, PrEP and ART uptake, and adherence to these strategies for diverse populations. Moreover, the proposed sample size will provide substantial, and important, precision for estimating the proportion of persons who take up and adhere to PrEP and ART. For example, if initial uptake of PrEP is 80%, the 95% confidence interval on that estimate will be 77.5-82.5%, providing a tight estimate for policymaking and cost-effectiveness analysis. If uptake or adherence is 50%, the corresponding 95% confidence interval will be 46.5-53.5%.

Finally, the proposed samples size will permit detection of key differences between those who do and do not initiate PrEP/ART or do/do not adhere well to PrEP/ART, with the study having 90% power (at α=0.05) to detect relative risk estimates that range from 1.10 to 1.56 – depending on the prevalence of the exposure correlates (e.g., gender, younger age, fertility desire, etc.) and the frequency of PrEP/ART initiation (Table 10).

Table 10. Risk estimates for correlates of PrEP/ART initiation

	% PrEP/ART initiation among unexposed			
Prevalence of exposure correlate	80%	65%	50%	20%
80%	1.11	1.18	1.25	1.56
65%	1.10	1.15	1.21	1.46
50%	1.09	1.15	1.20	1.44
35%	1.10	1.15	1.21	1.46

Analysis

Participant preferences, uptake and adherence to PrEP and ART, sexual behavior and condom use at enrollment and over follow-up will be described. Categorical variables will be detailed in tables, and continuous measures will be summarized using means and standard deviations or medians and ranges, as appropriate. T-tests will be used to detect differences in mean levels of continuous variables and chi-square tests for dichotomous variables. Multivariable proportional hazards models and generalized estimating equations with dichotomous and continuous outcomes will be used to assess independent correlates of initiation and adherence to antiretroviral-based HIV-1 prevention. Qualitative analyses will identify and describe key themes and explore variation within themes. Descriptive content will be inductively assembled to form explanatory accounts.

Cost-effectiveness analysis

Data from the study will be used to conduct cost-effectiveness analyses to compare costs of offering and delivering PrEP as a bridging strategy to ART, including recruitment costs for targeting higher-risk couples and PrEP delivery and monitoring costs, to the current standard of care in terms of costs for ART delivery without risk targeting. Effectiveness will be assessed through estimated numbers of infections averted. The difference in cost is divided by the difference in effectiveness. Simulation mathematical models will be used to simulate the health outcomes from study data, the Partners PrEP results and the literature to consider clinical outcomes beyond the scope of the observational study. Using the outcomes and costs from the study and effectiveness (health outcomes) estimated by the model, cost-effectiveness will be estimated. The cost-effectiveness analysis will follow WHO guidelines and report summary estimates for the PrEP intervention which allow comparison to other strategies to decrease HIV-1 transmission among serodiscordant couples. This approach will help decision makers define priorities and allocate resources. Model outcomes will include 1) HIV-1 incidence, 2) HIV-1-related deaths and 3) HIV-1 related disability adjusted life years (DALYs) averted by antiretroviral-based interventions among highest-risk couples. Cost-effectiveness of antiretroviral-based interventions among high risk couples: costs will be estimated for the intervention and treatment costs incurred (and averted) as a result of the intervention incremental to existing HIV testing and HIV care costs (current practice). Using the effectiveness estimated above and the incremental intervention costs (and savings) of the intervention, the incremental cost-effectiveness ratio (ICER) per HIV related infection, death and DALY averted will be estimated. Results will be reported as the ICER of PrEP compared to current practice per incident HIV-1 case, HIV related death and DALY averted. Following WHO

guidelines, interventions will be judged to be cost-effective if the ICER is <3 times local GDP and very cost-effective if the ICER is <1 times local GDP per DALY averted. Sensitivity analyses will be conducted to identify the price point at which antiretroviral-based interventions for high risk couples are cost-effective interventions to reduce HIV-1 incidence and HIV-1 related disability and death.

PrEP safety among pregnant women

Maternal and fetal outcomes among women who choose and choose not to use PrEP during pregnancy will be described. Infant growth patterns will be described and compared between infants exposed and unexposed to PrEP in utero using multivariable generalized estimating equations with continuous and dichotomous outcomes. Comparisons will also be made to the completed Partners PrEP Study dataset (which includes women using placebo at the time of pregnancy initiation and women using PrEP) and the Antiretroviral Pregnancy Registry. Independent correlates of PrEP use and high adherence during pregnancy will be assessed with multivariable generalized estimating equations.

VII. HUMAN SUBJECTS CONSIDERATIONS

The study protocol, site-specific informed consent forms, participant education and recruitment materials, and other requested documents — and any subsequent modifications — will be reviewed and approved by the IRBs/ECs responsible for oversight of research conducted at the study sites. Subsequent to initial review and approval, the responsible IRBs/ECs will review the study at least annually.

Study oversight

This study will be subject to oversight by an independent data monitoring committee that will periodically review data from the study, including study execution, adherence, HIV-1 incidence, HIV-1 drug resistance, and serious adverse events. Review will be in an unblinded fashion, consistent with the open-label, non-randomized, unblinded nature of the study. The members of the Partners PrEP Study Data and Safety Monitoring Board, who monitored that related clinical trial, will serve as the independent data monitoring committee for the Partners Demonstration Project. The independent data monitoring committee will provide recommendations to the study team as part of six-monthly reviews. Reports from all reviews will be provided for submission to overseeing IRBs/ECs.

Informed consent

Written informed consent will be obtained from each study participant prior to both screening and enrollment. Separate informed consent will be obtained to offer enhanced follow-up for HIV-1 uninfected women using PrEP at the time of pregnancy, including a decision to use or not use in pregnancy and to consent to follow-up of infants to measure growth, using a follow-up schedule similar to that in the Partners PrEP Study. Participants will be offered copies of the informed consent forms.

Each study site is responsible for developing study informed consent forms for local use that describe the purpose of screening and of the study, the procedures to be followed, and the risks and benefits of participation, in accordance with all applicable regulations, based on the samples provided in the Appendix. Each site also is responsible for translating the forms into

local languages and verifying the accuracy of the translation by performing an independent back-translation, which will be reviewed and approved by the Coordinating Center.

Risks

Participants may experience discomfort or pain when undergoing phlebotomy. They also may feel dizzy or faint, and/or develop a bruise, swelling, or infection where the needle is inserted.

Participants may become embarrassed, worried, or anxious when completing their HIV-1 risk assessment and/or receiving HIV-1 counseling. They also may become worried or anxious while waiting for their (or their partner's) HIV-1 test results. Couples-based counseling and discussions of study participation may raise issues between partners, particularly related to blame (from the HIV-1 uninfected partner) and potential termination of the partnership. Participants who learn that they have HIV-1 may experience anxiety or depression related to their test results. At all study sites, individual and couples-based HIV-1 counseling will be provided by counselors and clinicians who have been trained in specific issues related to HIV-1 serodiscordant couples, including stigma, blame, methods to avoid transmission, and available support services.

Although study sites will make every effort to protect participant privacy and confidentiality, it is possible that participants' involvement in the study could become known to others, and that social harms may result (i.e., because participants could become known as participating in a trial involving HIV-1 infected persons). For example, participants could be treated unfairly or discriminated against, or could have problems being accepted by their families and/or communities.

Risks and side effects related to PrEP include: occurring in a minority of individuals taking PrEP - gastrointestinal intolerance, such as nausea, diarrhea or vomiting, flatulence; rare but serious - lactic acidosis/ severe hepatomegaly with steatosis, renal impairment, including cases of acute renal failure and Fanconi's syndrome (renal tubular injury with severe hypophosphatemia), increase in bone metabolism leading to osteopenia, hypersensitivity reaction.

Benefits

HIV-1 uninfected participants will benefit by having free access to PrEP during the study period, until 6 months after their partner initiates ART. HIV-1 infected participants will benefit from monitoring of clinical status and provision or directed referral for ART. Couples may benefit from ongoing access to prevention services. There may be no other direct benefits to participants in this study. However, participants and others also may benefit in the future from information learned from this study.

All couples will be provided with HIV-1 prevention services, including condoms, STI treatment, and ongoing support, including counseling and referral to other support services.

The primary aim of this study is to assess adherence to PrEP and ART among HIV-1 serodiscordant couples who are receiving standard-of-care HIV-1 prevention services. HIV-1 prevention practices, according to national guidelines, will be provided to all couples enrolled in this study. This will include risk reduction counseling, treatment of sexually transmitted infections (STIs), condoms, and referral for medical male circumcision. If prevention strategies are modified during the course of study conduct (i.e. ART is indicated for HIV-1 prevention at

CD4 counts >350 cells/μL), participants will be counseled and referred for these strategies as well.

This study will additionally assess the safety of PrEP among pregnant women. PrEP is an appealing HIV-1 risk reduction strategy for HIV-1 uninfected high risk pregnant women and additional data are needed to inform public health practice regarding continued PrEP use during pregnancy. Women who participate in more frequent study visits during pregnancy may benefit from the information learned from this study regarding PrEP use during pregnancy.

Care for persons identified as HIV-1 infected

This study will identify persons who are infected with HIV-1, either as part of the study screening process or during follow-up of enrolled participants. Study staff will provide participants with their HIV-1 test results in the context of post-test counseling. Persons identified as HIV-1 infected during the study screening process, but who do not meet eligibility criteria or who do not wish to enroll in the study, will be referred to local HIV-1 care services and/or other agencies that provide care or access to treatment.

HIV-1 infected persons who enroll in the study will be referred for HIV-1 clinical care, including primary care and antiretroviral therapy, according to national guidelines. During the course of the study, it is possible that guidelines will change regarding clinical care of persons with HIV-1 (including timing of initiation of ART, optimal therapy, prophylaxis, etc.). At all times during the study, treatment and referral practices for HIV-1 infected persons in the study will adhere to national guidelines for HIV-1 infected persons. After the study ends, HIV-1 infected participants will be provided referrals to other care programs for ongoing HIV-1 primary care. This type of care will also be offered to initially HIV-1 uninfected study participants who become infected during follow-up. The results of batched HIV-1 resistance assays for HIV-1 seroconverters will be made available to the research sites to be considered in ART regimen selection when seroconverters meet national guidelines for ART initiation.

For participants who are HIV-1 infected and who also become pregnant during follow-up, every effort will be made to facilitate access to programs for preventing mother-to-child HIV-1 transmission for appropriate antiretroviral treatment to reduce the probability of HIV-1 transmission from mother to child.

Benefits to the community

An important goal of this study is to achieve the study objectives in a way that provides benefits to the community that endure beyond the proposed study lifetime regardless of the specific outcome of the study. Some of these community benefits are listed below:

<u>Development of couples HIV-1 counseling and testing (CHCT)</u>: Infrastructure to effectively counsel and test couples for HIV-1 will be needed for this study to effectively recruit serodiscordant couples. CHCT capacity developed at participating sites in collaboration with existing local VCT programs through training of local counselors will be a beneficial resource to the community well after the study is completed. Given recent data that 70% of incident HIV-1 cases are transmitted from regular partners, creating awareness, increasing demand and constructing facilities for couples VCT should reduce transmission of HIV-1 among couples. This will have consequent benefits to the family and community by maintaining one healthy partner.

Development of comprehensive HIV-1 prevention centers within a real world setting: This study aims to provide HIV-1 prevention policy makers with information on how to best implement antiretroviral-based HIV-1 prevention. In addition to the provision of this biomedical method, the study site will provide CHCT, and routine adherence counseling. The outcome of the study will be evidence upon which to based policy guidelines for scaling up HIV-1 prevention centers in Kenya, Uganda and nearby countries with similar HIV-1 prevention needs. The HIV-1 prevention centers that serve as sites for this study will be models upon which future centers can be based. Summary outcomes from this study will be submitted to overseeing regulatory bodies and will be especially important for the development of normative guidance.

Sustainable infrastructure for future HIV-1 prevention and treatment trials: A sustainable infrastructure allowing high quality behavioral and laboratory research with HIV-1 serodiscordant couples is needed to study vaccines and other preventive and treatment strategies for HIV-1.

Treatment for injury

Participants will be asked to inform the study staff if they feel they have been injured because of taking part in the study. Injuries may also be identified during laboratory testing, medical histories, and physical examinations. Treatment for adverse events related to study participation will be provided by the study clinic. If treatment is required that is beyond the capacity of the study clinic, the study doctors will refer the participant to appropriate services or organizations that can provide care for the injury.

Study records

Site Investigators will maintain, and store in a secure manner, complete, accurate, and current study records throughout the study. The investigator will retain all study records for at least five years after completion of the study. Study records include administrative documentation and regulatory documentation as well as documentation related to each participant screened and/or enrolled in the study, including informed consent forms, locator forms, case report forms, notations of all contacts with the participant, and all other source documents. Logs linking participant name to study identification number and other identifying information in study files will be retained for five years after the study is completed; after that time, the link will be destroyed. Participants may consent to storage of samples for future research; any future research must be approved by overseeing Institutional Review Boards.

Confidentiality

Every effort will be made to protect participant privacy and confidentiality to the extent possible. Personal identifying information will be retained at the local study site and not forwarded to the University of Washington Coordinating Center. Each study site will establish a standard operating procedure for confidentiality protection that reflects the local study implementation plan and the input of study staff and community representatives to identify potential confidentiality issues and strategies to address them. In addition to local considerations, the protections described below will be implemented at all sites.

All study-related information will be stored securely at the study site. All participant information will be stored in areas with limited access. Data collection, administrative forms, laboratory specimens, and other reports will be identified only by a coded number to maintain participant confidentiality. All records that contain names or other personal identifiers, such as locator

forms and informed consent forms, will be stored separately from study records identified by code number. All local databases will be secured with password-protected access systems. Forms, lists, logbooks, appointment books, and any other listings that link participant ID numbers to other identifying information will be stored in a separate, locked file in an area with limited access.

Participants' study information will not be released without their written permission, except as necessary for oversight by:

- The Protocol Chair or designees
- Study funders
- Site IRBs/ECs
- University of Washington
- Any additional study sponsors

REFERENCES

1. Baeten JM, Celum C, Partners PrEP Study Team. Antiretroviral pre-exposure prophylaxis for HIV-1 prevention among heterosexual African men and women: the Partners PrEP Study. In: *6th IAS Conference on HIV Pathogenesis, Treatment and Prevention*. Rome, Italy; 2011. Abstract MOAX0106.

2. UNAIDS/WHO. *AIDS Epidemic Update 2009*: Available at: http://www.unaids.org/en/KnowledgeCentre/HIVData/EpiUpdate/EpiUpdArchive/2009/default.asp Accessed 4 April 2010; 2009.

3. Merson M, Padian N, Coates TJ, Gupta GR, Bertozzi SM, Piot P*, et al.* Combination HIV prevention. *Lancet* 2008,**372**:1805-1806.

4. Cohen MS, Gay C, Kashuba AD, Blower S, Paxton L. Narrative review: antiretroviral therapy to prevent the sexual transmission of HIV-1. *Ann Intern Med* 2007,**146**:591-601.

5. Karim SS, Karim QA. Antiretroviral prophylaxis: a defining moment in HIV control. *Lancet* 2011,**17**:17.

6. Padian NS, McCoy SI, Karim SS, Hasen N, Kim J, Bartos M*, et al.* HIV prevention transformed: the new prevention research agenda. *Lancet* 2011,**378**:269-278.

7. Quinn TC, Wawer MJ, Sewankambo N, Serwadda D, Li C, Wabwire-Mangen F*, et al.* Viral load and heterosexual transmission of human immunodeficiency virus type 1. Rakai Project Study Group. *N Engl J Med* 2000,**342**:921-929.

8. Lingappa JR, Baeten JM, Wald A, Hughes JP, Thomas KK, Mujugira A*, et al.* Daily aciclovir for HIV-1 disease progression in people dually infected with HIV-1 and herpes simplex virus type 2: a randomised placebo-controlled trial. *Lancet* 2010,**375**:824-833.

9. Baeten J, Kahle E, Lingappa JR, Coombs R, Donnell D, Wald A*, et al.* Genital HIV-1 RNA levels predict risk of heterosexual HIV-1 transmission. *Sci Trans Med* 2011,**3**:77ra29.

10. Ellerbrock TV, Chiasson MA, Bush TJ, Sun XW, Sawo D, Brudney K*, et al.* Incidence of cervical squamous intraepithelial lesions in HIV-infected women. *J Am Med Assoc* 2000,**283**:1031-1037.

11. Phillips AN, Staszewski S, Weber R, Kirk O, Francioli P, Miller V*, et al.* HIV viral load response to antiretroviral therapy according to the baseline CD4 cell count and viral load. *J Am Med Assoc* 2001,**286**:2560-2567.

12. Graham SM, Holte SE, Peshu NM, Richardson BA, Panteleeff DD, Jaoko WG*, et al.* Initiation of antiretroviral therapy leads to a rapid decline in cervical and vaginal HIV-1 shedding. *AIDS* 2007,**21**:501-507.

13. Gupta P, Mellors J, Kingsley L, Riddler S, Singh MK, Schreiber S*, et al.* High viral load in semen of human immunodeficiency virus type 1-infected men at all stages of disease

and its reduction by therapy with protease and nonnucleoside reverse transcriptase inhibitors. *J Virol* 1997,**71**:6271-6275.

14. Marcelin AG, Tubiana R, Lambert-Niclot S, Lefebvre G, Dominguez S, Bonmarchand M*, et al.* Detection of HIV-1 RNA in seminal plasma samples from treated patients with undetectable HIV-1 RNA in blood plasma. *AIDS* 2008,**22**:1677-1679.

15. Cu-Uvin S, Caliendo AM. Genital tract HIV-1 RNA shedding among women with below detectable plasma viral load. *AIDS* 2011,**25**:880-881.

16. Vernazza PL. Genital shedding of HIV-1 despite successful antiretroviral therapy. *Lancet* 2001,**358**:1564.

17. Mofenson LM. Can perinatal HIV infection be eliminated in the United States? *J Am Med Assoc* 1999,**282**:577-579.

18. Wood E, Kerr T, Montaner JS. HIV treatment, injection drug use, and illicit drug policies. *Lancet* 2007,**370**:8-10.

19. Attia S, Egger M, Muller M, Zwahlen M, Low N. Sexual transmission of HIV according to viral load and antiretroviral therapy: systematic review and meta-analysis. *AIDS* 2009,**23**:1397-1404.

20. Donnell D, Baeten JM, Kiarie J, Thomas KK, Stevens W, Cohen CR*, et al.* Heterosexual HIV-1 transmission after initiation of antiretroviral therapy: a prospective cohort analysis. *Lancet* 2010,**375**:2092-2098.

21. Cohen MS, Chen YQ, McCauley M, Gamble T, Hosseinipour MC, Kumarasamy N*, et al.* Prevention of HIV-1 infection with early antiretroviral therapy. *N Engl J Med* 2011,**365**:493-505.

22. Granich RM, Gilks CF, Dye C, De Cock KM, Williams BG. Universal voluntary HIV testing with immediate antiretroviral therapy as a strategy for elimination of HIV transmission: a mathematical model. *Lancet* 2009,**373**:48-57.

23. Rapid advice: antiretroviral therapy for HIV infection in adults and adolescents. In: World Health Organization; 2009.

24. Ware NC, Idoko J, Kaaya S, Biraro IA, Wyatt MA, Agbaji O*, et al.* Explaining adherence success in sub-Saharan Africa: an ethnographic study. *PLoS Med* 2009,**6**:e11.

25. Connor EM, Sperling RS, Gelber R, Kiselev P, Scott G, O'Sullivan MJ*, et al.* Reduction of maternal-infant transmission of human immunodeficiency virus type 1 with zidovudine treatment. Pediatric AIDS Clinical Trials Group Protocol 076 Study Group. *N Engl J Med* 1994,**331**:1173-1180.

26. Chasela CS, Hudgens MG, Jamieson DJ, Kayira D, Hosseinipour MC, Kourtis AP*, et al.* Maternal or infant antiretroviral drugs to reduce HIV-1 transmission. *N Engl J Med* 2010,**362**:2271-2281.

27. Mofenson LM. Protecting the next generation--eliminating perinatal HIV-1 infection. *N Engl J Med* 2010,**362**:2316-2318.

28. Garcia-Lerma JG, Paxton L, Kilmarx PH, Heneine W. Oral pre-exposure prophylaxis for HIV prevention. *Trends Pharmacol Sci* 2010,**31**:74-81.

29. Parikh UM, Dobard C, Sharma S, Cong ME, Jia H, Martin A*, et al.* Complete protection from repeated vaginal simian-human immunodeficiency virus exposures in macaques by a topical gel containing tenofovir alone or with emtricitabine. *J Virol* 2009,**83**:10358-10365.

30. Garcia-Lerma JG, Otten RA, Qari SH, Jackson E, Cong ME, Masciotra S*, et al.* Prevention of rectal SHIV transmission in macaques by daily or intermittent prophylaxis with emtricitabine and tenofovir. *PLoS Med* 2008,**5**:e28.

31. Subbarao S, Otten RA, Ramos A, Kim C, Jackson E, Monsour M*, et al.* Chemoprophylaxis with tenofovir disoproxil fumarate provided partial protection against infection with simian human immunodeficiency virus in macaques given multiple virus challenges. *J Infect Dis* 2006,**194**:904-911.

32. Cong ME, Youngpairoj AS, Zheng Q, Aung W, Mitchell J, Sweeney E*, et al.* Protection against rectal transmission of an emtricitabine-resistant simian/human immunodeficiency virus SHIV162p3M184V mutant by intermittent prophylaxis with Truvada. *J Virol* 2011,**85**:7933-7936.

33. Cohen J. AIDS research. Complexity surrounds HIV prevention advances. *Science* 2011,**333**:393.

34. Abdool Karim Q, Abdool Karim SS, Frohlich JA, Grobler AC, Baxter C, Mansoor LE*, et al.* Effectiveness and safety of tenofovir gel, an antiretroviral microbicide, for the prevention of HIV infection in women. *Science* 2010,**329**:1168-1174.

35. Grant RM, Lama JR, Anderson PL, McMahan V, Liu AY, Vargas L*, et al.* Preexposure chemoprophylaxis for HIV prevention in men who have sex with men. *N Engl J Med* 2010,**363**:2587-2599.

36. Thigpen MC, Kebaabetswe PM, Smith DK, Segolodi TM, Soud FA, Chillag K*, et al.* Daily oral antiretroviral use for the prevention of HIV infection in heterosexually active young adults in Botswana: results from the TDF2 study. In: *6th IAS Conference on HIV Pathogenesis, Treatment and Prevention*. Rome, Italy; 2011. Abstract WELBC01.

37. FHI statement on the FEM-PrEP HIV prevention study. In; 2011.

38. NIH modifies 'VOICE' HIV prevention study in women: oral tenofovir discontinued in clinical trial. In; 2011.

39. Kashuba AD, Patterson KB, Dumond JB, Cohen MS. Pre-exposure prophylaxis for HIV prevention: how to predict success. *Lancet* 2011,**6**:6.

40. Dunkle KL, Stephenson R, Karita E, Chomba E, Kayitenkore K, Vwalika C*, et al.* New heterosexually transmitted HIV infections in married or cohabitating couples in urban

Zambia and Rwanda: an analysis of survey and clinical data. *Lancet* 2008,**371**:2183-2191.

41. Grabbe KL, Bunnell R. Reframing HIV prevention in sub-Saharan Africa using couple-centered approaches. *JAMA* 2010,**304**:346-347.

42. Piot P, Bartos M, Larson H, Zewdie D, Mane P. Coming to terms with complexity: a call to action for HIV prevention. *Lancet* 2008,**372**:845-859.

43. Baeten JM, Celum C, Partners PrEP Study Team. Antiretroviral pre-exposure prophylaxis for HIV-1 prevention among heterosexual African men and women: the Partners PrEP Study In: *6th IAS Conference on HIV Pathogenesis, Treatment and Prevention*. Rome, Italy; 17-20 July 2011.

44. Hallett TB, Baeten JM, Heffron R, Barnabas R, de Bruyn G, Cremin I, *et al.* Optimal uses of antiretrovirals for prevention in HIV-1 serodiscordant heterosexual couples in South Africa: a modelling study. *PLoS Med* 2011,**8**:e1001123.

45. Division of AIDS. Table for grading the severity of adult and pediatric adverse events. In; December 2004.

Appendices 1-4 Sample Study Consent Forms

Each study site is responsible for developing study informed consent forms for local use that describe the purpose of screening and of the study, the procedures to be followed, and the risks and benefits of participation, in accordance with all applicable regulations, based on the samples provided in the following appendices. Each site also is responsible for translating the forms into local languages and verifying the accuracy of the translation by performing an independent back-translation, which will be reviewed by the Coordinating Center before submission to the local IRB.

Appendix 1. TEMPLATE CONSENT: Human Immunodeficiency Virus (HIV) Antibody Test

An open-label, pilot demonstration and evaluation project of antiretroviral-based HIV-1 prevention among high-risk HIV-1 serodiscordant African couples

Version 3.0
10 December 2012

PRINCIPAL INVESTIGATOR: *[insert name]*
[insert physical address and relevant contact info]

INVESTIGATOR'S STATEMENT

Purpose and Benefits

A virus called HIV (Human Immunodeficiency Virus) causes AIDS (Acquired Immunodeficiency Syndrome). Any person with HIV can spread it to others through unprotected sex, needle sharing, or donating blood or other tissues. Infected mothers can spread HIV to their newborns. The test for HIV detects the body's reaction to the virus (antibody). It does not detect the virus itself. You are not required to have the test. Testing for HIV is voluntary. This test is being done for a research study. You should be tested only if you are well informed about the risks and benefits of testing. Please read this consent form carefully so that you can make an informed decision about having the blood test.

What the Test Means

If you test POSITIVE, you have the HIV virus. That means you can pass it to others. The test cannot tell how long a person has been infected. A positive test does not mean that you have AIDS, which is the most advanced stage of HIV infection.

If the test is NEGATIVE, you probably do not have the HIV virus. A negative test usually means that a person is not infected with HIV; however, recently infected persons can have a negative test, which becomes positive in three months after infection. This would mean that your body has not yet made antibody to fight the virus.

False results (a negative test in an infected person, or a positive result in an uninfected person) are rare. Indeterminate (unclear) results are also rare. When a test result does not seem to make sense, a repeat test or another kind of blood test is done to find out if the person is infected or not.

Procedures

This is what will happen if you decide to have the test. First, you will meet with a counselor to get more information about the risks and benefits of the test. They will explain the meaning of test results. They will teach you how to reduce the chance of spreading HIV. They will explain the dangers of HIV infection. Then, they will take blood, either from your arm or from your finger, with a sterile needle. They will test your blood for HIV. It will take about 20 minutes to get your test result. You will be told your result on the same day that you give blood and have the test. The study staff will talk with you about the meaning of your result and how you feel about it. Sometimes HIV tests are not clearly positive but also not negative. In that case, we will do more tests until we know the result for sure. If the test result is positive, you will learn how to notify anyone with whom you have sex, and how to get services for yourself.

Risk and Benefits

The needle used to draw blood for the test may cause discomfort. A bruise may form where the needle enters the vein, and if you get a bruise, it usually goes away within a week.

Learning the test results may cause stress, anxiety and depression for people being tested and for their partners. You might be tempted to have unsafe sex if the result is negative. This would increase your risk of becoming infected with HIV. It is possible that you may feel nervous about the information you are going to give us and concerned about any links between this information and your name or identity.

Many people are afraid that their test results will get into the wrong hands. For example someone might see the test results who might tell others that you have HIV, and prejudice and discrimination, such as gossip, may cause problems in keeping your job or the place where you live. We will take precautions to protect your identity and prevent the study results from being used in this fashion. One risk is that your partner may become upset or leave you after hearing that you are HIV-infected. We encourage you to be tested with your partner so that the counselor can provide information and support to both of you, regardless of your results. If you want to be tested without your partner, and you test positive for HIV, we will provide you with support to avoid negative reactions of your partner, if you decide to notify him/her about your HIV infection. There may be other risks and stresses of being tested that we don't know about now.

Otherwise, the benefits of being tested are personal. Test results may help diagnose a medical problem, guide your health care, help you follow strategies to improve your health, and may help you avoid transmitting HIV to other people. If you are worried about AIDS, you might feel better if you have a negative test. Sometimes knowing that the test is positive can relieve stress. You may want to know your test results before you have sex with a new partner. There may be other benefits of testing that we don't know about now.

Confidentiality
Your HIV antibody test result must be held in the strictest confidence, and no identifying information of any kind will be released to any other person or agency without your specific written permission. No publication or public discussion of the testing will contain information that could identify you.

Other Information
We will tell you the results of the test in person. If you test positive, we will encourage you to notify your sexual partners. The investigator or his representative can answer all your questions about this study. If you have any additional questions, you can ask them now, or contact a study representative at the telephone number on this form.
We will give you a copy of this form.

<u>VOLUNTEER'S STATEMENT</u>
The benefits and risk about HIV testing on the preceding page has been explained to me, and I voluntarily agree to participate. I have had an opportunity to ask questions. I have been told that if I have future questions about the research, I can ask one of the investigators listed above. If I have questions about my rights as a research subject, I may call [insert name or title of person on the IRB/EC or other organization appropriate for the site] at [insert telephone number and/or physical address of above].

[Insert signature blocks as required by the local IRB/EC:]

Witness Name (print)	Witness Signature	Date
Participant Name (print)	Participant Signature/Thumbprint	Date
Study Staff Conducting Consent Discussion (print)	Study Staff Signature	Date

Appendix 2. TEMPLATE CONSENT: Screening

An open-label, pilot demonstration and evaluation project of antiretroviral-based HIV-1 prevention among high-risk HIV-1 serodiscordant African couples
Version 3.0
10 December 2012

PRINCIPAL INVESTIGATOR: *[insert name]*
[insert physical address and relevant contact info]

INFORMED CONSENT
We are asking you to volunteer to have screening tests to find out if you are eligible for a research study. The study is for partners between whom one person has HIV and the other person does not have HIV. HIV is the virus that causes AIDS. This study is sponsored by the University of Washington, the Bill and Melinda Gates Foundation, the National Institutes of Health, and the United States Agency for International Development, which are located in the USA.

If you decide to take part in the screening procedures you will be asked to sign this consent form or make your mark in front of a witness. We will give you a copy of this form. This consent form might contain some words that are unfamiliar to you. Please ask us to explain anything you may not understand.

PURPOSE OF THE STUDY
Recent research showed that taking a medication called Truvada® can reduce the chances for HIV-**uninfected** men and women to get the HIV virus from their partner by 75 percent. This is called **pre-exposure prophylaxis**. The study also learned that Truvada® is safe (meaning that it does not produce significant health problems in persons who take them) when taken by HIV-uninfected men and women.

Other recent research showed that HIV-**infected** men and women who took HIV medications (called ART or anti-retroviral therapy) took longer to get sick with AIDS and also lowered the risk of transmitting the virus to their partner by 96%.

This purpose of this study is to find out more about the choices couples in which one partner has HIV and the other does not make to prevent getting HIV; couples in which one partner has HIV and the other does not are called discordant couples. We also want to understand how easy or hard it is to take these medications to prevent and treat HIV and which kinds of medication couples prefer.

Approximately 1000 couples will be enrolled in this study. Up to 500 couples will be enrolled in this study clinic [clinic].

Each couple will be in the study for up to 24 months.

SCREENING
The screening tests for this study will include questions and tests done from samples of blood. Some people may not be able to join the study because of information found during the screening process. You and your partner must both be willing and eligible to participate in the study.

YOUR PARTICIPATION IS VOLUNTARY

Before you learn about the screening tests, it is important that you know the following:

- You do not have to be in this study if you do not want to.

- You may decide not to have the screening tests, or to stop the screening tests at any time, without losing your regular medical care.

- If you decide not to have the screening tests, you can still join other research studies later, if available and you qualify.

- You may be asked about joining other studies. Due to the time commitment from being in this study, you may not be eligible to join this study if you are in other studies. If you do not agree to join these other studies, you may still take part in this study.

- We are only asking you to have the screening tests at this time. Even if you agree to have the screening tests and are eligible to join the study, you do not have to join the research study.

- You will receive the results of the screening tests even if you are not eligible to join the research study.

SCREENING PROCEDURES

Screening procedures will begin today, after you read, discuss, and sign or make your mark on this form. The screening procedures will include the following:

- The study staff will ask you where you live and other questions about you and your sexual practices.

- We will counsel you about HIV and other infections passed during sex, and how to avoid these infections.

- Even if you have recently been tested for HIV, we will need to repeat the HIV test today as part of screening for the study. You will sign a separate form to consent for HIV testing. The study staff will talk with you about the HIV test, what it may mean to know your HIV test results, including issues for partners when one partner is HIV-infected and the other is HIV-uninfected. We will then ask your partner and you whether you are ready to be tested today and receive your HIV test results. You must receive your HIV test results to be in the research study.

- If you are in an HIV discordant couple and you <u>do not</u> have HIV infection:
    - We will ask you questions about your medical history.

    - You will be asked to give us permission to obtain a blood sample for laboratory tests. No more than 21 ml (about 1 ½ tablespoons) will be collected for all the screening tests, including the HIV test. Some of the blood sample will be used to test for hepatitis B infection and some will be used to test the function of your kidneys.

- If you are in an HIV discordant couple and you <u>do</u> have HIV infection:

- We will ask you questions about your medical history. As part of these questions, you will be asked about symptoms of AIDS.

- You will be asked to give us permission to obtain a blood sample for laboratory tests. No more than 21 ml (about 1 ½ tablespoons) will be collected for all the screening tests, including the HIV test. Some of the blood sample will be used for a test called a CD4 cell count. The CD4 count is a measure of how well your immune system is functioning. The lower the CD4 count in persons who are HIV-infected, the more at risk they are for having problems from AIDS. Some of the blood sample will be used to test for the amount of HIV virus in your blood, called 'viral load.'

If both you and your partner are found to be eligible for the research study, the study staff will fully explain the study to you and answer any questions you have. You and your partner must have at least one other visit here at the clinic together to learn about the study. You also may have additional separate visits. If you and your partner decide to take part in the research study, each of you will be asked to sign another consent form.

RISKS AND/OR DISCOMFORTS
You may feel discomfort or pain when your blood is drawn. You may feel dizzy or faint. You may have a bruise where the sterile instrument (needle) goes into your arm.

You may become embarrassed, worried, or anxious when talking about your sexual practices, ways to protect against HIV and other infections passed during sex, and your test results. You may become worried or anxious while waiting for your test results. Having the screening tests, talking about HIV, and finding out your and your partner's results could cause problems between you and your partner. Sometimes partners experience anger and blame when one partner learns that s/he is HIV-positive and the other is HIV-negative ("HIV-discordant"), sometimes leading to the relationship ending. The counseling that you will receive through the study staff will help you understand this situation. Previous studies have shown that counseling is important to help HIV-discordant partners stay together and to reduce the chances of spreading HIV. Trained counselors are available through the study who will help you and your partner deal with any feelings or questions you may have.

The study staff will make every effort to protect your privacy and confidentiality while you are having the screening tests. However, it is possible that others may learn of your participation here and, because of this, may treat you unfairly or discriminate against you. For example, you could have problems getting or keeping a job, or being accepted by your family or community.

BENEFITS
You may or may not get direct benefit from the screening tests. However, you and your partner will get counseling and testing for HIV. You will get information on how to protect against HIV and other infections passed during sex. You will get free condoms. For other health problems that cannot be treated at this clinic, the study staff will tell you about other places you can go for treatment. The study staff will also tell you about other places that provide care and support for people with HIV/AIDS. You may also contribute to understanding about HIV in couples in Africa, which will help others in the future.

REASONS WHY YOU MAY BE WITHDRAWN FROM THE SCREENING PROCESS
You may be removed from the screening process without your consent for the following reasons:

- The research study is stopped or canceled.
- You are not willing to find out your HIV test result.
- You are not willing to talk about HIV and your HIV test result with your partner.
- You or your partner are not able to attend clinic visits or complete the screening tests.
- You or your partner are found to not be eligible for the research study, for any of a number of reasons. We may not be able to tell you why you and your partner are not eligible.
- The study staff feels that having the screening tests would be harmful to you or your partner.

COSTS TO YOU
There is no cost to you for the screening tests.

REIMBURSEMENT
[*Sites to insert information about local incentives*:] [You will receive [*site specified amount*] for your time and effort at each scheduled screening visit. You also will receive payment for the costs of [lost work, travel, and/or childcare] due to your visits.][*Note that reimbursement will be only for research procedures, to try to mimic as real-world delivery of ART and PrEP as possible.]*

CONFIDENTIALITY
Efforts will be made to keep your personal information confidential. However absolute confidentiality cannot be guaranteed. Your personal information may be disclosed if required by law. Any sample from you or information about you will be identified only by code and not by name. The link between your name and code will be kept in a secure location at the clinic only. Any publication of this study will not use your name or identify you personally.
The records of your screening tests may be reviewed by study staff and representatives of:

- the University of Washington, including study monitors
- the Bill and Melinda Gates Foundation
- the United States National Institutes of Health
- [insert applicable local authorities, e.g., Ministry of Health]
- [insert names of applicable IRBs/ECs]

RESEARCH-RELATED INJURY
[Sites to amend this sample text as needed, and/or specify institutional policy:] It is unlikely that you will be injured as a result of having the screening tests. If you are injured as a result of having the screening tests, the study staff will give you immediate necessary treatment for your injuries, free of charge. The study staff also will tell you where you can get additional treatment for your injuries, if needed. There is no program for monetary compensation or other forms of compensation for such injuries. You do not give up any legal rights by signing this consent form.

PROBLEMS OR QUESTIONS
If you ever have any questions about the screening tests, or if you have a research-related injury, you should contact [insert name of the investigator or other study staff] at [insert telephone number and/or physical address].

If you have questions about your rights as a research participant, you should contact [insert name or title of person on the IRB/EC or other organization appropriate for the site] at [insert telephone number and/or physical address of above].

STATEMENT OF CONSENT AND SIGNATURES
I have read this form or had it read to me. I have discussed the information with study staff. My questions have been answered. I understand that my decision whether or not to take part in the screening tests is voluntary. I understand that if I decide to have the screening tests I may withdraw at any time. By signing this form I do not give up any rights that I have as a research participant.

[Insert signature blocks as required by the local IRB/EC:]

______________________	____________________________	_______________
Participant Name (print)	Participant Signature/Thumbprint	Date
______________________	______________________	______________
Study Staff Conducting Consent Discussion (print)	Study Staff Signature	Date
______________________	__________________________	_______________
Witness Name (print)	Witness Signature	Date

Appendix 3. TEMPLATE CONSENT: Enrollment

An open-label, pilot demonstration and evaluation project of antiretroviral-based HIV-1 prevention among high-risk HIV-1 serodiscordant African couples
Version 3.0
10 December 2012

PRINCIPAL INVESTIGATOR: *[insert name]*
[insert physical address and relevant contact info]

INFORMED CONSENT
We are asking you to volunteer to continue in this research study. This study is for partners in which one person has HIV and the other does not. HIV is the virus that causes AIDS. Before you decide whether to take part in the study, we would like to explain the purpose of the study, the risks and benefits, and what would be expected of you if you agree to be in the study. This study is sponsored by the University of Washington, the Bill and Melinda Gates Foundation, the National Institutes of Health, and the United States Agency for International Development, which are located in the USA.

If you decide to participate in the research study, you will be asked to sign this consent form or make your mark in front of a witness. We will give you a copy of this form. This consent form might contain some words that are unfamiliar to you. Please ask us to explain anything you may not understand.

PURPOSE OF THE STUDY
Recent research showed that taking a medication called Truvada® can reduce the chances for HIV-**uninfected** men and women to get the HIV virus from their partner by 75 percent. In research studies, people who took the Truvada® medication consistently reduced their chances of getting HIV by as much as 90 percent. When HIV-uninfected people take medication to prevent getting the HIV virus it is called **pre-exposure prophylaxis**. Research studies have also learned that Truvada® is safe (meaning that they do not produce significant health problems in persons who take them) when taken by HIV-uninfected men and women.

Other recent research showed that HIV-**infected** men and women who took HIV medications (called ART or antiretroviral therapy) took longer to get sick with AIDS and also lowered the risk of transmitting the virus to their partner by 96%.

This purpose of this study is to find out more about the choices couples in which one partner has HIV and the other does not make to treat or prevent HIV; couples in which one partner has HIV and the other does not are called discordant couples. We also want to understand how easy or hard it is to take medications to prevent and treat HIV and which kinds of medication couples prefer.

Approximately 1000 couples will be enrolled in this study. Up to 500 couples will be enrolled at [site clinic].

YOUR PARTICIPATION IS VOLUNTARY
This consent form gives information about the study that we will discuss with you. Once you understand the study, and if you agree to take part, we will ask you to sign your name or make your mark on this form. We will offer you a copy to keep.

Before you learn about the study procedures, it is important that you know the following:

- You do not have to be in this study if you do not want to join.
- You may decide not to take part in this study, or to withdraw from the study at any time, without losing the benefits of your or your partner's routine medical care.
- If you decide not to take part in this study, you can still join another research study later, if one is available and you qualify.

If you decide to take part in this study, after your enrollment visit you will be in the study up to 24 months.

STUDY APPROACH

In this research study, HIV uninfected partners will be offered Truvada® to be taken as one pill once each day, as pre-exposure prophylaxis to prevent HIV infection. The Truvada® medication will be provided by this clinic, free of charge. Truvada® is a combination of two medications: emtricitabine at 200 mg once per day and tenofovir at 300 mg once per day. Truvada® was chosen for this study because it has low levels of side effects, it can be taken once per day, HIV does not easily become resistant to Truvada®, and recent studies have shown that Truvada® reduces the risk of getting HIV. Gilead Sciences is the pharmaceutical company that produces Truvada®, and Gilead Sciences will supply the medications for this study but is not otherwise involved in the study.

HIV infected partners will be assessed for needing HIV medications for treatment, by measuring the CD4 count (a type of blood cell that helps fight infection). If the HIV-infected partner's CD4 count falls low enough to get HIV medication, then the HIV infected partner will be provided HIV treatment medications here at this clinic or referred to an HIV care center to receive HIV treatment medications.

When the HIV infected partner starts HIV medications, then six months later, the HIV-uninfected partner will stop taking Truvada®. If the HIV-infected partner decides not to take ART, then the study team will continue to provide Truvada® medication for the HIV-uninfected partner. In this way, you and your partner are able to both use medications to prevent HIV, depending on your own preferences and the CD4 count level of the infected partner. You and your partner's study visits will stay the same, no matter what choices you make. All couples will get condoms and counseling on how to avoid HIV and other infections passed during sex.

STUDY PROCEDURES

If you decide to take part in the study, your first visit will continue today after you read, discuss, and sign or make your mark on this form. At today's visit, several things will happen:

<u>Both partners</u>:

- Be asked questions about your health and medical history, including whether you have any clinical symptoms, your sexual practices and other behaviors, and your feelings about taking medications for HIV prevention.
- You will undergo a physical exam.

HIV uninfected partners:

- We will ask your permission to obtain a blood sample [up to 21 ml / about 1 ½ tablespoons]. The blood sample will be used for tests at the clinic and by study researchers at the University of Washington. Some of the blood will be used to test for HIV today; if the HIV test is positive today, you will not be able to enroll in this study.

- Women will be asked to provide a urine sample for a pregnancy test. You are not eligible for this study if you are pregnant.

- If you are willing to take Truvada®, you will receive one bottle of pills, enough to last one month. You should take one pill once every day, by mouth. The study staff will counsel you on methods to not forget to take the pills every day. You will also be given a special bottle lid, called a MEMS cap, that will record each day and time your medication bottle is opened. We will show you how to use this special lid. Please bring this cap and all your bottles with you to each clinic visit.

HIV infected partners:

- If you are eligible to start HIV medications for treatment today, you will be offered to start those here or referred to a clinic to start them.

After today's visit, both partners will schedule study follow-up visits in one month, two months later, then every three months after that. Both partners will also have regular follow-up visits scheduled on the same days (when possible). When possible, you and your partner should come to the clinic together, for your convenience and so you can have counseling together as a couple. If that is not possible, you can come for your visits by yourself if you wish. Each visit will take about 60 minutes.

At each study visit you will:

Both partners:

- Be asked questions about your health and medical history, including whether you have any clinical symptoms, your sexual practices and other behaviors, and your feelings about taking medications for HIV prevention.

- Talk with study staff about ways to avoid HIV and other infections passed during sex. We encourage you to have this counseling with your partner, but you can have it by yourself if you wish. You will be offered condoms.

- Get medical care or referrals for medical care and other services if you need them.

- Give updated information on where you live and how to keep in contact with you. The study staff will use this information to remind you of scheduled visits. If you miss a visit, the study staff will try to contact you by [*describe site-specific methods*]. They may try to reach you through the contact people that you list. If they talk to these people, they will not tell them why they are trying to reach you.

- Women will be asked whether they think they may be pregnant. A urine pregnancy test may be done.

HIV uninfected partners:

- We will ask your permission to obtain a blood sample [up to 21 ml / about 1 ½ tablespoons]. The blood sample will be used for tests at the clinic and by study researchers at the University of Washington. Some of the blood will be used to test for HIV.

- If your HIV test is negative you can continue to receive Truvada® medication. Each visit, you will return the Truvada® medication bottles, the MEMS cap, and any pills left over. You will be asked to answer questions about the pills you took during the previous month and be counseled about methods for not forgetting to take your pills during the following month. The pharmacy staff will provide you with new bottles with pills to last until your next visit. You will be given your MEMS cap back. Please bring this cap and all your bottles with you to each clinic visit.

- If your partner starts HIV medications for treatment, you will stop receiving the Truvada® medication six months later.

- At the first month after starting Truvada® and then every 6 months, you will have a blood sample to check the safety of Truvada® for your kidneys. If at any time you have an abnormal result, we will contact you so that you will know and schedule you to return for another visit to recheck the result and evaluate you.

<u>HIV infected partners</u>:

- If you started HIV medications for treatment, the study staff will talk to you about how well you take the medication.

- Every 6 months, you will have blood drawn for a CD4 count and for a test to measure the amount of HIV in your blood [up to 21 ml / about 1 ½ tablespoons]. If the results of this test show that you are eligible to receive HIV medications for treatment, you will be offered to start that here or be referred for treatment.

At any time in the study:

- If you or the study staff thinks you may have any health problems, you may need to undergo a physical exam and may need to provide blood or other samples for testing.

- You can have extra counseling and testing for HIV if needed between scheduled visits, either with your partner or by yourself.

- If you and your partner end your relationship before your last scheduled study visit, we will ask you to stay in the study as originally scheduled and continue the study medication.

- If you decide to leave the study before your last scheduled study visit, we will ask you to have a final study visit with all the exams and tests listed above.

Long interviews

At some point during the study, the staff may invite you to answer more questions about your opinions of the different medications that can be used to prevent passing the HIV virus, your future wishes for more children, and your risks for getting or passing on HIV. An interview will

take no more than two hours. Some interviews will be done individually and some will be done as a group session with other people who are participating in the study. We will record the interviews so we can write them down later. This recording will then be destroyed.

PREGNANCY (women only)
During this study, you will receive counseling at each visit about the potential that you may become pregnant. You will also receive counseling about your options for contraception. You can receive some forms of contraception from the study clinic or be referred to an appropriate clinic for contraception. You may choose whether or not you want to receive contraception.

<u>HIV uninfected women</u>:
Although infants born to HIV-infected women taking Truvada® during pregnancy have not been found to have a greater chance of having birth defects, we do not know for sure if these drugs are safe to the fetus in women who become pregnant. For this reason, if you become pregnant, you will continue to be followed in the research clinic. We also will offer you a chance to continue using PrEP and have more frequent study visits during pregnancy and for your baby to have study visits for up to 12 months. You can make this decision at the time when we first know that you are pregnant. You will also receive, or be referred for, antenatal care. If you do not carry the pregnancy successfully to term, you will resume regular study visits and the Truvada® medication once you are no longer pregnant.

<u>HIV infected women:</u>
If you become pregnant at any time during this study you will be referred for antenatal care and HIV care.

IF THE HIV UNINFECTED PARTNER BECOMES HIV INFECTED
During the course of the study we will provide you with condoms, Truvada® and/or HIV treatment, and other materials to help prevent the HIV uninfected partner from getting HIV. However it is possible to become HIV infected.

If the HIV uninfected partner has a positive HIV test during the study:

- The study staff will talk with you about this test result and what this means for you.
- You will stop the taking the Truvada® medication.
- The staff will ask your permission to obtain a second blood sample [up to 95.5 ml / less than 7 tablespoons] that will be used to confirm the initial positive test and to perform a CD4 count, a measure of your immune function, and to measure the amount of HIV in your blood.
- You will undergo a physical exam
- Women will be asked to provide a urine sample for a pregnancy test.
- You will then be asked to return for another visit after about 2 weeks. At that visit, results from the confirmatory HIV test will be available. If those results confirm that you have become infected with HIV, we will ask your permission to obtain another blood sample [up to 95.5 ml / less than 7 tablespoons] and you will be referred to an HIV care clinic.

IMPORTANCE OF NOT SHARING THE STUDY MEDICATION

It is very important that you do not share HIV medications with your partner or with anyone else. Although Truvada® is used to treat HIV infection, it is only effective for treating people who already have HIV infection if they are used in combination with other medications. Thus, Truvada® by itself is only for HIV uninfected people. Similarly, HIV medications for treatment are only for HIV infected people, and must be used every day.

SPECIMEN STORAGE AND USE OF SAMPLES AND DATA FOR FUTURE STUDIES

We would like to save data from this study and samples of your blood at [local site] and at the University of Washington for future research by us and by other researchers. We will use these samples only for research related to HIV, HIV-related diseases, and sexually-transmitted infections. Before your samples leave the clinic, they will be assigned a code and your name will not be on them. Your name will be linked to the code only at this clinic and only for five years after the study is completed. After that time, the link between your name and the code on your data and samples will be destroyed. An Institutional Review Board or Independent Ethics Committee, which watches over the safety and rights of research participants, must approve any future research studies using your data and samples. If you agree to store your samples, we will keep them for as long as sample remains that can be used for future research. If you do not want to have your samples saved for future research, you can still be in this study and your samples will be destroyed once testing for this study is completed. If you agree to store your samples now, but change your mind before the end of the study, let the study staff know and we will make sure that your samples do not get stored for future research. We will not sell your data or samples. Tests done on your samples may lead to a new invention or discovery. We have no plans to share any money or other benefits resulting from this invention or discovery with you.

RISKS AND/OR DISCOMFORTS

You may feel discomfort or pain when your blood is drawn. You may feel dizzy or faint. You may have a bruise where the needle goes into your arm.

You may become embarrassed, worried, or anxious when talking about your sexual practices, ways to protect against HIV and other infections passed during sex, and your HIV test results. You may become worried or anxious while waiting for your test results. If you become infected with HIV, knowing this could make you worried or anxious. Talking about HIV and finding out your test results could cause problems between you and your partner. Trained counselors will help you and your partner deal with any feelings or questions you may have.

The study staff will make every effort to protect your privacy and confidentiality while you are having the study procedures. However, it is possible that others may learn of your participation here and, because of this, may treat you unfairly or discriminate against you. For example, you could have problems getting or keeping a job, or being accepted by your family or community.

FOR HIV UNINFECTED PARTNERS:

Risks potentially related to the Truvada® medication

You may have symptoms or adverse effects while participating in the study. These symptoms or adverse effects may be due to participation in the study or due to illnesses that have no relation to the study, like a cold or flu. All persons who participate in this study will be watched carefully to monitor their health. You should tell the doctor of the study clinic about any symptoms that you feel while you are participating in the study. You will be given a telephone number where

the study doctors will be available 24 hours a day, 7 days a week. You should call them if you experience any serious symptoms.

The adverse effects that can occur in a small proportion of people taking Truvada® are well known because the medication has been used by many people. Some mild adverse effects are expected to occur in up to 1 in 10 persons who take Truvada®. Other adverse effects are more serious, but are expected to occur in less than 1 in 100 persons who take Truvada®. Occasional adverse effects include: mild problems of kidney function that are only detected by laboratory tests; lack of energy/fatigue; upset stomach, vomiting, soft or liquid stools; dizziness. Rare adverse effects include: rash; liver function problems; serious kidney damage; allergic reaction. Small changes in the mineral density of bones were observed in studies of people who were given Truvada®, but the changes in the mineral density of the bones did not cause any fractures, or other problems that bothered the patients. Lactic acidosis has occurred in HIV-infected persons taking Truvada®, in combination with other drugs. Lactic acidosis is a condition that can produce shortness of breath, nausea, and liver failure. This is a serious adverse effect of some medications used for HIV infection. **You should call or come to the study clinic if you have unexplained increased or decreased urination, weight loss, cramps, muscle pain, dizziness, excessive fatigue, nausea, vomiting, or shortness of breath. If you have these symptoms, or any other symptoms that concern you, the study staff will evaluate your symptoms and determine whether you should stop your Truvada® pills.**

Other medications
Many medications can be taken at the same time as the medications used in this research study. However, some medications should not be taken while you are taking the study medication. When you visit the study clinic, we will ask you about other medications that you are taking.

The medications used in this study may have side effects that no one knows about yet. The researchers will let you know if they learn anything that might make you change your mind about continuing to participate in the study.

Risk of acquiring HIV infection and drug resistance
You may become infected with HIV during this study from your HIV-infected partner or from other sexual partners you may have. It is very important to use all the known risk reduction strategies to prevent the acquisition of HIV, like using condoms for all sexual relations and keeping your number of sexual partners low. You could become infected with a strain of the HIV virus that could be resistant to Truvada® or other medications used for HIV treatment. Resistance to antiretroviral medications may make effective HIV treatment more difficult and may limit your treatment options. You will be able to discuss treatment and the generation of resistance to medications with the study doctor.

For more information about risks of this study, ask your study doctor.

BENEFITS
You may or may not get direct benefit from being in this study. You or others may benefit in the future from information learned in this study. You also may get some personal satisfaction from being part of research on HIV.

The HIV uninfected partner will be receiving Truvada®, which is known to help keep HIV-uninfected individuals who have HIV-infected partners from getting HIV. You will get counseling and testing for HIV. You will get free condoms. While Truvada® has been shown in research

studies to decrease the chances of getting HIV, it does not provide 100% protection; using condoms every time you and your partner have sex is important to protect against HIV. If you or your partner have health problems that may be due to infections passed during sex, you will get medicine to treat them, if needed. For other health problems, the study staff will give you care and treatment that is available at the clinic. For care and treatment that is not available at the clinic, study staff will tell you about other places where care and treatment may be available.

The HIV infected partner will receive care related to his/her HIV infection while this study is ongoing. This care will be either be provided at this study clinic or through a referral clinic. Medications used to treat HIV will be given per national guidelines.

If the HIV uninfected partner becomes infected with HIV while in this study, you will be offered counseling and referred to HIV care.

After the study is over, the study staff will refer you and your partner to other HIV care programs that are available to you.

NEW FINDINGS
You will be told any new information learned during this study that is important for your health or might cause you to change your mind about staying in the study. You will be told when the results of the study may be available, and how to learn about them.

COSTS TO YOU
There is no cost to you for being in this study. Treatments available to you from the study will be given free of charge.

REASONS WHY YOU MAY BE WITHDRAWN FROM THE STUDY
You may be removed from the study without your consent for the following reasons:

• The study is stopped or canceled.

• The study staff feel that staying in the study would be harmful to you.

ALTERNATIVES TO PARTICIPATION
[*Sites to include/amend the following if applicable:* There may be other studies going on here or in the community that you or your partner may be eligible for. If you wish, we will tell you about other studies that we know about. There also may be other places where you can go for HIV counseling and testing. We will tell you about those places if you wish.]

REIMBURSEMENT
[*Sites to insert information about local incentives*:] [You will receive [*site specified amount*] for your time and effort at each scheduled visit. You also will receive payment for the costs of [lost work, travel, and/or childcare] due to your visits.][*Note that reimbursement will be only for research procedures, to try to mimic as real-world delivery of ART and PrEP as possible.]*

CONFIDENTIALITY
Efforts will be made to keep your personal information confidential. However, absolute confidentiality cannot be guaranteed. Your personal information may be disclosed if required by law. Any sample from you or information about you will be identified only by code. The link between your name and code will be kept in a secure location at the clinic only. We will not discuss any information about you with your partner unless you give written permission, and we

will encourage you to be present during the discussion. Any publication of this study will not use your name or identify you personally.

Your study records may be reviewed by study staff and representatives of:

- the University of Washington, including study monitors
- the Bill and Melinda Gates Foundation
- The United States National Institutes of Health
- The United States Agency for International Development
- [insert applicable local authorities, e.g., Ministry of Health]
- [insert names of applicable IRBs/ECs]

RESEARCH-RELATED INJURY
[Sites to amend this sample text as needed, and/or specify institutional policy]
The study staff will monitor your health and the health of your partner while you are in this study. If you or your partner has any health problems between visits, please contact the study staff. If you have a medical emergency that requires immediate care, [insert site-specific instructions].

If you are injured from participating in this study, you will be offered care at the study clinic, free of charge. It is important that you tell the members of the team of researchers at this clinic if you feel that you have been injured because of taking part in this study. There is not a program of monetary compensation through this institution. If you require medical care that the study clinic cannot provide, the study doctors will refer you to the appropriate services or organizations that can provide care for the injury. You do not give up any legal rights by signing this consent form.

PROBLEMS OR QUESTIONS
If you ever have any questions about this study, or if you have a research-related injury, you should contact [insert name of the investigator or other study staff] at [insert telephone number and/or physical address].

If you have questions about your rights as a research participant, you should contact [insert name or title of person on the IRB/EC or other organization appropriate for the site] at [insert telephone number and/or physical address of above].

STATEMENT OF CONSENT AND SIGNATURES
I have read this form or had it read to me. I have discussed the information with study staff. My questions have been answered. I understand that my decision whether or not to take part in the study is voluntary. I understand that if I decide to join the study I may withdraw at any time. By signing this form I do not give up any rights that I have as a research participant.

[Insert signature blocks as required by the local IRB/EC:]

______________________	___________________________	_______________
Participant Name (print)	Participant Signature/Thumbprint	Date
______________________	___________________________	_______________

Study Staff Conducting Study Consent Discussion (print) | Staff Signature | Date

____________________ ________________________ ______________

Witness Name (print) | Witness Signature | Date

SPECIMEN STORAGE AND USE OF YOUR DATA AND SAMPLES FOR FUTURE STUDIES:

Please initial and date one option:

___________ I DO agree to store my data and samples for future research into HIV, HIV-related diseases, and other sexually transmitted diseases.

____________ I DO NOT agree to store my data and samples for future research into HIV, HIV-related diseases, and other sexually transmitted diseases.

[Insert signature blocks as required by the local IRB/EC:]

____________________ ________________________ ______________

Participant Name (print) | Participant Signature/Thumbprint | Date

____________________ ________________________ ______________

Study Staff Conducting Study Consent Discussion (print) | Staff Signature | Date

____________________ ________________________ ______________

Witness Name (print) | Witness Signature | Date

Appendix 4. TEMPLATE CONSENT: Additional follow up visits for HIV-1 uninfected pregnant women

An open-label, pilot demonstration and evaluation project of antiretroviral-based HIV-1 prevention among high-risk HIV-1 serodiscordant African couples
Version 3.0
10 December 2012

PRINCIPAL INVESTIGATOR: *[insert name]*
[insert physical address and relevant contact info]

INFORMED CONSENT
We are asking you to volunteer to have extra visits as part of this research study. These extra visits are for pregnant women without HIV who were using Truvada® when they became pregnant. Before you decide if you want to have these extra visits, we would like to explain the purpose, the risks and benefits, and what would be expected of you if you agree to be in the study.

If you agree to have these extra visits, we will ask you to sign this consent form or make your mark in front of a witness. We will give you a copy of this form. This consent form might contain some words that are unfamiliar to you. Please ask us to explain anything you may not understand.

PURPOSE OF THE STUDY
Research studies have shown that taking a medication called Truvada® can lower the chances for HIV-**uninfected** men and women to get the HIV virus from their partner by 75 percent. In those research studies, those people who took the Truvada® medication every day reduced their chances of getting HIV by as much as 90 percent. When HIV-uninfected people take medication to prevent getting the HIV virus it is called **pre-exposure prophylaxis**. Research studies have also learned that Truvada® is safe and does not lead to significant health problems in persons who take the medication.

Other research studies have found that pregnant women who take the Truvada® medication do not have increased risks of serious problems with their pregnancy and their babies do not have serious birth defects. More research studies HIV-uninfected women taking Truvada® medication during pregnancy are needed.

This purpose of the extra visits in this study is to find out more about using Truvada® during pregnancy. Specifically, this research will study the effects on the growth and development of infants born to women using Truvada® and the occurrence of side effects in women using Truvada® during pregnancy.

YOUR PARTICIPATION IS VOLUNTARY
This consent form gives information about extra visits in the study that we will discuss with you. Once you understand the study, and if you agree to take part, we will ask you to sign your name or make your mark on this form. We will offer you a copy to keep.

Before you learn about the study procedures, it is important that you know the following:

- You do not have to participate in the additional study visits if you do not want to.

- You may decide not to take part in these extra visits, or to withdraw from the study at any time, without losing the benefits of your or your partner's routine medical care and without losing the opportunity to continue with the regular visits in the primary study you have consented to.

- If you decide not to have these extra visits, you can still join another research study later, if one is available and you qualify.

If you decide to have these extra visits, you will have extra visits for as at least long as you are pregnant and your baby will be in this study for up to 12 months.

STUDY PROCEDURES
If you decide to have these extra visits, you will continue to have study visits as you were having but now they will be every month instead of every 3 months.

At each study visit you will:

- Be asked questions about your health and medical history, including whether you have been sick, your sexual practices and other behaviors, and your feelings about taking medication for HIV prevention.

- Talk with study staff about ways to avoid HIV and other infections passed during sex. We encourage you to have this counseling with your partner, but you can have it by yourself if you wish. You will be offered condoms.

- Get medical care or referrals for medical care and other services if you need them.

- Give updated information on where you live and how to keep in contact with you. The study staff will use this information to remind you of scheduled visits. If you miss a visit, the study staff will try to contact you by [*describe site-specific methods*]. They may try to reach you through the contact people that you list. If they talk to these people, they will not tell them why they are trying to reach you.

- We will ask your permission to obtain a blood sample [up to 21 ml / about 1 ½ tablespoons]. The blood sample will be used for tests at the clinic and by study researchers at the University of Washington. Some of the blood will be used to test for HIV.

- If your HIV test is negative you can continue to receive Truvada® medication. Each visit, you will return the Truvada® medication bottles, the MEMS cap, and any pills left over. You will be asked questions about the pills you took during the past month and be counseled about methods for remembering to take your pills during the next month. The pharmacy staff will provide you with new bottles with pills to last until your next visit. You will be given your MEMS cap back. Please bring this cap and all your bottles with you to each clinic visit.

- You will continue to have a blood sample to check the safety of Truvada® for your kidneys every 6 months. If at any time you have an abnormal result, we will contact you so that you will know and schedule you to return for another visit to recheck the result and evaluate you.

- We will ask you to bring your baby to have a study visit within 1 month of birth, 2 months later and every three months after that until your baby is 1 year old. During these visits, we will do an examination of the baby to collect information on how much the baby is growing.

At any time in the study:

- If you or the study staff thinks you may have any health problems, you may need to undergo a physical exam and may need to provide blood or other samples for testing.
- You can have extra counseling and testing for HIV if needed between scheduled visits, either with your partner or by yourself.
- If you and your partner end your relationship before your last scheduled study visit, we will ask you to stay in the study as originally scheduled.
- If you decide to leave the study before your last scheduled study visit, we will ask you to have a final study visit with all the exams and tests listed above.
- If your pregnancy ends before it is expected to end, we will ask you to come to your next study visit as scheduled.

IF YOU BECOME HIV INFECTED DURING PREGNANCY

During the study we will provide you with condoms, Truvada®, and other materials to help you prevent getting HIV. However it is possible to become HIV infected.

If you have a positive HIV test while pregnant:

- The study staff will talk with you about this test result and what this means for you.
- You will stop the taking the Truvada® medication.
- The staff will ask your permission to obtain a second blood sample [up to 95.5 ml / less than 7 tablespoons] that will be used to confirm the initial positive test and to perform a CD4 count, a measure of your immune function, and to measure the amount of HIV in your blood.
- You will undergo a physical exam
- You will then be asked to return for another visit within about 2 weeks. At that visit, results from the confirmatory HIV test will be available. If those results confirm that you have become infected with HIV, we will ask your permission to obtain another blood sample [up to 95.5 ml / less than 7 tablespoons] and you will be referred to an HIV care clinic.
- You will be referred for prevention of mother to child HIV transmission services at the local health center.

IMPORTANCE OF NOT SHARING THE STUDY MEDICATION

It is very important that you do not share HIV medication with your partner or with anyone else. Truvada® is used to treat HIV infection, but it only works for treating people who already have HIV infection if used in combination with other medications. Thus, Truvada® by itself is only for HIV uninfected people.

SPECIMEN STORAGE AND USE OF SAMPLES AND DATA FOR FUTURE STUDIES

We would like to save data from this study and samples of your blood at [local site] and at the University of Washington for future research by us and by other researchers. We will use these samples only for research related to HIV, HIV-related diseases, and sexually-transmitted infections. Before your samples leave the clinic, they will be assigned a code and your name will not be on them. Your name will be linked to the code only at this clinic and only for five years after the study is completed. After that time, the link between your name and the code on your data and samples will be destroyed. An Institutional Review Board or Independent Ethics Committee, which watches over the safety and rights of research participants, must approve any future research studies using your data and samples. If you agree to store your samples, we will keep them for as long as a sample remains that can be used for future research. If you do not want to have your samples saved for future research, you can still be in this study and your samples will be destroyed once testing for this study is completed. If you agree to store your samples now, but change your mind before the end of the study, let the study staff know and we will make sure that your samples do not get stored for future research. We will not sell your data or samples. Tests done on your samples may lead to a new invention or discovery. We have no plans to share any money or other benefits resulting from this invention or discovery with you.

RISKS AND/OR DISCOMFORTS

You may feel discomfort or pain when your blood is drawn. You may feel dizzy or faint. You may have a bruise where the needle goes into your arm.

You may become embarrassed, worried, or anxious when talking about your sexual practices, ways to protect against HIV and other infections passed during sex, and your HIV test results. You may become worried or anxious while waiting for your test results. If you become infected with HIV, knowing this could make you worried or anxious. Talking about HIV and finding out your test results could cause problems between you and your partner. Trained counselors will help you and your partner deal with any feelings or questions you may have.

The study staff will make every effort to protect your privacy and confidentiality while you are having the study procedures. However, it is possible that others may learn of your participation here and, because of this, may treat you unfairly or discriminate against you. For example, you could have problems getting or keeping a job, or being accepted by your family or community.

Risks potentially related to the Truvada® medication

You may have symptoms or adverse effects while participating in the study. These symptoms or adverse effects may be due to participation in the study or due to illnesses that have no relation to the study, like a cold or flu. All persons who participate in this study will be watched carefully to monitor their health. You should tell the doctor of the study clinic about any symptoms that you feel while you are participating in the study. You will be given a telephone number where the study doctors will be available 24 hours a day, 7 days a week. You should call them if you experience any serious symptoms.

The adverse effects that can occur in a small proportion of people taking Truvada® are well known because the medication has been used by many people. Occasional adverse effects include: mild problems of kidney function that are only detected by laboratory tests; lack of energy/fatigue; upset stomach, vomiting, soft or liquid stools; dizziness. Rare adverse effects include: rash; liver function problems; serious kidney damage; allergic reaction. Small changes in the mineral density of bones were observed in studies of people who were given Truvada®, but the changes in the mineral density of the bones did not cause any fractures, or other problems that bothered the patients. Lactic acidosis has occurred in HIV-infected persons taking Truvada®, in combination with other drugs. Lactic acidosis is a condition that can produce shortness of breath, nausea, and liver failure. This is a serious adverse effect of some medications used for HIV infection. **You should call or come to the study clinic if you have unexplained increased or decreased urination, weight loss, cramps, muscle pain, dizziness, excessive fatigue, nausea, vomiting, or shortness of breath. If you have these symptoms, or any other symptoms that concern you, the study staff will evaluate your symptoms and determine whether you should stop your Truvada® pills.**

Truvada® has been studied in a small number of women during pregnancy and after birth and there are some studies that are still ongoing. Data from animals and people, including studies done in Kenya and Uganda, suggest that Truvada® is safe when used by women who are pregnant. Studies also suggest that Truvada® does not increase the risk of major birth defects for babies. However, not enough studies have been done in pregnant women yet. So, Truvada® should be used during pregnancy only if it is clearly needed. Women thinking about using Truvada® should think about their risk of HIV.

The medications used in this study may have side effects that no one knows about yet. The researchers will let you know if they learn anything that might make you change your mind about continuing to participate in the study.

Risk of acquiring HIV infection and drug resistance
When a woman is pregnant, she may have more risk of getting HIV than usual. You may become infected with HIV during this study from your HIV-infected partner or from other sexual partners you may have. It is very important to use all the known strategies to prevent getting HIV, like using condoms for all sexual relations and keeping your number of sexual partners low. You could become infected with a strain of HIV that could be resistant to Truvada® or other medicines used for HIV treatment. Resistance to antiretroviral medicines may make effective HIV treatment more difficult and may limit your treatment options. You will be able to discuss treatment and the generation of resistance to medicines with the study doctor.
For more information about risks of this study, ask your study doctor.

BENEFITS
You may or may not get direct benefit from being in this study. You or others may benefit in the future from information learned in this study. You also may get some personal satisfaction from being part of research on HIV.

You will be receiving Truvada®, which is known to help keep HIV-uninfected individuals who have HIV-infected partners from getting HIV. You will get counseling and testing for HIV. You will get free condoms. While Truvada® has been shown in research studies to lower the chances of getting HIV, it does not provide 100% protection; using condoms every time you and your partner have sex is important to protect against HIV. If you or your partner have health problems that may be due to infections passed during sex, you will get medicine to treat them, if needed. For other health problems, the study staff will give you care and treatment that is

available at the clinic. For care and treatment that is not available at the clinic, study staff will tell you about other places where care and treatment may be available.

If you become infected with HIV while in this study, you will be offered counseling and referred to HIV care for yourself and services to prevent mother to child HIV transmission to your baby.

After the study is over, the study staff will refer you and your partner to other HIV care programs that are available to you.

NEW FINDINGS
You will be told any new information learned during this study that is important for your health or might cause you to change your mind about staying in the study. You will be told when the results of the study may be available, and how to learn about them.

COSTS TO YOU
There is no cost to you for being in this study. Treatments available to you from the study will be given free of charge.

REASONS WHY YOU MAY BE WITHDRAWN FROM THE STUDY
You may be removed from the study without your consent for the following reasons:

• The study is stopped or canceled.

• The study staff feel that staying in the study would be harmful to you.

REIMBURSEMENT
[*Sites to insert information about local incentives*:] [You will receive [*site specified amount*] for your time and effort at each scheduled visit. You also will receive payment for the costs of [lost work, travel, and/or childcare] due to your visits.][*Note that reimbursement will be only for research procedures, to try to mimic as real-world delivery of PrEP as possible.]*

CONFIDENTIALITY
Efforts will be made to keep your personal information confidential. However, absolute confidentiality cannot be guaranteed. Your personal information may be disclosed if required by law. Any sample from you or information about you will be identified only by code. The link between your name and code will be kept in a secure location at the clinic only. We will not discuss any information about you with your partner unless you give written permission, and we will encourage you to be present during the discussion. Any publication of this study will not use your name or identify you personally.

Your study records may be reviewed by study staff and representatives of:

- the University of Washington, including study monitors
- the Bill and Melinda Gates Foundation
- the United States National Institutes of Health
- The United States Agency for International Development

- [insert applicable local authorities, e.g., Ministry of Health]
- [insert names of applicable IRBs/ECs]

RESEARCH-RELATED INJURY
[Sites to amend this sample text as needed, and/or specify institutional policy]
The study staff will monitor your health while you are in this study. If you have any health problems between visits, please contact the study staff. If you have a medical emergency that requires immediate care, [insert site-specific instructions].

If you are injured from participating in this study, you will be offered care at the study clinic, free of charge. It is important that you tell the members of the team of researchers at this clinic if you feel that you have been injured because of taking part in this study. There is not a program of monetary compensation through this institution. If you require medical care that the study clinic cannot provide, the study doctors will refer you to the appropriate services or organizations that can provide care for the injury. You do not give up any legal rights by signing this consent form.

PROBLEMS OR QUESTIONS
If you ever have any questions about this study, or if you have a research-related injury, you should contact [insert name of the investigator or other study staff] at [insert telephone number and/or physical address].

If you have questions about your rights as a research participant, you should contact [insert name or title of person on the IRB/EC or other organization appropriate for the site] at [insert telephone number and/or physical address of above].

STATEMENT OF CONSENT AND SIGNATURES
I have read this form or had it read to me. I have discussed the information with study staff. My questions have been answered. I understand that my decision whether or not to take part in the study is voluntary. I understand that if I decide to join the study I may withdraw at any time. By signing this form I do not give up any rights that I have as a research participant.

[Insert signature blocks as required by the local IRB/EC:]

______________________	___________________________	_______________
Participant Name (print)	Participant Signature/Thumbprint	Date
______________________	___________________________	_______________
Study Staff Conducting Study Consent Discussion (print)	Staff Signature	Date
______________________	___________________________	_______________
Witness Name (print)	Witness Signature	Date

SPECIMEN STORAGE AND USE OF YOUR DATA AND SAMPLES FOR FUTURE STUDIES:

Please initial and date one option:
___________ I DO agree to store my data and samples for future research into HIV, HIV-related diseases, and other sexually transmitted diseases.

____________ I DO NOT agree to store my data and samples for future research into HIV, HIV-related diseases, and other sexually transmitted diseases.

[Insert signature blocks as required by the local IRB/EC:]

_____________________<br>Participant Name (print)	__________________________<br>Participant Signature/Thumbprint	______________<br>Date
_____________________<br>Study Staff Conducting Consent Discussion (print)	__________________________<br>Study Staff Signature	______________<br>Date
_____________________<br>Witness Name (print)	__________________________<br>Witness Signature	______________<br>Date
