# Supplementary material for: Integrated Delivery of Antiretroviral Treatment and Pre-exposure Prophylaxis to HIV-1–Serodiscordant Couples: A Prospective Implementation Study in Kenya and Uganda
Source: PLoS Med. 2016 Aug 23;13(8):e1002099. doi: 10.1371/journal.pmed.1002099 (PMC4995047; doi:10.1371/journal.pmed.1002099)
Supplement: S1 STROBE Checklist — The STROBE checklist is included as supplementary text. (DOCX) [file pmed.1002099.s003.docx]

STROBE Statement—checklist of items that should be included in reports of observational studies

|  | Item No | Recommendation | Present manuscript |
| --- | --- | --- | --- |
| **Title and abstract** | 1 | (*a*) Indicate the study’s design with a commonly used term in the title or the abstract | **Done (“prospective implementation study” in title).** |
|  |  | (*b*) Provide in the abstract an informative and balanced summary of what was done and what was found | **Done (throughout Abstract and summarized in “Interpretation” section).** |
| Introduction | | |  |
| Background/rationale | 2 | Explain the scientific background and rationale for the investigation being reported | **Done (throughout Introduction).** |
| Objectives | 3 | State specific objectives, including any prespecified hypotheses | **“The overall goal was to evaluate a scalable, integrated, and pragmatic delivery approach for ART and time-limited PrEP, in combination with targeted counseling, brief adherence promotion, and frequency of follow-up designed to reflect approaches suitable for public health settings in East Africa.”** |
| Methods | | |  |
| Study design | 4 | Present key elements of study design early in the paper | **Done (Methods).** |
| Setting | 5 | Describe the setting, locations, and relevant dates, including periods of recruitment, exposure, follow-up, and data collection | **Done (1^st^ paragraph of Methods).** |
| Participants | 6 | (*a*) *Cohort study*—Give the eligibility criteria, and the sources and methods of selection of participants. Describe methods of follow-up  *Case-control study*—Give the eligibility criteria, and the sources and methods of case ascertainment and control selection. Give the rationale for the choice of cases and controls  *Cross-sectional study*—Give the eligibility criteria, and the sources and methods of selection of participants | **Done (2^nd^ paragraph of Methods).** |
|  |  | (*b*) *Cohort study*—For matched studies, give matching criteria and number of exposed and unexposed  *Case-control study*—For matched studies, give matching criteria and the number of controls per case | **N/A** |
| Variables | 7 | Clearly define all outcomes, exposures, predictors, potential confounders, and effect modifiers. Give diagnostic criteria, if applicable | **“Initially-HIV-1 seronegative participants who had positive HIV-1 rapid test results had HIV-1 seroconversion confirmed by enzyme immunoassay and plasma HIV-1 RNA PCR…. For all HIV-1 seroconverters, archived plasma samples from the enrollment visit were tested by HIV-1 RNA PCR and those with detectable HIV-1 RNA, signifying seronegative acute HIV-1 infection, were assessed as having been infected prior to study initiation”** |
| Data sources/ measurement | 8* | For each variable of interest, give sources of data and details of methods of assessment (measurement). Describe comparability of assessment methods if there is more than one group | **As per #7.** |
| Bias | 9 | Describe any efforts to address potential sources of bias | **Detailed in Results.** |
| Study size | 10 | Explain how the study size was arrived at | **“A sample size of 1000 couples was chosen to provide a robust evaluation of the integrated ART and PrEP delivery strategy, across a diversity of clinical research sites.”** |
| Quantitative variables | 11 | Explain how quantitative variables were handled in the analyses. If applicable, describe which groupings were chosen and why | **Detailed in Statistical Analysis section.** |
| Statistical methods | 12 | (*a*) Describe all statistical methods, including those used to control for confounding | **Detailed in Statistical Analysis section.** |
|  |  | (*b*) Describe any methods used to examine subgroups and interactions |  |
|  |  | (*c*) Explain how missing data were addressed |  |
|  |  | (*d*) *Cohort study*—If applicable, explain how loss to follow-up was addressed  *Case-control study*—If applicable, explain how matching of cases and controls was addressed  *Cross-sectional study*—If applicable, describe analytical methods taking account of sampling strategy |  |
|  |  | (*e*) Describe any sensitivity analyses |  |

Continued on next page

| Results | | |  |
| --- | --- | --- | --- |
| Participants | 13* | (a) Report numbers of individuals at each stage of study—eg numbers potentially eligible, examined for eligibility, confirmed eligible, included in the study, completing follow-up, and analysed | **This information is provided in the first paragraph of the Results and in Figure 1.** |
|  |  | (b) Give reasons for non-participation at each stage |  |
|  |  | (c) Consider use of a flow diagram |  |
| Descriptive data | 14* | (a) Give characteristics of study participants (eg demographic, clinical, social) and information on exposures and potential confounders | **This information is provided in the first paragraph of the Results and in Table 1.** |
|  |  | (b) Indicate number of participants with missing data for each variable of interest | **Table 1.** |
|  |  | (c) *Cohort study*—Summarise follow-up time (eg, average and total amount) | **“A total of 858 person-years of follow-up were accrued, with a median follow-up of 11·4 months per couple for assessment of incident HIV-1 infection (interquartile range 6-15). Retention of HIV-1 uninfected partners for assessment of HIV-1 acquisition was ≥90% throughout follow-up.”** |
| Outcome data | 15* | *Cohort study*—Report numbers of outcome events or summary measures over time | **“A total of 14 initially HIV-1 seronegative partners seroconverted to HIV-1 during follow-up, of which 12 were subsequently determined by HIV-1 RNA PCR testing of archived plasma to have been infected at the time of study enrollment. Thus, 2 incident HIV-1 infections occurred, among 1001 couples, for an observed HIV-1 incidence of 0·2 per 100 person-years (Figure 3).”** |
|  |  | *Case-control study—*Report numbers in each exposure category, or summary measures of exposure |  |
|  |  | *Cross-sectional study—*Report numbers of outcome events or summary measures |  |
| Main results | 16 | (*a*) Give unadjusted estimates and, if applicable, confounder-adjusted estimates and their precision (eg, 95% confidence interval). Make clear which confounders were adjusted for and why they were included | **Figure 3** |
|  |  | (*b*) Report category boundaries when continuous variables were categorized | **Figure 3** |
|  |  | (*c*) If relevant, consider translating estimates of relative risk into absolute risk for a meaningful time period | **Figure 3 and related paragraphs in text.** |
| Other analyses | 17 | Report other analyses done—eg analyses of subgroups and interactions, and sensitivity analyses | **Paragraph on subgroups in Results.** |
| Discussion | | |  |
| Key results | 18 | Summarise key results with reference to study objectives | **First paragraph of Discussion.** |
| Limitations | 19 | Discuss limitations of the study, taking into account sources of potential bias or imprecision. Discuss both direction and magnitude of any potential bias | **Second to last paragraph of Discussion.** |
| Interpretation | 20 | Give a cautious overall interpretation of results considering objectives, limitations, multiplicity of analyses, results from similar studies, and other relevant evidence | **Done.** |
| Generalisability | 21 | Discuss the generalisability (external validity) of the study results | **“The empiric risk scoring tool we used to recruit couples for this study and select the counterfactual population was developed and validated in three separate cohorts of HIV-1 serodiscordant couples, from seven African countries and occurring over a decade, suggesting generalizability and stability over time.”** |
| Other information | | |  |
| Funding | 22 | Give the source of funding and the role of the funders for the present study and, if applicable, for the original study on which the present article is based | **“The Partners Demonstration Project was funded by the National Institute of Mental Health of the US National Institutes of Health (grant R01 MH095507), the Bill & Melinda Gates Foundation (grant OPP1056051), and through the generous support of the American people through the US Agency for International Development (cooperative agreement AID-OAA-A-12-00023). Gilead Sciences donated the PrEP medication but had no role in data collection or analysis. The results and interpretation presented here do not necessarily reflect the views of the study funders.”** |

*Give information separately for cases and controls in case-control studies and, if applicable, for exposed and unexposed groups in cohort and cross-sectional studies.

**Note:** An Explanation and Elaboration article discusses each checklist item and gives methodological background and published examples of transparent reporting. The STROBE checklist is best used in conjunction with this article (freely available on the Web sites of PLoS Medicine at http://www.plosmedicine.org/, Annals of Internal Medicine at http://www.annals.org/, and Epidemiology at http://www.epidem.com/). Information on the STROBE Initiative is available at www.strobe-statement.org.
