# Supplementary material for: Integrated Delivery of Antiretroviral Treatment and Pre-exposure Prophylaxis to HIV-1–Serodiscordant Couples: A Prospective Implementation Study in Kenya and Uganda
Source: PLoS Med. 2016 Aug 23;13(8):e1002099. doi: 10.1371/journal.pmed.1002099 (PMC4995047; doi:10.1371/journal.pmed.1002099)
Supplement: S1 Table — Data on serious adverse events and events felt by the treating clinician to be related to PrEP were collected. (DOCX) [file pmed.1002099.s004.docx]

**Supplementary Table 1. Serious adverse events**

|  | **Number of events** |
| --- | --- |
| **HIV-1 uninfected partners** | |
| **Grade 5 (death)** |  |
| Suicide | 1 |
| Unknown cause | 1 |
| **Grade 4** |  |
| Abortion, spontaneous | 4 |
| Intestinal obstruction | 1 |
| Hemoglobin decreased | 1 |
| Suicide attempt | 2 |
| Uterine hemorrhage | 1 |
| **Grade ≤3** |  |
| Abortion spontaneous | 1 |
| Anal abscess | 1 |
| Blood creatinine increased | 1 |
| Caesarean section | 1 |
| Cephalo-pelvic disproportion | 1 |
| Delivery | 6 |
| Fetal death | 1 |
| Fetal distress syndrome | 1 |
| Malaria | 3 |
| Penetrating abdominal trauma | 1 |
| Pneumonia | 1 |
| Premature labor | 1 |
| Road traffic accident | 2 |
| Upper respiratory tract infection | 1 |
| Urogenital fistula | 1 |
| **HIV-1 infected partners** |  |
| **Grade 5 (death)** |  |
| Aids dementia complex | 1 |
| Completed suicide | 1 |
| Dehydration | 1 |
| Diarrhea | 1 |
| Gastric cancer | 1 |
| Lymphoma | 1 |
| Meningitis | 1 |
| Pulmonary tuberculosis | 1 |
| Pyrexia | 1 |
| Rash, papulosquamous | 1 |
| Wound sepsis | 1 |
| **Grade 4** |  |
| Abnormal loss of weight | 2 |
| Abortion incomplete | 1 |
| Abortion spontaneous | 3 |
| Anemia | 1 |
| Diabetes mellitus | 1 |
| Fetal death | 8 |
| Hemoglobin decreased | 1 |
| Pre-eclampsia | 1 |
| Premature delivery | 1 |
| Premature labor | 1 |
| Stab wound | 1 |
| Stillbirth | 2 |
| Suicide attempt | 1 |
| Umbilical cord prolapse | 1 |
| Uterine contractions during pregnancy | 1 |
| Cleft lip | 1 |
| **Grade ≤3** |  |
| Abortion incomplete | 1 |
| Abortion spontaneous | 2 |
| Anemia of pregnancy | 1 |
| Breech presentation | 1 |
| Caesarean section | 7 |
| Cephalo-pelvic disproportion | 1 |
| Chest pain | 1 |
| Delivery (vaginal) | 48 |
| Failed induction of labor | 1 |
| Fetal distress syndrome | 5 |
| Fetal malposition | 1 |
| Fetal malpresentation | 1 |
| Gastritis | 1 |
| Gastroenteritis | 1 |
| Hemorrhoidal hemorrhage | 1 |
| Hemiplegia | 1 |
| Lower limb fracture | 1 |
| Malaria | 4 |
| Meningitis | 1 |
| Meningitis tuberculous | 1 |
| Obstructed labor | 1 |
| Placenta previa | 1 |
| Postpartum sepsis | 1 |
| Premature rupture of membranes | 3 |
| Prolonged labor | 2 |
| Prolonged pregnancy | 1 |
| Respiratory tract infection | 2 |
| Retained placenta or membranes | 1 |
| Soft tissue injury | 1 |
| Twin pregnancy | 1 |
| Typhoid fever | 1 |
| Upper limb fracture | 1 |
| Urinary tract infection | 2 |
| Uterine leiomyoma | 2 |

In the study protocol, serious adverse events and events felt by the treating clinician to be related to PrEP were collected. None of the above events were felt to be related to PrEP. The severity of clinical and laboratory adverse events were scored according to criteria established by the Division of AIDS, National Institute of Allergy and Infectious Diseases of the U.S. National Institutes of Health.
